# Computational Study on Substrate Specificity of a Novel Cysteine Protease 1 Precursor from *Zea mays*

**DOI:** 10.3390/ijms150610459

**Published:** 2014-06-11

**Authors:** Huimin Liu, Liangcheng Chen, Quan Li, Mingzhu Zheng, Jingsheng Liu

**Affiliations:** 1 College of Food Science and Engineering, Jilin Agricultural University, Changchun 130118, China; E-Mails: hymanliu@126.com (H.L.); minminlhm@126.com (M.Z.); 2 National Engineering Laboratory of Wheat and Corn Deep Processing, Jilin Agricultural University, Changchun 130118, China; 3 Engineering Research Center of Edible and Medicinal Fungi, Ministry of Education, Changchun 130118, China; E-Mail: chen71@139.com; 4 College of Life Science, Jilin University, Changchun 130023, China; E-Mail: liquan13@mails.jlu.edu.cn

**Keywords:** homology modeling, molecular dynamics, MM-PBSA, cysteine protease 1

## Abstract

Cysteine protease 1 precursor from *Zea mays* (zmCP1) is classified as a member of the C1A family of peptidases (papain-like cysteine protease) in MEROPS (the Peptidase Database). The 3D structure and substrate specificity of the zmCP1 is still unknown. This study is the first one to build the 3D structure of zmCP1 by computer-assisted homology modeling. In order to determine the substrate specificity of zmCP1, docking study is used for rapid and convenient analysis of large populations of ligand–enzyme complexes. Docking results show that zmCP1 has preference for P1 position and P2 position for Arg and a large hydrophobic residue (such as Phe). Gly147, Gly191, Cys189, and Asp190 are predicted to function as active residues at the S1 subsite, and the S2 subsite contains Leu283, Leu193, Ala259, Met194, and Ala286. *SIF*t results indicate that Gly144, Arg268, Trp308, and Ser311 play important roles in substrate binding. Then Molecular Mechanics-Poisson-Boltzmann Surface Area (MM-PBSA) method was used to explain the substrate specificity for P1 position of zmCp1. This study provides insights into the molecular basis of zmCP1 activity and substrate specificity.

## 1. Introduction

Corn (*Zea mays*) gluten meal (CGM), a by-product of the corn wet-milling process containing approximately 60% protein, is usually used as animal feed rather than food because of its insolubility. China has copious amount of CGM. However, instead of being used in a more efficient way, a great part of it is wasted, creating environmental problems. It was reported previously that the solubility of corn peptides (CPS), the hydrolysate of CGM could be significantly increased [1]. Important bioactivities of CPS have also been reported. For instance, CPS is able to increase ethanol metabolism thus reduce its concentration in blood plasma, and enhance hepatic alcohol dehydrogenase and aldehyde dehydrogenase activities, and so on [2,3].

CPS can be obtained by enzymatic hydrolysis. Proteases are the enzymes that can be used to hydrolyze CGM to generate CPS. Proteases are widely used in food, medicine, detergent, textile and leather processing industries. Papain superfamily is the largest family of proteases that include a wide range of enzymes from both prokaryotes and eukaryotes, encompassing bacteria, plants, invertebrates, and vertebrates [4]. Papain-like cysteine proteinases belong to papain superfamily and are essentially synthesized as inactive proenzymes (zymogens) with *N*-terminal propeptide regions. The most useful feature of propeptides is its ability to inhibit the activity of their cognate peptidases with high selectivity [5,6]. Papain-like cysteine proteinase is classified as a member of the C1A family of peptidases using the MEROPS (the Peptidase Database) search [5,7,8].

Cysteine protease 1 precursor from *Zea mays* (zmCP1) is also a member of the C1A family of peptidases. The 3D structures of several enzymes from the papain superfamily have been determined [9,10,11], and their overall similarity corresponds to the level expected from sequence homologies. The catalytic triad consists of two amino acids-cysteine and histidine. Aspartyl was concluded to play a role analogous to aspartate in the serine protease catalytic triad. However, cysteine protease 1 precursor (zmCP1) from *Zea mays* receives limited investigation. Until now, the 3D structure of cysteine protease 1 precursor remains unknown. It would be useful to find out the binding pose of cysteine protease 1 precursor in order to design excellent mutants for effective hydrolyzation of CGM. In this study, the homology model was built, and molecular dynamics, docking, and Molecular Mechanics-Poisson-Boltzmann Surface Area (MM-PBSA) calculations were used to predict the substrate specificity of zmCP1. Our results contribute more insightful information about C1A family peptidases.

## 2. Results and Discussion

### 2.1. Homology Modeling

Several 3D structures with homologous sequences to cysteine protease 1 (NP_001151293.1) [12] were found by Protein Data Base/Basic Local Alignment Search Tool (PDB/BLAST). Eight templates were used to build the model (Table 1). The 3D structure of zmCP1 was built by Swiss model on line. The Qualitative Model Energy Analysis (QMEAN) server provides a quality estimate on the basis of the geometrical analysis result of a weighted all-against-all comparison of the models from the ensemble provided by the user [13,14]. In many cases, peptide proteinase inhibitors are synthesized as part of a larger precursor protein, either as a propeptide or as an *N*-terminal domain associated with an inactive peptidase or zymogen [15]. This domain prevents the substrate to slide in the active site pocket. Removal of this region by proteolytic cleavage results in activation of the enzyme. For this reason, only the residues from 124 to 340 of 340 residues containing zmCP1 [12] are included in the modeling in this paper (1–123 residues functioned as peptide proteinase inhibitor and do not appear in the mature protein).

**Table 1:** The homology modeling results (made by Swiss model online).

Template (PDB ID)	Sequence Identity	Resolution	Organism	Query Coverage	QMEAN *Z*-Score ^a^	Procheck ^b^	Errat ^c^
1S4V A	59%	2.00	*Ricinus communis*	0.64	−1.26	83.1% core 15.7% allow 0.6% gener 0.6% disall	85.2
2FO5 A	56%	2.20	*Hordeum vulgare*	0.64	−2.01	83.1% core 15.7% allow 0.6% gener 6.0% disall	82.0
3P5W A	55%	1.90	*Actinidia arguta*	0.63	−1.82	82.6% core 15.7% allow 0.6% gener 1.1% disall	82.5
1AEC A	53%	1.86	*Actinidia chinensis*	0.63	−2.38	83.6% core 15.3% allow 0.0% gener 1.1% disall	82.8
1CJL A	44%	2.20	*Homo sapiens*	0.79	−4.32	85.1% core 13.2% allow 0.4% gener 1.3% disall	68.6
1PCI A	43%	3.20	*Carica papaya*	0.90	−5.55	79.3% core 18.8% allow 1.5% gener 0.4% disall	72.6
3TNX A	42%	2.62	*Carica papaya*	0.88	−5.17	81.5% core 16.2% allow 1.5% gener 0.8% disall	70.8
7PCK B	40%	3.20	*Homo sapiens*	0.89	−4.91	78.8% core 17.8% allow 1.5% gener 1.9% disall	63.0

Abbreviations: PDB, protein data base; QMEAN, Qualitative Model Energy Analysis. ^a^ Calculated by Swiss model on line; ^b^ Calculated by Procheck online [16,17]; ^c^ Calculated by Errat online [16,18].

From Table 1, the lowest absolute value of QMEAN *Z*-score was got by the model made of KDEL-tailed cysteine endopeptidase (PDB ID 1S4V A, sequence identify 59%, KDEL represent a unique *C*-terminal sequence which is required for the retention of these proteins in the endoplasmic reticulum) [13]. It is well known that the *Z*-scores of the individual terms of the scoring function are indications of structural features of a model that could exhibit significant deviation from the expected “native” behavior [15]. The “good” models depicted in reach QMEAN *Z*-scores comparable to experimental structures about −0.65 and the “medium” quality models are about −1.75 [15]. QMEAN *Z*-score for our model made by KDEL-tailed cysteine endopeptidase (PDB ID 1S4V A) is −1.26, which is “medium” quality model. Ninety-nine point four percent residues of the model made of KDEL-tailed cysteine endopeptidase (PDB ID 1S4V A) were in the allowed region by PROCHECK methods [17]. The ERRAT [18] score (85.2) of the model made of KDEL-tailed cysteine endopeptidase (PDB ID 1S4V A) was higher than the other models employed in our study. The resolution of the template (PDB ID 1S4V A) is 2.0 Å, and the coverage between the model and the template is 0.64, which suggested that the KDEL-tailed cysteine endopeptidase (PDB ID 1S4V A) is a right template for the 3D structure building for zmCP1.

**Figure 1:**
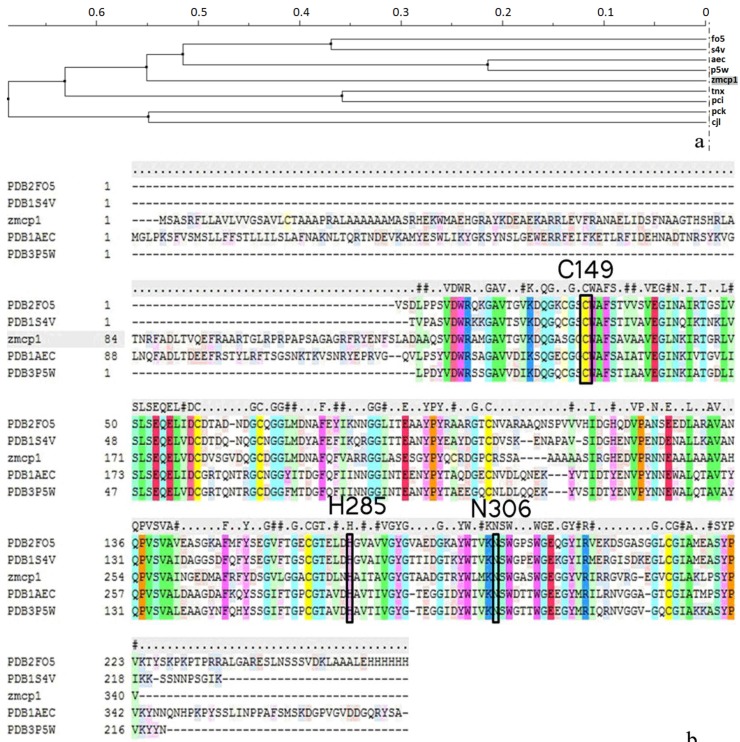
(**a**) Phylogenetic analysis of nine proteins; (**b**) Sequence alignment of zmCP1 and KDEL-tailed cysteine endopeptidase (PDB ID 1S4V, sequence identity 59%), 2FO5, barley cysteine endoprotease B isoform 2 (EP-B2) (PDB ID 2FO5, sequence identity 56%); 3P5W, Actinidin (PDB ID 3P5W, sequence identity 55%); 1AEC: Actinidin (PDB ID 1AEC, sequence identity 53. Red represent identity, and yellow represent similary.

In Figure 1a, zmCP1 and KDEL-tailed cysteine endopeptidase evolved from a common ancestor, hence they may share the similar structure in the active site. It is known that high sequence similarity would warrant the reliability of the homology model. According to the sequence alignment (Figure 1b), the catalytic triad of zmCP1 was conservative, composed by Cys149, His285, and Asn306. In Figure 1b, the highest sequence similarity score is KDEL-tailed cysteine endopeptidase (PDB ID 1S4V A) [13]. High sequence similarity level usually ensures accurate alignment between the target sequence and the template structure. After trying different methods to assess the built models, the model made by KDEL-tailed cysteine endopeptidase was chosen for further study.

**Figure 2:**
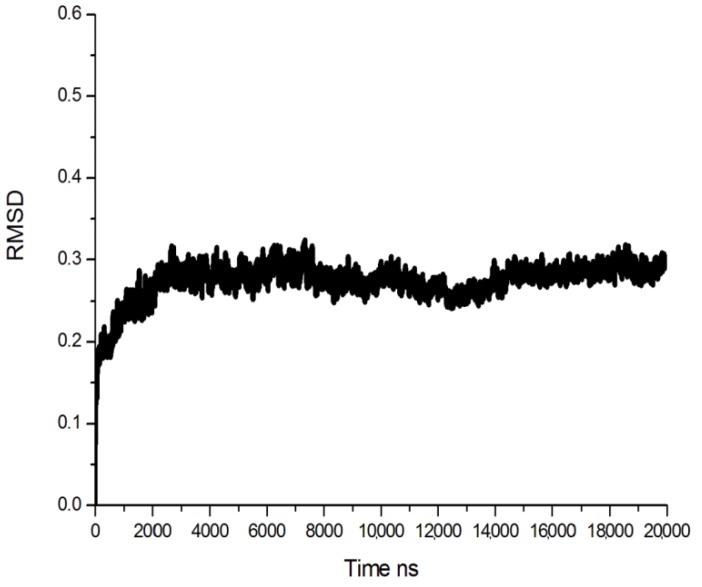
Root-mean-square deviation (RMSD) plot during 20-ns simulation.

The model was minimized using the Amber 10 program with the conditions described later in Experimental section. Figure 2 shows the root-mean-square deviation (RMSD) of Cα atoms to their initial positions during 20 ns molecular dynamics (MD) simulations. The model was stable after 4000 ps. So the last conformation during the 20 ns MD simulation was chosen for the further study.

**Figure 3:**
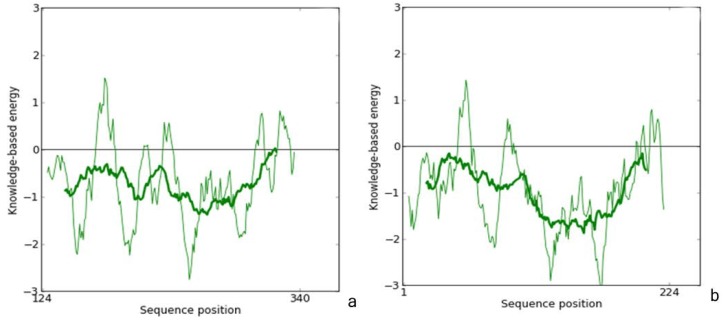
Screen shot of residue of a native protein (**a**) zmCP1; (**b**) PDB ID 1S4V. Thin line represents result of window size 10, thick line represents result of window size 40.

The stereochemistry of the model was assessed using ProSA-web [19], which is a diagnostic tool that is based on the statistical analysis of all available protein structures. The location of the *Z*-score for 1S4V (chain A) is −7.98, and is in the range of native conformations, and the location of the *Z*-score for zmCP1 is −6.95 and is also in the range of native conformation similar to 1S4V. Figure 3 shows the screen shot of residue of a native protein, indicating that the two structures of zmCP1 and 1S4V (chain A) are similar to each other.

**Figure 4:**
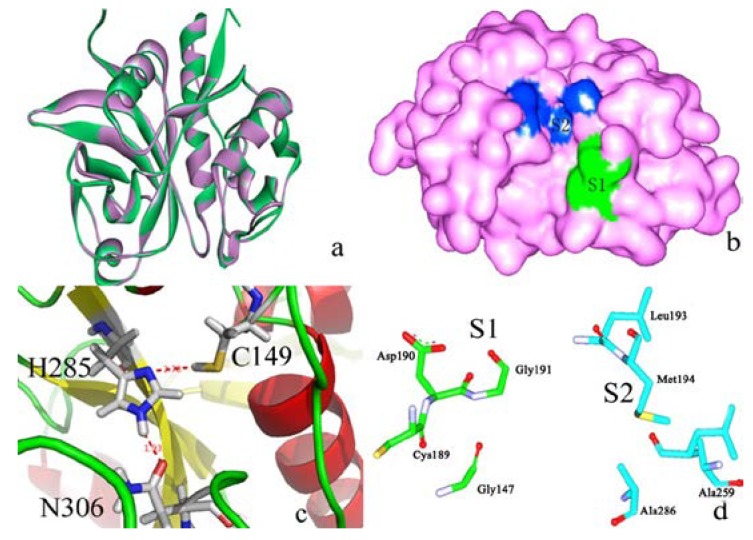
(**a**) Structure superimpose between zmCP1 (green) and the template (purple); (**b**) S1 and S2 pocket in the protein contact potential of zmCP1; (**c**) Catalytic triad of zmCP1: Cys149, His285, and Asn306; (**d**) The S1 (Gly147, Gly191, Cys189, and Asp190) and S2 (Leu193, Met194, Leu283, and Ala286) residues in zmCP1.

Figure 4a shows the superimpose alignment of zmCP1 (green) and KDEL-tailed cysteine endopeptidase (PDB ID 1S4V A) (purple), indicating the similarity in structure (RMSD of Cα atoms 0.56 Å). It was reported that KDEL-tailed cysteine endopeptidase can cleave a protein at multiple sites with strong preference for hydrophobic residues in the P2 position (designating the substrate residues *N*-/*C*-terminal of the scissile peptide bond, P1, P2, … and P1', P2', …, opposing the enzyme specificity pockets S1, S2, … and S1', S2', …, respectively) and no obvious preference in the P1 position [13]. Figure 4b shows the contact potential of S1 and S2 in of zmCP1. In alignment of the amino acid sequences zmCP1 and the KDEL-tailed cysteine endopeptidase shows high degree similarity, especially in their catalytic sites, which can be clearly observed in zmCP1 (Cys149, His285, and Asn306 acted as the catalytic triad, Figure 4c). Figure 4d shows the S1 (Gly147, Gly191, Cys189, and Asp190) and S2 (Leu193, Met194, Leu283, and Ala286) pocket in zmCP1.

### 2.2. Docking Study

Interestingly, cysteine endopeptidase accepts proline at the P1 and P1' positions of the cleavage site [13]. The dominant specificity subsite in most of the C1A subfamily is S2. This commonly displays a preference for occupation by a bulky hydrophobic side chain (such as Phe), and not a charged one [20]. Exceptionally, the S2 subsite of cathepsin B readily accepts Arg; this distinctive specificity of cathepsin B can be explained by the residue lying at the bottom of the S2 pocket [21]. However, the substrate specificity for zmCP1 is still not known. In this study, docking methods were used to determine the preference in the P1 and P2 positions.

#### 2.2.1. Docking Validation

Despite many challenges, docking methods have emerged to be a useful tool in drug discovery and design [22]. A great deal of docking analysis software has been developed for research purposes which makes the later validation and refinement of docking and associated protocols more important. In particular, it is important to pick up how well a given procedure can accurately generate and score known ligand binding poses [23,24].

**Figure 5:**
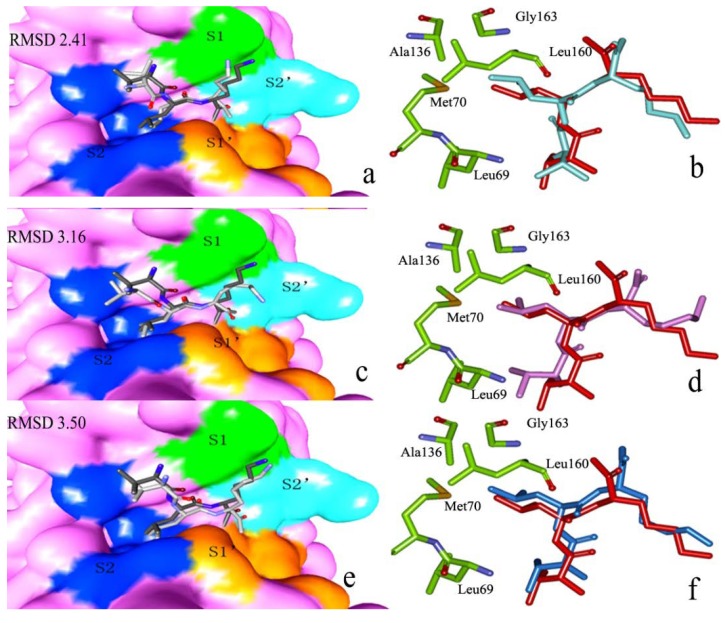
(**a**,**c**,**e**) Surface representation of the substrate binding sites of inhibitor E64. Dark blue color represents S2 pocket, and green color represents S1 pocket, calculated by Autodock Vina (Molecular Graphics Laboratory, La Jolla, CA, USA), Autodock 4.2 (Molecular Graphics Laboratory), Dock 6.6 (University of California, San Francisco, CA, USA), respectively; (**b**,**d**,**f**) The S1, S2, S1', and S2' residues around the docked ligand and the reference for the crystal structure (red),calculated by Autodock Vina, Autodock 4.2, Dock 6.6 , respectively.

Docking success was observed when the top scoring pose was about 2.5 Å heavy atoms RMSD of the crystal ligand. It is important to note that examining docking accuracy depends on the RMSD algorithm employed. When the top-scoring pose was not within 2.5 Å, it was defined as a scoring failure. Figure 5a–f shows representative example for a ligand (inhibitor E64) docked to a target (the template, KDEL-tailed cysteine endopeptidase) with Autodock vina, Autodock 4.2 and Dock 6.6 software. Seen from Figure 5a–f, the docked ligands were in the same orientation in the different binding modes (S1, S2, S1', and S2'). And it is easier to see which part of the ligands docks in which cavity. In comparison to the crystallographic reference, the ligand docked by Autodock vina was successful (RMSD 2.41). Therefore, Autodock vina was used for further docking analysis.

#### 2.2.2. P1–S1 Interactions

**Figure 6:**
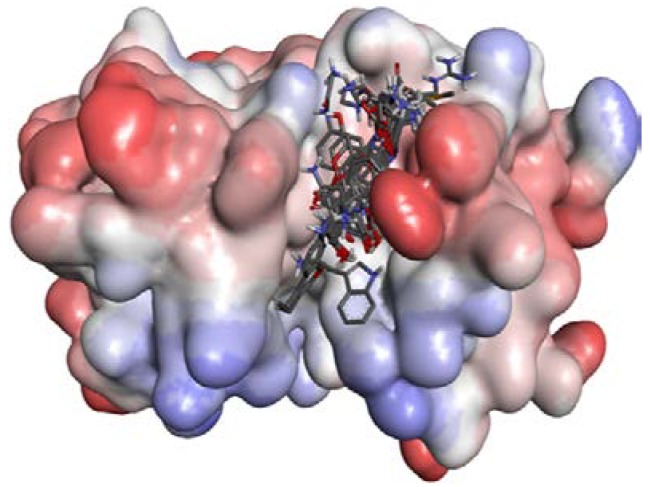
The protein contact potential of zmCP1–ligands complex. Red color represents negative charge; blue represents positive charge; and grey color represents neutral charge.

7-Amido-4-methylcoumarin (AMC) is used as the fluorogenic group. Twenty substrates (see Figure S1) were drawn by Chemdraw 3D (CambridgeSoft, Cambridge, MA, USA.) and then optimized with Gaussian 03 B3LYP methods at 6-31G* set. The ligands were docked to zmCP1 with Autodock vina. In Figure 6, it can be seen that all 20 ligands locate in the active cleft.

Autodock vina will provide 20 docking poses for a ligand docking. In general, there are several conformations with the lowest energy score. During these conformations with the lowest energy score, the correct binding mode for each ligand is selected as follow: firstly, the P1 group is in the S1 binding pocket (Gly147, Gly191, Cys189, and Asp190) according to sequence alignment between zmCP1 and KDEL-tailed cysteine endopeptidase (PDB ID 1S4V A); secondly, the distance between the carbon of hydroxyl group of R'-AMC and sulfur should be in about 4 Å; lastly, the positive charge at P1 should make hydrogen with the oxyanion hole residues.

The docking scores are listed in Table 2. The lowest docking score among the 20 ligands is −10.4 kcal·mol^−1^ (R-AMC–zmCP1, a difference of 2.1 kcal·mol^−1^ between the first two substrates). The kinetic studies indicate that the papain-like cysteine proteases have a preference for a long-chain positively charged residue such as Arg, Lys, and His at the P1 position [13]. In our docking study, the docking score between R-AMC and zmCP1 (−10.4 kcal·mol^−1^) is lower than that of H-AMC (−6.4) and K-AMC (−6.1). It can be concluded that zmCP1 has a preference for P1 for Arg.

**Table 2:** The docking scores among the 20 peptides and zmCP1 (kcal·mol^−1^).

Ligands	Docking Score	Ligands	Docking Score	Ligands	Docking Score
R-AMC	−10.4	L-AMC	−6.4	I-AMC	−6.1
F-AMC	−8.3	V-AMC	−6.4	K-AMC	−6.1
Y-AMC	−7.9	A-AMC	−6.2	T-AMC	−6.1
W-AMC	−7.5	Q-AMC	−6.2	M-AMC	−5.9
N-AMC	−7.2	E-AMC	−6.2	S-AMC	−5.9
P-AMC	−6.9	G-AMC	−6.2	D-AMC	−5.5
H-AMC	−6.4	C-AMC	−6.1	–	–

**Figure 7:**
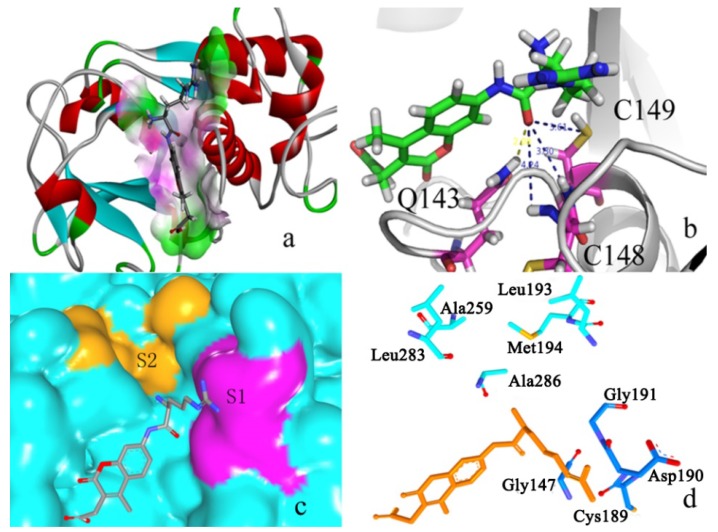
(**a**) R-AMC in the active binding pocket calculated by Discovery studio client 3.5 (Accelrys, San Diego, CA, USA); (**b**) The hydrogen bonds among the R-AMC and the active residues; (**c**) Surface representation of the substrate binding sites of R-AMC; (**d**) The active sites of R-AMC. Light blue color represents S2 pocket residues; dark blue color represents S1 pocket residues; orange color represents R-AMC.

Figure 7a shows that R-AMC, the best substrate locates in the active pocket. The distance between the carbon of hydroxyl group of R-AMC and sulfur is 4.33 Å, and it is useful for the nucleophile to attach. It was reported that in 1S4V [13] and 2FO5 [25], Gln20, Ser25, and Cys26 (residue number according to PDB ID 1S4V) function as oxyanion hole, while in 1AEC [26], only Gln20, and Cys26 (residue number according to 1S4V) function as oxyanion hole. According to sequence alignment, Cys149, Cys148, and Gln143 function as the oxyanion hole in zmCP1. Shown in Figure 1b, Cys149 and Gln143 are conservative in all five C1A proteins, but not Cys148. During the docking cycle, the positive charge at Arg P1 is stabilized via its tight interaction with Gln143 N^ε2^ forming (2.08 Å, Figure 7b), together with the main chain nitrogen atoms of Cys149 (3.80 Å), and the nitrogen atoms of Cys148 of the main chain does not make a hydrogen bond with R-AMC. So Cys149 and Gln143 may act as the oxyanion hole for zmCP1. Figure 7c,d indicate that the P1 group locate at S1 binding pocket.

#### 2.2.3. P2–S2 Interactions

It was reported that the S2 pocket in C1A family proteases is of special interest, because it is an established fact that the specificity of this family enzymes is determined predominantly by P2–S2 interactions [13,27]. In our study, AMC was used as the fluorogenic group and Arg as the P1 residue. 20 substrates were drawn by Chemdraw 3D and then optimized with Gaussian 03 at B3LYP 6-31G* set. The ligands are docked to zmCP1 with Autodock vina.

Autodock vina will provide 20 docking poses for a ligand docking. In general, there are several conformations with the lowest energy score. During these conformations with the lowest energy score, the correct binding mode for each ligand is selected as follow: firstly, the P1 group is in the S1 binding pocket (Gly147, Gly191, Cys189, and Asp190) and P2 group is in the S2 binding pocket (Leu283, Leu193, Ala259, Met194, and Ala286); secondly, the distance between the carbon of hydroxyl group of R'-AMC and sulfur should be in about 4 Å; lastly, the positive charge at P1 should make hydrogen with the oxyanion hole residues.

**Figure 8:**
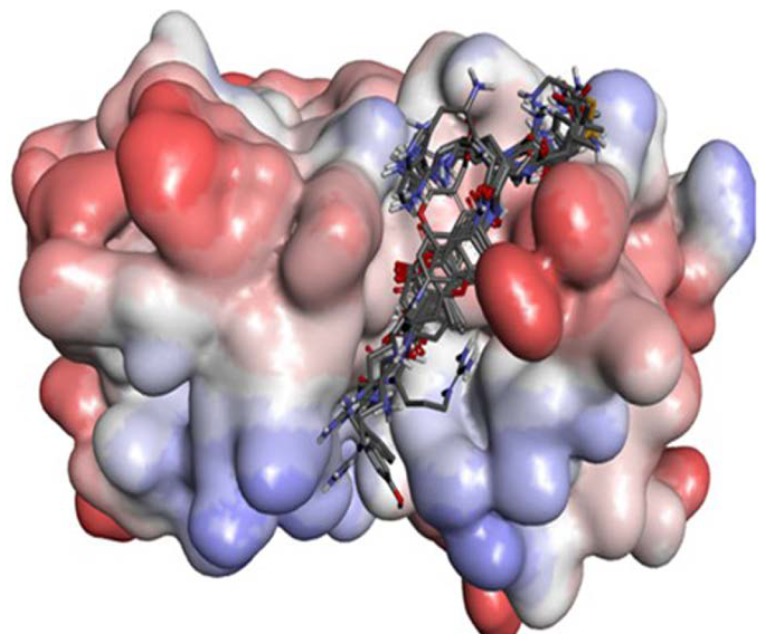
The protein contact potential of zmCP1–ligands complex. Red color represents negative charge; blue represents positive charge; and grey color represents neutral charge.

In Figure 8, it can be seen that all 20 ligands locate in the active cleft. The docking scores are listed in Table 3.

**Table 3:** The docking scores among the 20 dipeptides and zmCP1 (kcal·mol^−1^).

Ligands	Docking Score	Ligands	Docking Score	Ligands	Docking Score
F-R-AMC	−9.8	A-R-AMC	−8.3	E-R-AMC	−8.0
Y-R-AMC	−9.7	N-R-AMC	−8.3	T-R-AMC	−8.0
P-R-AMC	−8.9	I-R-AMC	−8.3	G-R-AMC	−7.8
H-R-AMC	−8.5	D-R- AMC	−8.2	K-R-AMC	−7.6
R-R-AMC	−8.4	Q-R-AMC	−8.2	M-R-AMC	−7.6
W-R-AMC	−8.4	L-R-AMC	−8.1	S-R-AMC	−7.6
V-R-AMC	−8.4	C-R-AMC	−8.1	–	–

The lowest docking score among the 20 ligands is −9.8 kcal·mol^−1^ (F-R-AMC–zmCP1). This result indicates that the enzyme has specificity for aromatic or non-polar residues (such as Phe and Tyr) at the P2 position of the peptide substrate. The crystal structure of zmCP1 complexes with different substrate analog inhibitors reveals that the P1 side chain faces the solvent, whereas the P2 side chain contacts the enzyme surface inside an enclosed cavity, the S2 subsites which in papain is dominantly hydrophobic in nature [27,28,29]. Our result is consistent with the existing data [21].

**Figure 9:**
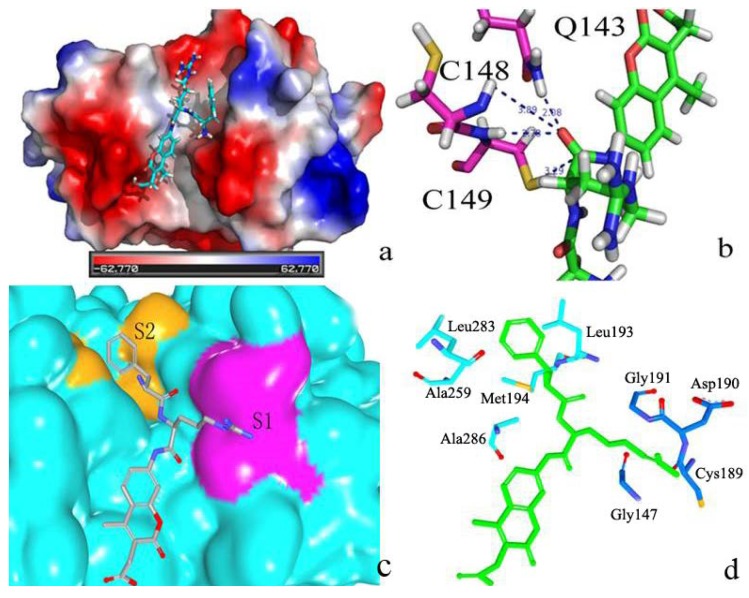
(**a**) F-R-AMC in the active cleft. Red color represents negative charge; blue represents positive charge; and grey color represents neutral charge; (**b**) The hydrogen bonds among the F-R-AMC and the active residues in the active binding pocket calculated by Discovery studio client 3.5.; (**c**) Surface representation of the substrate binding sites of F-R-AMC; (**d**) The active sites of F-R-AMC. Light blue color represents S2 pocket residues; dark blue color represents S1 pocket residues; green color represents F-R-AMC.

Figure 9a shows that F-R-AMC locates in the active cleft. Seen from Figure 9b, the distance between the carbon of hydroxyl group of F-R-AMC and sulfur is 3.29 Å, it is useful to the nucleophile to attach. During the docking cycle, the positive charge at Arg P1 is stabilized via its tight interaction with Gln143 N^ε2^ forming (2.08 Å, Figure 9b), together with the main chain nitrogen atoms of Cys149 (2.78 Å). The main chain nitrogen atoms of Cys148 also do not make a hydrogen bond with F-R-AMC.

As for the S2 subsite, zmCP1 is also dominantly hydrophobic in nature. The S2 pocket is the major determinant of specificity for most cysteine proteinases [13]. KDEL-tailed cysteine endopeptidase (PDB ID 1S4V) is formed mostly by Leu69, Ala136, Gly163, Leu160, and Met170 located at the edge of the S2 (see Figure 5b,d,f). In our study, the S2 residues of zmCP1 containing Leu193, Leu283, Met194, and Ala259 form a hydrophobic cavity, then it is more prone that it facilitates more the binding of hydrophobic residues (such as Phe) than of positively charged or polar ones (Figure 9c,d).

### 2.3. Structural Interaction Fingerprint Analysis

Analysis of their binding modes with different molecules is an approach to determine the composition and volume of the binding sites of a protein. It can be seen from Figure 6 and Figure 8 that all ligands are located in the same active cleft. Then structural interaction fingerprint analysis (*SIF*t) was performed over an ensemble of protein–ligand complexes. The procedure allowed the finding out of crucial amino acids involved in interactions with ligands. The *SIF*t results are listed in Table 4.

**Table 4:** An averaged structural interaction fingerprint (*SIF*t) calculated over all successfully docked poses among two receptor conformations and 40 ligands presented for all identified interacting residues.

Amino Acid	Interactions								
	Any	Back-Bone	Side Chain	Polar	Hydrophobic	H-Bond Acceptor	H-Bond Donor	Aromatic	Charged
Q143	1	0.1	1	0.97	0	0	0.71	0	0
W308	1	0.68	1	1	0	0	0.71	1	0
H285	0.97	0.65	0.97	0.97	0	0	0.1	0	0
M194	0.94	0	0.94	0	0.94	0	0	0	0
L283	0.94	0.71	0.94	0.74	0.19	0	0	0	0
G144	0.9	0.9	0	0.35	0.55	0	0	0	0
R268	0.9	0.9	0.97	0.87	0.1	0.19	0	0	0.71
A286	0.86	0.86	0.86	0.23	0	0	0	0	0
L193	0.74	0.71	0.74	0.23	0.52	0	0	0	0
G147	0.72	0.72	0	0.59	0.13	0	0	0	0
S311	0.68	0.55	0.68	0.55	0.19	0.1	0	0	0
D190	0.56	0.56	0.16	0.46	0.1	0.16	0	0	0
C189	0.56	0.56	0	0.1	0.46	0	0	0	0
G191	0.56	0.56	0	0.53	0	0	0	0	0
A286	0.56	0.56	0	0.56	0	0	0	0	0
C149	0.52	0.29	0.9	0.39	0.52	0	0.13	0	0
N284	0.45	0.45	0.19	0.42	0	0.1	0	0	0
D142	0.32	0	0.32	0.32	0	0.1	0	0	0
Y270	0.29	0.29	0	0.26	0	0.13	0	0	0
W150	0.26	0.26	0.26	0.19	0	0	0	0	0
G262	0.26	0.26	0	0.1	0.16	0	0	0	0
D271	0.23	0.23	0.23	0.1	0.16	0	0	0	0

Cys149, His285, and Gln143 were recognized by *SIF*t, they are important residues popularly responsible for ligand binding (Table 4). Cys149 and His285 function as catalytic triad, and Cys149 and Gln143 act as oxyanion hole. In our study, the S2 residues of zmCP1 contain Leu283, Leu193, Ala259, Met194, and Ala286 and the S1 residues of zmCP1 contain Gly147, Gly191, Cys189, and Asp190.

All ligands feature polar interactions with the Trp308 and Gln143 side chain (Table 4 columns: any, side chain, and polar). Some compounds (71%) interact also with the side chain of Gln143 and Trp308 through a hydrogen bond. Some compounds (71%) interact also with the side chain of Arg268 with charge interaction. All ligands interact also with the side chain of Trp308 with aromatic interactions. Our results may be cross-validated with results from reported mutagenesis studies [30]. And thence *SIF*t results indicated that Gly144, Arg268, Trp308, and Ser311 were important in substrate binding.

### 2.4. Calculation of the Free Energy of Binding with Molecular Mechanics-Poisson–Boltzmann Surface Area (MM-PBSA) Method

Through the docking scores among the 40 ligands and zmCP1 (see Table 2 and Table 3), R-AMC was the best substrate, and the D-AMC is the worst. Thus, R-AMC and D-AMC were chosen to be calculated with MM-PBSA. The 3D structure of R-AMC and D-AMC were optimized with Gaussian 03 at B3LYP 6-31G* set. In general, the larger the apparent catalytic efficiency, the greater affinity an enzyme will have to its substrate. A smaller attractive energy means less affinity between the substrate and enzyme. In order to explain the reason of substrate specificity of zmCP1, we chose R-AMC and D-AMC for further calculations on zmCP1.

**Figure 10:**
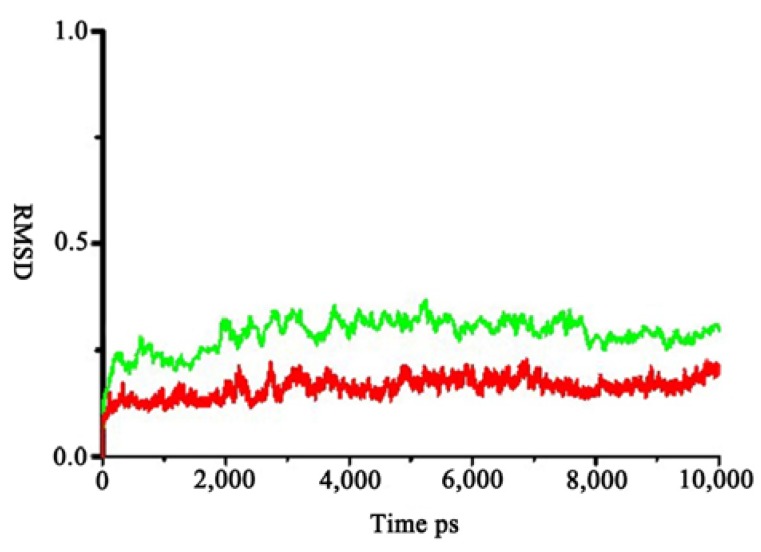
The two RMSD plot during 10 ns simulation (green: zmCP1-D-AMC; red: zmCP1–R-AMC).

To make a more precise and quantitative analysis of the protein–substrate interaction, two complexes were used as starting structures for 10 ns MD simulations. The substrate interactions observed in the starting structures were maintained after the MD simulations. By analyzing the RMSD from the complex structures of all of the heavy atoms of the proteins, it is found that the RMSD remained approximately constant after 10 ns (Figure 10). Seen from Figure 10, the average RMSD for zmCP1–R-AMC complex around 0.22 nm is lower than that of zmCP1-D-AMC complex (0.25 nm), indicating that zmCP1-R-AMC complex is more stable.

The MD trajectories were further analyzed using MM-PBSA method. The two substrates used in the present study are very similar. According to previous studies [31,32,33], the entropy differences should be very small so that the correlation between the experimental *Km* value and the calculated binding free energy may not be greatly improved. Therefore, the solute entropy term was neglected in the present study. For each MD-simulated complex, we calculated the Δ*G*_bind_ values for the 1000 snapshots of the MD trajectory (one snapshot for each 2 ps during the last 2000 ps of the stable trajectory) and the final Δ*G*_bind_ value was the average of the calculated Δ*G*_bind_ values for these snapshots. The binding free energies for the two complexes were estimated (Table 5) using the MM-PBSA method.

**Table 5:** The MM-PBSA score for R-AMC–zmCP1 complex and D-AMC–zmCP1 complex.

Energy Components (kcal·mol^−1^)	R-AMC–zmCP1	D-AMC–zmCP1
*∆E_ele_*	−172.62	−106.88
*∆E_vdW_*	−33.5	−31.4
*∆G_PB_*	170.65	129.89
*∆G_np_*	−5.44	−5.46
Non-polar	−38.94	−36.86
Polar	−1.97	23.01
*∆G_bind_*	−40.91	−13.85

The binding free energies for the two complexes were estimated (Table 5) using the MM-PBSA method. The calculated mean binding free energies were −13.85 kcal·mol^−1^ for D-AMC and zmCP1. The MM-PBSA calculation predicted the R-AMC binds stronger to the zmCP1 (−40.91 kcal·mol^−1^). This result shows that the binding free energy of R-AMC to zmCP1 is 26.06 kcal·mol^−1^ indicating that zmCP1 is more favored than D-AMC. Hence, it can be concluded that zmCP1 has specificity for a positive residue (such as Arg) at the P1 position of the peptide substrate.

## 3. Experimental Section

### 3.1. Homology Protein Modeling

Several 3D structures with homologous sequences to cysteine protease 1 [12] were found by PDB/BLAST. Eight templates were used to build the model (Table 1). The 3D structure of zmCP1 was built by Swiss model on line. Homology models were produced by the Comparative Modeling module in the Swiss model to produce reasonably good models [14,34]. Five steps were used in the protein modeling: (i) sequence alignment on one or more template structures; (ii) threading for generation of initial models based on template structure by copying coordinates over the aligned regions; (iii) loop modeling to rebuild the missing parts using de novo modeling; (iv) selection of models based on reported experimental data from biochemical, biophysical, and electrophysiological studies; (v) refinement using all-atom MD simulations with reported constraints for the interatomic distances of the salt-bridge interaction pair obtained from electrophysiology and mutagenesis experiments.

### 3.2. Molecular Dynamics (MD) Simulation

For the ligand, Generalized AMBER force field (GAFF) parameters and RESP partial charges were assigned using the ANTECH Amber program implemented in Amber 10 [35]. The simulations were done in a truncated octahedral box under periodic boundary conditions and then neutralized with Cl^−^ counterions where necessary. Amber99sb force field was used for the protein–ligand complex. Prior to MD simulations, systems were energy minimized through the steepest descent algorithm with 2000 steps to avoid any steric conflicts generated during the initial setup. The density of the system was adjusted during the first equilibration runs at NPT (Quantum simulations in the isothermic-isobaric) condition by weak coupling to a bath of constant pressure (P0 = 1 bar, coupling time = 2 ps). For temperature regulation, we used Langevin thermostat (NTT = 3) to maintain the temperature of our system at 300 K. This temperature control method uses Langevin dynamics with a collision frequency of 1.0 ps (GAMMA_LN = 1.0) [35]. As such, especially with explicit solvent dynamics, it is often better to equilibrate the system using Langevin methods (NTT = 3) [36] and then, once equilibrated, switch to Berendsen methods (NTT = 1) [37]. The electrostatic interactions were calculated by using the Particle-Mesh Ewald (PME) algorithm [38]. The equilibration procedure consisted of thermalization of the solvent, for 500 ps at 300 K, followed by minimization of all solute atoms, keeping the solvent coordinates fixed, and then started MD simulation of the complete system by raising the temperature from 0 to 300 K in 500 ps increments of 50 K each. Data production was carried out for 10 ns for the two protein-ligands complex and 10 ns for the protein 10 under normal temperature (300 K) and pressure (1 bar), using a temperature coupling time constant of 0.1 ps and a pressure coupling time constant of 2.0 ps. The value of the isothermal compressibility was set to 4.5 × 10^−5^ bar for water simulations. Usually, the catalytic pair Cys–His in the papain-like proteases acts as an ion pair, *i.e.*, Cys(−)–His(+), but in this paper, the catalytic pair Cys149–His285 were not protonated in H++ program calculation.

### 3.3. Docking Study

In validation analysis, AutoDock 4.2 [39,40,41], AutoDock vina [42,43,44], and Dock 6.6 [45] were used to perform docking analysis.

AutoDock 4.2 combines a rapid energy evaluation through precalculated grids of affinity potentials with a variety of search algorithms to find the best-fit binding positions for a ligand to a given protein [40,41,42]. All torsion angles for each compound were considered flexible. The grid maps representing the proteins in the actual docking process were calculated with AutoGrid. The grids (one for each atom type in the ligand plus one for electrostatic interactions) chosenwere sufficiently large enough to include not only active site but also significant portions of the surrounding surface [42].

AutoDock Vina is a new open-source program for drug discovery, molecular docking and virtual screening, offering multi-core capability, high performance and enhanced accuracy and ease of use [43,44,45].

Dock 6.0 improves the algorithm’s ability to predict binding poses by adding new features like force-field scoring enhanced by solvation and receptor flexibility [46]. The created clusters were enclosed in a box, and force fields scoring grids were generated by the GRID module of Dock 6.0. The ligands were docked by optimizing overlap with the active-site spheres. The maximum number of orientations of the ligand was limited to 5000, and only the 50 lowest solutions were saved and evaluated.

### 3.4. Structural Interaction Fingerprint Analysis

Protein residues are grouped into four classes: polar, hydrophobic, aromatic, and charged. In our study, nine bits (any, backbone, side chain, polar, hydrophobic, H-donor, H-acceptor, aromatic, charged) were used to describe those associations.

For each ligand’s atom, the residues within cut-off range were selected. The occurrence of interaction was determined by atom–atom distance, type of atoms/residues, and appropriate angle in case of hydrogen bonds:

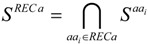
(1)


On this basis an average *SIF*t may generate a population of ligands and/or receptors (e.g., alternative conformational states):

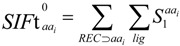
(2)


The list was then sequentially recalculated for every amino acid in the population of ligands docked into receptors, comparing alternative complexes. At this stage only “any contact” bit was taken into account. The most frequent matches (by default, cutoff of 50% was applied) were then put into a separated list for the construction of averaged/consensus fingerprint:

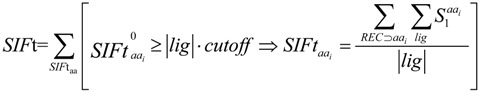
(3)


### 3.5. MM-PBSA Calculations

Ten nanosecond MD were used for two protein-substrate complex analysis. The 2000 snapshots isolated from the final 4000 ps MD trajectory with protein–substrate complex were used for the binding free energy calculation by using the MM-PBSA method encoded in the AMBER 10 program [35]. For each snapshot, the ligand–protein binding free energy (∆*G*_binding_) was calculated using (Equation (4)):

∆*G*_binding_ = ∆*G*_complex_ − [*∆G*_protein_ + ∆*G*_ligand_] (4)

where ∆*G*_complex,_ ∆*G*_protein_ and ∆*G*_ligand_ are the free energies of the complex (protein and ligand). Each free energy term in Equation (1) was calculated with the absolute free energy of the species (protein, ligand, and their complex) in gas phase (*E*_gas_), the solvation free energy (*G*_solvation_), and the entropy term (*TS*) using (Equation (5)):

*G* = *E*_gas_ + *G*_solvation_ − *TS* (5)

*E*_gas_ is the sum of the internal strain energy (*E*_int_), van der Waals energy (*E*_vdW_), and electrostatic energy (*E*_ele_ (Equation (6))). *E*_int_ is the energy associated with vibrations of covalent bonds and bond angles, rotation of single bond torsional angles (Equation (7)):

*E*_gas_ = *E*_int_ + *E*_vdW_ + *E*_ele_ (6)

*E*_int_ = *E*_bond_ + *E*_angle_ + *E*_torsion_**(7)

The solvation free energy, ∆*G*_solvation_, is approximated as the sum of the polar contribution (∆*G*_PB_) and nonpolar contribution (∆*G*_nonpolar_) using a continuum representation of the solvent.

## 4. Conclusions

In this study, we built the 3D structure based on the known amino acids sequence of cysteine protease 1 from *Zea mays*. We report a computer-assisted homology study conducted to build its 3D structure based on the known sequence of amino acids of this enzyme. Docking results show that zmCP1 has preference for P1 and P2 for Arg and a large hydrophobic residue (such as Phe). And *SIF*t results also indicate that Gly144, Arg268, Trp308, and Ser311 are important in substrate binding. MM-PBSA was used to explain the substrate specificity for P1 position of zmCP1. Our findings would provide useful information for further C1A family research.

## Supplementary Files

Supplementary File 1 Supplementary Information (PDF, 719 KB)
