# Supplementary material for: Computational Study on Substrate Specificity of a Novel Cysteine Protease 1 Precursor from Zea mays
Source: Int J Mol Sci. 2014 Jun 11;15(6):10459–78. doi: 10.3390/ijms150610459 (PMC4100162; doi:10.3390/ijms150610459)
Supplement: Supplementary File 1 — Supplementary Information (PDF, 719 KB) [file ijms-15-10459-s001.pdf]

Supplementary Information

1. The Coordinates of the Model

TITLE SWISS-MODEL SERVER (http://swissmodel.expasy.org)
TITLE 2 Untitled Project
EXPDTA THEORETICAL MODEL (SWISS-MODEL SERVER)
AUTHOR SWISS-MODEL SERVER (SEE REFERENCE IN JRNL Records)
REVDAT 1 06-MAY-14 1MOD 1 03:24
JRNL AUTH K.ARNOLD, L.BORDOLI, J.KOPP, T.SCHWEDE
JRNL TITL SWISS-MODEL WORKSPACE: A WEB-BASED ENVIRONMENT FOR PROTEIN
JRNL TITL 2 STRUCTURE HOMOLOGY MODELLING
JRNL REF BIOINFORMATICS V. 22 195 2006
JRNL REFN ISSN 1367-4803
JRNL PMID 16301204
JRNL DOI 10.1093/bioinformatics/bti770
REMARK 1
REMARK 1 REFERENCE 1
REMARK 1 AUTH P.BENKERT, M.BIASINI, T.SCHWEDE
REMARK 1 TITL TOWARD THE ESTIMATION OF THE ABSOLUTE QUALITY OF INDIVIDUAL
REMARK 1 TITL 2 PROTEIN STRUCTURE MODELS
REMARK 1 REF BIOINFORMATICS V. 3 343 2011
REMARK 1 REFN ISSN 1367-4803
REMARK 1 PMID 21134891
REMARK 1 DOI 10.1093/bioinformatics/btq662
REMARK 1
REMARK 1 REFERENCE 2
REMARK 1 AUTH F.KIEFER, K.ARNOLD, M.KUENZLI, L.BORDOLI, T.SCHWEDE
REMARK 1 TITL THE SWISS-MODEL REPOSITORY AND ASSOCIATED RESOURCES
REMARK 1 REF NUCLEIC.ACIDS.RES V. 37 D387 2009
REMARK 1 REFN ISSN 0305-1048
REMARK 1 PMID 18931379
REMARK 1 DOI 10.1093/nar/gkn750
REMARK 1
REMARK 1
REMARK 1 REFERENCE 3
REMARK 1 AUTH T.SCHWEDE, J.KOPP, N.GUEX, M.C.PEITSCH
REMARK 1 TITL SWISS_MODEL: AN AUTOMATED PROTEIN HOMOLOGY-MODELING
REMARK 1 TITL 2 SERVER
REMARK 1 REF NUCLEIC.ACIDS.RES. V. 31 3381 2003
REMARK 1 REFN ISSN 0305-1048
REMARK 1 PMID 12824332
REMARK 1 DOI 10.1093/nar/gkg520
REMARK 1

REMARK 1 REFERENCE 4
REMARK 1 AUTH M.C.PEITSCH, N.GUEX
REMARK 1 TITL SWISS-MODEL AND THE SWISS-PDBVIEWER: AN ENVIRONMENT
REMARK 1 TITL 2 FOR COMPARATIVE PROTEIN MODELLING
REMARK 1 REF ELECTROPHORESIS V. 18 2714 1997
REMARK 1 REFN ISSN
REMARK 1 DOI 10.1002/elps.1150181505
REMARK 1 PMID 9504803
REMARK 1
REMARK 1 REFERENCE 5
REMARK 1 AUTH M.C.PEITSCH
REMARK 1 TITL PROTEIN MODELING BY E-MAIL
REMARK 1 REF BIO/TECHNOLOGY V. 13 658 1995
REMARK 1 REFN ISSN 1087-0156
REMARK 1 DOI 10.1038/nbt0795-658
REMARK 1
REMARK 1 DISCLAIMER
REMARK 1 The SWISS-MODEL SERVER produces theoretical models for proteins.
REMARK 1 The results of any theoretical modelling procedure is
REMARK 1 NON-EXPERIMENTAL and MUST be considered with care. These models may
REMARK 1 contain significant errors. This is especially true for automated
REMARK 1 modeling since there is no human intervention duringmodel
REMARK 1 building. Please read the header section and the logfile carefully
REMARK 1 to know what templates and alignments were used during the model
REMARK 1 building process. All information by the SWISS-MODELSERVER is
REMARK 1 provided "AS-IS", without any warranty, expressed orimplied.
REMARK 2
REMARK 2 COPYRIGHT NOTICE
REMARK 2 This SWISS-MODEL protein model is copyright. It is produced by the
REMARK 2 SWISS-MODEL Server Group at Swiss Institute of Bioinformatics. There
REMARK 2 are no restrictions on the use of individual models for your or your
REMARK 2 employer's research projects. If you publish or patent any results
REMARK 2 obtained from this model, please cite the papers mentioned under
REMARK 2 JRNL.
REMARK 2
REMARK 2 FAIR USE NOTICE
REMARK 2 Downloading the entire data-base or substantial portions of it,
REMARK 2 systematic or automatic submission of data, mirroring the
REMARK 2 repository, or further redistribution of substantialportions data
REMARK 2 obtained from SWISS-MODEL is prohibited and requireswritten
REMARK 2 permission from the SWISS-MODEL Server Group at Swiss Institute of
REMARK 2 Bioinformatics.

REMARK 3
REMARK 3 MODEL INFORMATION
REMARK 3 ENGIN PROMOD
REMARK 3 VERSN 3.70
REMARK 3 OSTAT MONOMER
REMARK 3 OSRSN USER
REMARK 3 GMQE NA
REMARK 3 QMN4 -1.26
REMARK 3
REMARK 3 TEMPLATE 1
REMARK 3 PDBID 1s4v
REMARK 3 CHAIN A
REMARK 3 MMCIF A
REMARK 3 PDBV 04-04-2014
REMARK 3 SMTLE 1s4v.1.A
REMARK 3 SMTLV 09-04-14
REMARK 3 MTHD X-RAY DIFFRACTION 2.00 A
REMARK 3 FOUND HHblits
REMARK 3 GMQE 0.57
REMARK 3 SIM 0.49
REMARK 3 SID 58.80
REMARK 3 OSTAT monomer
REMARK 3 LIGND DVA-LEU-LYS-0QE
ATOM 1 N ASP A 124 22.257 29.647 22.354 1.00 0.44 N
ATOM 2 CA ASP A 124 21.712 30.942 22.801 1.00 0.44 C
ATOM 3 C ASP A 124 20.891 31.624 21.689 1.00 0.44 C
ATOM 4 O ASP A 124 21.441 32.240 20.778 1.00 0.44 O
ATOM 5 CB ASP A 124 22.842 31.865 23.284 1.00 0.44 C
ATOM 6 CG ASP A 124 22.338 33.236 23.763 1.00 0.44 C
ATOM 7 OD1ASP A 124 21.101 33.416 23.848 1.00 0.44 O
ATOM 8 OD2ASP A 124 23.207 34.066 24.098 1.00 0.44 O
ATOM 9 N ALA A 125 19.577 31.503 21.823 1.00 0.65 N
ATOM 10 CA ALA A 125 18.642 32.171 20.905 1.00 0.65 C
ATOM 11 C ALA A 125 17.996 33.346 21.643 1.00 0.65 C
ATOM 12 O ALA A 125 17.670 33.239 22.828 1.00 0.65 O
ATOM 13 CB ALA A 125 17.575 31.171 20.453 1.00 0.65 C
ATOM 14 N ALA A 126 17.848 34.455 20.924 1.00 0.74 N
ATOM 15 CA ALA A 126 17.194 35.663 21.462 1.00 0.74 C
ATOM 16 C ALA A 126 15.799 35.302 22.000 1.00 0.74 C
ATOM 17 O ALA A 126 15.167 34.346 21.547 1.00 0.74 O
ATOM 18 CB ALA A 126 17.073 36.718 20.356 1.00 0.74 C
ATOM 19 N GLN A 127 15.356 36.062 22.990 1.00 0.67 N

ATOM	20	CA	GLN	A	127	14.014	35.903	23.590	1.00	0.67	C
ATOM	21	C	GLN	A	127	12.905	35.954	22.524	1.00	0.67	C
ATOM	22	O	GLN	A	127	11.916	35.231	22.618	1.00	0.67	O
ATOM	23	CB	GLN	A	127	13.749	36.957	24.678	1.00	0.67	C
ATOM	24	CG	GLN	A	127	13.637	38.417	24.205	1.00	0.67	C
ATOM	25	CD	GLN	A	127	14.937	39.039	23.677	1.00	0.67	C
ATOM	26	OE1	GLN	A	127	16.041	38.520	23.747	1.00	0.67	O
ATOM	27	NE2	GLN	A	127	14.825	40.235	23.162	1.00	0.67	N
ATOM	28	N	SER	A	128	13.106	36.799	21.512	1.00	0.83	N
ATOM	29	CA	SER	A	128	12.147	36.959	20.407	1.00	0.83	C
ATOM	30	C	SER	A	128	12.827	37.501	19.151	1.00	0.83	C
ATOM	31	O	SER	A	128	13.841	38.198	19.215	1.00	0.83	O
ATOM	32	CB	SER	A	128	11.019	37.917	20.803	1.00	0.83	C
ATOM	33	OG	SER	A	128	11.525	39.232	21.051	1.00	0.83	O
ATOM	34	N	VAL	A	129	12.260	37.097	18.024	1.00	0.86	N
ATOM	35	CA	VAL	A	129	12.715	37.537	16.691	1.00	0.86	C
ATOM	36	C	VAL	A	129	11.475	37.853	15.849	1.00	0.86	C
ATOM	37	O	VAL	A	129	10.532	37.068	15.803	1.00	0.86	O
ATOM	38	CB	VAL	A	129	13.580	36.450	16.019	1.00	0.86	C
ATOM	39	CG1	VAL	A	129	14.044	36.870	14.622	1.00	0.86	C
ATOM	40	CG2	VAL	A	129	14.831	36.121	16.843	1.00	0.86	C
ATOM	41	N	ASP	A	130	11.512	39.013	15.208	1.00	0.86	N
ATOM	42	CA	ASP	A	130	10.466	39.432	14.269	1.00	0.86	C
ATOM	43	C	ASP	A	130	11.144	39.863	12.963	1.00	0.86	C
ATOM	44	O	ASP	A	130	11.522	41.024	12.809	1.00	0.86	O
ATOM	45	CB	ASP	A	130	9.650	40.572	14.895	1.00	0.86	C
ATOM	46	CG	ASP	A	130	8.446	40.985	14.041	1.00	0.86	C
ATOM	47	OD1	ASP	A	130	8.487	40.761	12.808	1.00	0.86	O
ATOM	48	OD2	ASP	A	130	7.506	41.549	14.629	1.00	0.86	O
ATOM	49	N	TRP	A	131	11.180	38.945	12.006	1.00	0.81	N
ATOM	50	CA	TRP	A	131	11.783	39.208	10.684	1.00	0.81	C
ATOM	51	C	TRP	A	131	11.112	40.331	9.886	1.00	0.81	C
ATOM	52	O	TRP	A	131	11.769	40.999	9.089	1.00	0.81	O
ATOM	53	CB	TRP	A	131	11.857	37.930	9.850	1.00	0.81	C
ATOM	54	CG	TRP	A	131	12.856	36.957	10.472	1.00	0.81	C
ATOM	55	CD1	TRP	A	131	12.581	35.780	11.029	1.00	0.81	C
ATOM	56	CD2	TRP	A	131	14.220	37.170	10.599	1.00	0.81	C
ATOM	57	NE1	TRP	A	131	13.703	35.242	11.505	1.00	0.81	N
ATOM	58	CE2	TRP	A	131	14.720	36.064	11.263	1.00	0.81	C
ATOM	59	CE3	TRP	A	131	15.062	38.208	10.220	1.00	0.81	C
ATOM	60	CZ2	TRP	A	131	16.073	35.996	11.557	1.00	0.81	C
ATOM	61	CZ3	TRP	A	131	16.419	38.130	10.502	1.00	0.81	C

ATOM	62	CH2	TRP	A	131	16.926	37.023	11.173	1.00	0.81	C
ATOM	63	N	ARG	A	132	9.843	40.603	10.184	1.00	0.73	N
ATOM	64	CA	ARG	A	132	9.118	41.740	9.575	1.00	0.73	C
ATOM	65	C	ARG	A	132	9.724	43.070	10.038	1.00	0.73	C
ATOM	66	O	ARG	A	132	10.152	43.874	9.218	1.00	0.73	O
ATOM	67	CB	ARG	A	132	7.647	41.759	9.969	1.00	0.73	C
ATOM	68	CG	ARG	A	132	6.907	40.483	9.579	1.00	0.73	C
ATOM	69	CD	ARG	A	132	5.500	40.527	10.159	1.00	0.73	C
ATOM	70	NE	ARG	A	132	5.571	40.621	11.624	1.00	0.73	N
ATOM	71	CZ	ARG	A	132	4.535	40.749	12.445	1.00	0.73	C
ATOM	72	NH1	ARG	A	132	3.287	40.754	11.997	1.00	0.73	N
ATOM	73	NH2	ARG	A	132	4.748	40.884	13.739	1.00	0.73	N
ATOM	74	N	ALA	A	133	9.920	43.166	11.353	1.00	0.78	N
ATOM	75	CA	ALA	A	133	10.572	44.317	12.008	1.00	0.78	C
ATOM	76	C	ALA	A	133	12.056	44.438	11.626	1.00	0.78	C
ATOM	77	O	ALA	A	133	12.580	45.543	11.527	1.00	0.78	O
ATOM	78	CB	ALA	A	133	10.439	44.187	13.527	1.00	0.78	C
ATOM	79	N	MET	A	134	12.690	43.293	11.374	1.00	0.73	N
ATOM	80	CA	MET	A	134	14.098	43.232	10.931	1.00	0.73	C
ATOM	81	C	MET	A	134	14.255	43.599	9.439	1.00	0.73	C
ATOM	82	O	MET	A	134	15.364	43.710	8.920	1.00	0.73	O
ATOM	83	CB	MET	A	134	14.668	41.844	11.251	1.00	0.73	C
ATOM	84	CG	MET	A	134	16.189	41.769	11.048	1.00	0.73	C
ATOM	85	SD	MET	A	134	17.141	42.965	12.054	1.00	0.73	S
ATOM	86	CE	MET	A	134	17.038	42.159	13.637	1.00	0.73	C
ATOM	87	N	GLY	A	135	13.106	43.744	8.767	1.00	0.75	N
ATOM	88	CA	GLY	A	135	13.026	44.136	7.349	1.00	0.75	C
ATOM	89	C	GLY	A	135	13.404	43.016	6.374	1.00	0.75	C
ATOM	90	O	GLY	A	135	13.847	43.303	5.267	1.00	0.75	O
ATOM	91	N	ALA	A	136	13.078	41.775	6.732	1.00	0.76	N
ATOM	92	CA	ALA	A	136	13.308	40.616	5.846	1.00	0.76	C
ATOM	93	C	ALA	A	136	12.011	39.992	5.298	1.00	0.76	C
ATOM	94	O	ALA	A	136	12.020	38.912	4.707	1.00	0.76	O
ATOM	95	CB	ALA	A	136	14.143	39.574	6.600	1.00	0.76	C
ATOM	96	N	VAL	A	137	10.894	40.702	5.453	1.00	0.75	N
ATOM	97	CA	VAL	A	137	9.586	40.208	4.983	1.00	0.75	C
ATOM	98	C	VAL	A	137	8.924	41.262	4.088	1.00	0.75	C
ATOM	99	O	VAL	A	137	8.808	42.430	4.452	1.00	0.75	O
ATOM	100	CB	VAL	A	137	8.677	39.836	6.173	1.00	0.75	C
ATOM	101	CG1	VAL	A	137	7.369	39.193	5.706	1.00	0.75	C
ATOM	102	CG2	VAL	A	137	9.358	38.856	7.132	1.00	0.75	C
ATOM	103	N	THR	A	138	8.430	40.778	2.957	1.00	0.74	N

ATOM	104	CA	THR	A	138	7.672	41.611	2.001	1.00	0.74	C
ATOM	105	C	THR	A	138	6.199	41.716	2.430	1.00	0.74	C
ATOM	106	O	THR	A	138	5.777	41.057	3.381	1.00	0.74	O
ATOM	107	CB	THR	A	138	7.784	41.039	0.580	1.00	0.74	C
ATOM	108	OG1	THR	A	138	7.212	39.731	0.545	1.00	0.74	O
ATOM	109	CG2	THR	A	138	9.232	41.009	0.079	1.00	0.74	C
ATOM	110	N	GLY	A	139	5.419	42.447	1.623	1.00	0.77	N
ATOM	111	CA	GLY	A	139	3.967	42.601	1.842	1.00	0.77	C
ATOM	112	C	GLY	A	139	3.250	41.245	1.781	1.00	0.77	C
ATOM	113	O	GLY	A	139	3.718	40.304	1.140	1.00	0.77	O
ATOM	114	N	VAL	A	140	2.143	41.160	2.508	1.00	0.79	N
ATOM	115	CA	VAL	A	140	1.275	39.966	2.498	1.00	0.79	C
ATOM	116	C	VAL	A	140	0.636	39.855	1.106	1.00	0.79	C
ATOM	117	O	VAL	A	140	0.066	40.812	0.582	1.00	0.79	O
ATOM	118	CB	VAL	A	140	0.207	40.057	3.604	1.00	0.79	C
ATOM	119	CG1	VAL	A	140	−0.750	38.858	3.593	1.00	0.79	C
ATOM	120	CG2	VAL	A	140	0.866	40.124	4.984	1.00	0.79	C
ATOM	121	N	LYS	A	141	0.648	38.632	0.603	1.00	0.75	N
ATOM	122	CA	LYS	A	141	0.084	38.320	−0.718	1.00	0.75	C
ATOM	123	C	LYS	A	141	−1.206	37.507	−0.560	1.00	0.75	C
ATOM	124	O	LYS	A	141	−1.545	37.028	0.524	1.00	0.75	O
ATOM	125	CB	LYS	A	141	1.092	37.502	−1.524	1.00	0.75	C
ATOM	126	CG	LYS	A	141	2.512	38.069	−1.633	1.00	0.75	C
ATOM	127	CD	LYS	A	141	2.560	39.495	−2.192	1.00	0.75	C
ATOM	128	CE	LYS	A	141	3.989	39.900	−2.568	1.00	0.75	C
ATOM	129	NZ	LYS	A	141	4.920	39.775	−1.438	1.00	0.75	N
ATOM	130	N	ASP	A	142	−1.968	37.458	−1.645	1.00	0.78	N
ATOM	131	CA	ASP	A	142	−3.180	36.634	−1.709	1.00	0.78	C
ATOM	132	C	ASP	A	142	−3.062	35.701	−2.917	1.00	0.78	C
ATOM	133	O	ASP	A	142	−3.082	36.155	−4.061	1.00	0.78	O
ATOM	134	CB	ASP	A	142	−4.414	37.540	−1.816	1.00	0.78	C
ATOM	135	CG	ASP	A	142	−5.734	36.778	−1.648	1.00	0.78	C
ATOM	136	OD1	ASP	A	142	−5.771	35.583	−2.022	1.00	0.78	O
ATOM	137	OD2	ASP	A	142	−6.690	37.412	−1.160	1.00	0.78	O
ATOM	138	N	GLN	A	143	−2.950	34.410	−2.629	1.00	0.75	N
ATOM	139	CA	GLN	A	143	−2.846	33.376	−3.676	1.00	0.75	C
ATOM	140	C	GLN	A	143	−4.072	33.304	−4.604	1.00	0.75	C
ATOM	141	O	GLN	A	143	−3.957	32.773	−5.700	1.00	0.75	O
ATOM	142	CB	GLN	A	143	−2.547	32.006	−3.061	1.00	0.75	C
ATOM	143	CG	GLN	A	143	−3.674	31.477	−2.171	1.00	0.75	C
ATOM	144	CD	GLN	A	143	−3.295	30.129	−1.567	1.00	0.75	C
ATOM	145	OE1	GLN	A	143	−2.627	30.026	−0.550	1.00	0.75	O

ATOM	146	NE2	GLN	A	143	−3.675	29.058	−2.223	1.00	0.75	N
ATOM	147	N	GLY	A	144	−5.219	33.813	−4.137	1.00	0.77	N
ATOM	148	CA	GLY	A	144	−6.491	33.799	−4.885	1.00	0.77	C
ATOM	149	C	GLY	A	144	−6.995	32.365	−5.094	1.00	0.77	C
ATOM	150	O	GLY	A	144	−6.676	31.456	−4.327	1.00	0.77	O
ATOM	151	N	ALA	A	145	−7.727	32.182	−6.190	1.00	0.77	N
ATOM	152	CA	ALA	A	145	−8.310	30.877	−6.562	1.00	0.77	C
ATOM	153	C	ALA	A	145	−7.325	29.983	−7.341	1.00	0.77	C
ATOM	154	O	ALA	A	145	−7.539	29.583	−8.486	1.00	0.77	O
ATOM	155	CB	ALA	A	145	−9.611	31.125	−7.333	1.00	0.77	C
ATOM	156	N	SER	A	146	−6.221	29.675	−6.673	1.00	0.77	N
ATOM	157	CA	SER	A	146	−5.180	28.785	−7.213	1.00	0.77	C
ATOM	158	C	SER	A	146	−4.404	28.182	−6.042	1.00	0.77	C
ATOM	159	O	SER	A	146	−3.971	28.878	−5.118	1.00	0.77	O
ATOM	160	CB	SER	A	146	−4.226	29.558	−8.121	1.00	0.77	C
ATOM	161	OG	SER	A	146	−3.573	30.517	−7.316	1.00	0.77	O
ATOM	162	N	GLY	A	147	−4.401	26.848	−6.027	1.00	0.86	N
ATOM	163	CA	GLY	A	147	−3.741	26.072	−4.958	1.00	0.86	C
ATOM	164	C	GLY	A	147	−2.216	26.146	−5.079	1.00	0.86	C
ATOM	165	O	GLY	A	147	−1.539	25.138	−5.268	1.00	0.86	O
ATOM	166	N	CYS	A	148	−1.681	27.339	−4.854	1.00	0.87	N
ATOM	167	CA	CYS	A	148	−0.228	27.567	−4.961	1.00	0.87	C
ATOM	168	C	CYS	A	148	0.380	28.030	−3.630	1.00	0.87	C
ATOM	169	O	CYS	A	148	1.467	28.605	−3.605	1.00	0.87	O
ATOM	170	CB	CYS	A	148	0.020	28.610	−6.043	1.00	0.87	C
ATOM	171	SG	CYS	A	148	−0.838	30.123	−5.517	1.00	0.87	S
ATOM	172	N	CYS	A	149	−0.252	27.642	−2.524	1.00	0.85	N
ATOM	173	CA	CYS	A	149	0.259	27.952	−1.172	1.00	0.85	C
ATOM	174	C	CYS	A	149	1.715	27.492	−0.979	1.00	0.85	C
ATOM	175	O	CYS	A	149	2.534	28.235	−0.444	1.00	0.85	O
ATOM	176	CB	CYS	A	149	−0.620	27.298	−0.109	1.00	0.85	C
ATOM	177	SG	CYS	A	149	−0.614	25.466	−0.135	1.00	0.85	S
ATOM	178	N	TRP	A	150	2.055	26.365	−1.610	1.00	0.83	N
ATOM	179	CA	TRP	A	150	3.429	25.822	−1.610	1.00	0.83	C
ATOM	180	C	TRP	A	150	4.459	26.798	−2.202	1.00	0.83	C
ATOM	181	O	TRP	A	150	5.540	26.976	−1.642	1.00	0.83	O
ATOM	182	CB	TRP	A	150	3.480	24.490	−2.365	1.00	0.83	C
ATOM	183	CG	TRP	A	150	3.076	24.627	−3.837	1.00	0.83	C
ATOM	184	CD1	TRP	A	150	1.833	24.577	−4.308	1.00	0.83	C
ATOM	185	CD2	TRP	A	150	3.924	24.839	−4.915	1.00	0.83	C
ATOM	186	NE1	TRP	A	150	1.852	24.740	−5.631	1.00	0.83	N
ATOM	187	CE2	TRP	A	150	3.110	24.904	−6.034	1.00	0.83	C

ATOM	188	CE3	TRP	A	150	5.304	24.970	−5.043	1.00	0.83	C
ATOM	189	CZ2	TRP	A	150	3.673	25.095	−7.287	1.00	0.83	C
ATOM	190	CZ3	TRP	A	150	5.867	25.151	−6.302	1.00	0.83	C
ATOM	191	CH2	TRP	A	150	5.051	25.211	−7.425	1.00	0.83	C
ATOM	192	N	ALA	A	151	4.053	27.500	−3.261	1.00	0.87	N
ATOM	193	CA	ALA	A	151	4.895	28.498	−3.947	1.00	0.87	C
ATOM	194	C	ALA	A	151	5.117	29.749	−3.082	1.00	0.87	C
ATOM	195	O	ALA	A	151	6.250	30.193	−2.906	1.00	0.87	O
ATOM	196	CB	ALA	A	151	4.260	28.884	−5.286	1.00	0.87	C
ATOM	197	N	PHE	A	152	4.041	30.202	−2.436	1.00	0.83	N
ATOM	198	CA	PHE	A	152	4.081	31.358	−1.514	1.00	0.83	C
ATOM	199	C	PHE	A	152	4.886	31.080	−0.238	1.00	0.83	C
ATOM	200	O	PHE	A	152	5.695	31.883	0.220	1.00	0.83	O
ATOM	201	CB	PHE	A	152	2.668	31.804	−1.141	1.00	0.83	C
ATOM	202	CG	PHE	A	152	2.014	32.580	−2.284	1.00	0.83	C
ATOM	203	CD1	PHE	A	152	1.379	31.905	−3.313	1.00	0.83	C
ATOM	204	CD2	PHE	A	152	2.058	33.969	−2.298	1.00	0.83	C
ATOM	205	CE1	PHE	A	152	0.808	32.625	−4.351	1.00	0.83	C
ATOM	206	CE2	PHE	A	152	1.470	34.672	−3.341	1.00	0.83	C
ATOM	207	CZ	PHE	A	152	0.840	34.001	−4.366	1.00	0.83	C
ATOM	208	N	SER	A	153	4.749	29.865	0.272	1.00	0.88	N
ATOM	209	CA	SER	A	153	5.545	29.433	1.433	1.00	0.88	C
ATOM	210	C	SER	A	153	7.047	29.450	1.115	1.00	0.88	C
ATOM	211	O	SER	A	153	7.834	30.035	1.857	1.00	0.88	O
ATOM	212	CB	SER	A	153	5.115	28.031	1.835	1.00	0.88	C
ATOM	213	OG	SER	A	153	5.991	27.526	2.841	1.00	0.88	O
ATOM	214	N	ALA	A	154	7.389	28.903	−0.051	1.00	0.88	N
ATOM	215	CA	ALA	A	154	8.783	28.827	−0.527	1.00	0.88	C
ATOM	216	C	ALA	A	154	9.408	30.214	−0.739	1.00	0.88	C
ATOM	217	O	ALA	A	154	10.479	30.503	−0.202	1.00	0.88	O
ATOM	218	CB	ALA	A	154	8.832	28.034	−1.834	1.00	0.88	C
ATOM	219	N	VAL	A	155	8.635	31.109	−1.355	1.00	0.84	N
ATOM	220	CA	VAL	A	155	9.096	32.477	−1.669	1.00	0.84	C
ATOM	221	C	VAL	A	155	9.401	33.285	−0.401	1.00	0.84	C
ATOM	222	O	VAL	A	155	10.372	34.033	−0.368	1.00	0.84	O
ATOM	223	CB	VAL	A	155	8.096	33.214	−2.580	1.00	0.84	C
ATOM	224	CG1	VAL	A	155	6.766	33.463	−1.889	1.00	0.84	C
ATOM	225	CG2	VAL	A	155	8.612	34.573	−3.049	1.00	0.84	C
ATOM	226	N	ALA	A	156	8.600	33.085	0.637	1.00	0.85	N
ATOM	227	CA	ALA	A	156	8.769	33.858	1.871	1.00	0.85	C
ATOM	228	C	ALA	A	156	10.110	33.555	2.547	1.00	0.85	C
ATOM	229	O	ALA	A	156	10.848	34.475	2.894	1.00	0.85	O

ATOM	230	CB	ALA	A	156	7.613	33.489	2.762	1.00	0.85	C
ATOM	231	N	ALA	A	157	10.495	32.280	2.485	1.00	0.87	N
ATOM	232	CA	ALA	A	157	11.790	31.807	3.002	1.00	0.87	C
ATOM	233	C	ALA	A	157	12.978	32.319	2.167	1.00	0.87	C
ATOM	234	O	ALA	A	157	13.973	32.784	2.722	1.00	0.87	O
ATOM	235	CB	ALA	A	157	11.778	30.281	3.033	1.00	0.87	C
ATOM	236	N	VAL	A	158	12.794	32.362	0.846	1.00	0.83	N
ATOM	237	CA	VAL	A	158	13.850	32.821	−0.084	1.00	0.83	C
ATOM	238	C	VAL	A	158	14.027	34.346	−0.003	1.00	0.83	C
ATOM	239	O	VAL	A	158	15.149	34.837	0.107	1.00	0.83	O
ATOM	240	CB	VAL	A	158	13.584	32.360	−1.530	1.00	0.83	C
ATOM	241	CG1	VAL	A	158	14.708	32.796	−2.476	1.00	0.83	C
ATOM	242	CG2	VAL	A	158	13.497	30.834	−1.617	1.00	0.83	C
ATOM	243	N	GLU	A	159	12.908	35.067	0.028	1.00	0.76	N
ATOM	244	CA	GLU	A	159	12.926	36.537	0.181	1.00	0.76	C
ATOM	245	C	GLU	A	159	13.591	36.943	1.505	1.00	0.76	C
ATOM	246	O	GLU	A	159	14.406	37.865	1.529	1.00	0.76	O
ATOM	247	CB	GLU	A	159	11.512	37.115	0.168	1.00	0.76	C
ATOM	248	CG	GLU	A	159	10.804	36.924	−1.173	1.00	0.76	C
ATOM	249	CD	GLU	A	159	9.375	37.470	−1.153	1.00	0.76	C
ATOM	250	OE1	GLU	A	159	8.752	37.465	−0.069	1.00	0.76	O
ATOM	251	OE2	GLU	A	159	8.915	37.843	−2.256	1.00	0.76	O
ATOM	252	N	GLY	A	160	13.354	36.095	2.518	1.00	0.89	N
ATOM	253	CA	GLY	A	160	13.894	36.257	3.880	1.00	0.89	C
ATOM	254	C	GLY	A	160	15.421	36.136	3.910	1.00	0.89	C
ATOM	255	O	GLY	A	160	16.125	37.125	4.124	1.00	0.89	O
ATOM	256	N	LEU	A	161	15.902	34.943	3.563	1.00	0.80	N
ATOM	257	CA	LEU	A	161	17.350	34.648	3.562	1.00	0.80	C
ATOM	258	C	LEU	A	161	18.137	35.603	2.650	1.00	0.80	C
ATOM	259	O	LEU	A	161	19.244	36.004	3.003	1.00	0.80	O
ATOM	260	CB	LEU	A	161	17.590	33.177	3.189	1.00	0.80	C
ATOM	261	CG	LEU	A	161	19.059	32.747	3.326	1.00	0.80	C
ATOM	262	CD1	LEU	A	161	19.134	31.244	3.557	1.00	0.80	C
ATOM	263	CD2	LEU	A	161	19.864	33.029	2.055	1.00	0.80	C
ATOM	264	N	ASN	A	162	17.510	36.050	1.560	1.00	0.76	N
ATOM	265	CA	ASN	A	162	18.147	36.988	0.618	1.00	0.76	C
ATOM	266	C	ASN	A	162	18.435	38.342	1.282	1.00	0.76	C
ATOM	267	O	ASN	A	162	19.556	38.836	1.236	1.00	0.76	O
ATOM	268	CB	ASN	A	162	17.284	37.185	−0.630	1.00	0.76	C
ATOM	269	CG	ASN	A	162	17.978	38.122	−1.620	1.00	0.76	C
ATOM	270	OD1	ASN	A	162	19.063	37.861	−2.119	1.00	0.76	O
ATOM	271	ND2	ASN	A	162	17.393	39.281	−1.825	1.00	0.76	N

ATOM	272	N	LYS	A	163	17.445	38.860	2.000	1.00	0.73	N
ATOM	273	CA	LYS	A	163	17.589	40.135	2.723	1.00	0.73	C
ATOM	274	C	LYS	A	163	18.601	40.013	3.869	1.00	0.73	C
ATOM	275	O	LYS	A	163	19.399	40.913	4.098	1.00	0.73	O
ATOM	276	CB	LYS	A	163	16.226	40.567	3.271	1.00	0.73	C
ATOM	277	CG	LYS	A	163	16.278	41.889	4.047	1.00	0.73	C
ATOM	278	CD	LYS	A	163	16.704	43.006	3.111	1.00	0.73	C
ATOM	279	CE	LYS	A	163	16.732	44.425	3.656	1.00	0.73	C
ATOM	280	NZ	LYS	A	163	17.872	44.518	4.563	1.00	0.73	N
ATOM	281	N	ILE	A	164	18.577	38.873	4.549	1.00	0.74	N
ATOM	282	CA	ILE	A	164	19.515	38.590	5.655	1.00	0.74	C
ATOM	283	C	ILE	A	164	20.975	38.603	5.163	1.00	0.74	C
ATOM	284	O	ILE	A	164	21.868	39.053	5.879	1.00	0.74	O
ATOM	285	CB	ILE	A	164	19.147	37.256	6.337	1.00	0.74	C
ATOM	286	CG1	ILE	A	164	17.756	37.373	6.983	1.00	0.74	C
ATOM	287	CG2	ILE	A	164	20.208	36.820	7.366	1.00	0.74	C
ATOM	288	CD1	ILE	A	164	17.217	36.064	7.571	1.00	0.74	C
ATOM	289	N	ARG	A	165	21.185	38.102	3.951	1.00	0.72	N
ATOM	290	CA	ARG	A	165	22.544	37.953	3.401	1.00	0.72	C
ATOM	291	C	ARG	A	165	23.038	39.191	2.640	1.00	0.72	C
ATOM	292	O	ARG	A	165	24.202	39.567	2.757	1.00	0.72	O
ATOM	293	CB	ARG	A	165	22.638	36.697	2.527	1.00	0.72	C
ATOM	294	CG	ARG	A	165	21.718	36.760	1.309	1.00	0.72	C
ATOM	295	CD	ARG	A	165	21.918	35.566	0.403	1.00	0.72	C
ATOM	296	NE	ARG	A	165	21.026	35.664	−0.762	1.00	0.72	N
ATOM	297	CZ	ARG	A	165	20.964	34.745	−1.716	1.00	0.72	C
ATOM	298	NH1	ARG	A	165	21.740	33.667	−1.658	1.00	0.72	N
ATOM	299	NH2	ARG	A	165	20.140	34.910	−2.741	1.00	0.72	N
ATOM	300	N	THR	A	166	22.130	39.837	1.915	1.00	0.79	N
ATOM	301	CA	THR	A	166	22.501	40.940	1.004	1.00	0.79	C
ATOM	302	C	THR	A	166	22.089	42.309	1.556	1.00	0.79	C
ATOM	303	O	THR	A	166	22.485	43.346	1.024	1.00	0.79	O
ATOM	304	CB	THR	A	166	21.899	40.753	−0.399	1.00	0.79	C
ATOM	305	OG1	THR	A	166	20.482	40.950	−0.362	1.00	0.79	O
ATOM	306	CG2	THR	A	166	22.261	39.395	−1.017	1.00	0.79	C
ATOM	307	N	GLY	A	167	21.199	42.282	2.554	1.00	0.77	N
ATOM	308	CA	GLY	A	167	20.635	43.504	3.152	1.00	0.77	C
ATOM	309	C	GLY	A	167	19.758	44.276	2.154	1.00	0.77	C
ATOM	310	O	GLY	A	167	19.547	45.474	2.329	1.00	0.77	O
ATOM	311	N	ARG	A	168	19.207	43.558	1.172	1.00	0.64	N
ATOM	312	CA	ARG	A	168	18.267	44.109	0.177	1.00	0.64	C
ATOM	313	C	ARG	A	168	17.013	43.230	0.053	1.00	0.64	C

ATOM	314	O	ARG	A	168	17.098	42.027	−0.199	1.00	0.64	O
ATOM	315	CB	ARG	A	168	18.961	44.226	−1.181	1.00	0.64	C
ATOM	316	CG	ARG	A	168	18.084	44.952	−2.206	1.00	0.64	C
ATOM	317	CD	ARG	A	168	17.862	46.417	−1.816	1.00	0.64	C
ATOM	318	NE	ARG	A	168	16.984	47.062	−2.808	1.00	0.64	N
ATOM	319	CZ	ARG	A	168	15.650	47.022	−2.829	1.00	0.64	C
ATOM	320	NH1	ARG	A	168	14.976	46.383	−1.891	1.00	0.64	N
ATOM	321	NH2	ARG	A	168	14.971	47.634	−3.788	1.00	0.64	N
ATOM	322	N	LEU	A	169	15.868	43.838	0.366	1.00	0.65	N
ATOM	323	CA	LEU	A	169	14.582	43.119	0.374	1.00	0.65	C
ATOM	324	C	LEU	A	169	13.979	43.112	−1.026	1.00	0.65	C
ATOM	325	O	LEU	A	169	13.479	44.117	−1.518	1.00	0.65	O
ATOM	326	CB	LEU	A	169	13.601	43.721	1.392	1.00	0.65	C
ATOM	327	CG	LEU	A	169	12.299	42.915	1.508	1.00	0.65	C
ATOM	328	CD1	LEU	A	169	12.546	41.484	2.001	1.00	0.65	C
ATOM	329	CD2	LEU	A	169	11.320	43.638	2.432	1.00	0.65	C
ATOM	330	N	VAL	A	170	13.998	41.938	−1.633	1.00	0.67	N
ATOM	331	CA	VAL	A	170	13.499	41.782	−3.010	1.00	0.67	C
ATOM	332	C	VAL	A	170	12.285	40.847	−2.986	1.00	0.67	C
ATOM	333	O	VAL	A	170	12.345	39.742	−2.444	1.00	0.67	O
ATOM	334	CB	VAL	A	170	14.608	41.229	−3.928	1.00	0.67	C
ATOM	335	CG1	VAL	A	170	14.140	41.133	−5.385	1.00	0.67	C
ATOM	336	CG2	VAL	A	170	15.885	42.077	−3.876	1.00	0.67	C
ATOM	337	N	SER	A	171	11.202	41.315	−3.596	1.00	0.73	N
ATOM	338	CA	SER	A	171	10.010	40.477	−3.817	1.00	0.73	C
ATOM	339	C	SER	A	171	10.270	39.534	−4.998	1.00	0.73	C
ATOM	340	O	SER	A	171	10.656	39.963	−6.085	1.00	0.73	O
ATOM	341	CB	SER	A	171	8.789	41.357	−4.083	1.00	0.73	C
ATOM	342	OG	SER	A	171	7.624	40.541	−4.217	1.00	0.73	O
ATOM	343	N	LEU	A	172	10.001	38.258	−4.771	1.00	0.71	N
ATOM	344	CA	LEU	A	172	10.288	37.194	−5.746	1.00	0.71	C
ATOM	345	C	LEU	A	172	9.016	36.580	−6.342	1.00	0.71	C
ATOM	346	O	LEU	A	172	7.916	36.750	−5.819	1.00	0.71	O
ATOM	347	CB	LEU	A	172	11.188	36.149	−5.076	1.00	0.71	C
ATOM	348	CG	LEU	A	172	12.518	36.746	−4.600	1.00	0.71	C
ATOM	349	CD1	LEU	A	172	13.323	35.677	−3.872	1.00	0.71	C
ATOM	350	CD2	LEU	A	172	13.348	37.319	−5.754	1.00	0.71	C
ATOM	351	N	SER	A	173	9.212	35.884	−7.453	1.00	0.79	N
ATOM	352	CA	SER	A	173	8.128	35.341	−8.292	1.00	0.79	C
ATOM	353	C	SER	A	173	7.606	33.965	−7.850	1.00	0.79	C
ATOM	354	O	SER	A	173	8.185	32.917	−8.147	1.00	0.79	O
ATOM	355	CB	SER	A	173	8.598	35.284	−9.747	1.00	0.79	C

ATOM	356	OG	SER	A	173	7.627	34.610	−10.554	1.00	0.79	O
ATOM	357	N	GLU	A	174	6.429	33.987	−7.233	1.00	0.80	N
ATOM	358	CA	GLU	A	174	5.654	32.749	−6.970	1.00	0.80	C
ATOM	359	C	GLU	A	174	5.159	32.130	−8.281	1.00	0.80	C
ATOM	360	O	GLU	A	174	5.145	30.910	−8.450	1.00	0.80	O
ATOM	361	CB	GLU	A	174	4.410	32.960	−6.101	1.00	0.80	C
ATOM	362	CG	GLU	A	174	4.729	33.422	−4.679	1.00	0.80	C
ATOM	363	CD	GLU	A	174	5.019	34.926	−4.525	1.00	0.80	C
ATOM	364	OE1	GLU	A	174	5.039	35.639	−5.552	1.00	0.80	O
ATOM	365	OE2	GLU	A	174	5.140	35.363	−3.365	1.00	0.80	O
ATOM	366	N	GLN	A	175	4.818	33.009	−9.221	1.00	0.78	N
ATOM	367	CA	GLN	A	175	4.307	32.606	−10.534	1.00	0.78	C
ATOM	368	C	GLN	A	175	5.287	31.736	−11.334	1.00	0.78	C
ATOM	369	O	GLN	A	175	4.867	30.798	−12.016	1.00	0.78	O
ATOM	370	CB	GLN	A	175	3.961	33.847	−11.347	1.00	0.78	C
ATOM	371	CG	GLN	A	175	3.150	33.378	−12.544	1.00	0.78	C
ATOM	372	CD	GLN	A	175	1.823	32.758	−12.084	1.00	0.78	C
ATOM	373	OE1	GLN	A	175	1.008	33.334	−11.370	1.00	0.78	O
ATOM	374	NE2	GLN	A	175	1.512	31.616	−12.654	1.00	0.78	N
ATOM	375	N	GLU	A	176	6.576	32.035	−11.193	1.00	0.78	N
ATOM	376	CA	GLU	A	176	7.625	31.262	−11.880	1.00	0.78	C
ATOM	377	C	GLU	A	176	7.588	29.805	−11.404	1.00	0.78	C
ATOM	378	O	GLU	A	176	7.580	28.893	−12.221	1.00	0.78	O
ATOM	379	CB	GLU	A	176	9.011	31.839	−11.602	1.00	0.78	C
ATOM	380	CG	GLU	A	176	10.075	31.124	−12.445	1.00	0.78	C
ATOM	381	CD	GLU	A	176	11.501	31.570	−12.118	1.00	0.78	C
ATOM	382	OE1	GLU	A	176	11.665	32.721	−11.649	1.00	0.78	O
ATOM	383	OE2	GLU	A	176	12.389	30.706	−12.259	1.00	0.78	O
ATOM	384	N	LEU	A	177	7.439	29.624	−10.095	1.00	0.82	N
ATOM	385	CA	LEU	A	177	7.284	28.286	−9.498	1.00	0.82	C
ATOM	386	C	LEU	A	177	6.052	27.557	−10.035	1.00	0.82	C
ATOM	387	O	LEU	A	177	6.189	26.471	−10.591	1.00	0.82	O
ATOM	388	CB	LEU	A	177	7.191	28.370	−7.975	1.00	0.82	C
ATOM	389	CG	LEU	A	177	8.490	28.854	−7.328	1.00	0.82	C
ATOM	390	CD1	LEU	A	177	8.286	28.963	−5.817	1.00	0.82	C
ATOM	391	CD2	LEU	A	177	9.655	27.905	−7.628	1.00	0.82	C
ATOM	392	N	VAL	A	178	4.923	28.262	−10.043	1.00	0.82	N
ATOM	393	CA	VAL	A	178	3.652	27.725	−10.574	1.00	0.82	C
ATOM	394	C	VAL	A	178	3.821	27.244	−12.026	1.00	0.82	C
ATOM	395	O	VAL	A	178	3.445	26.121	−12.349	1.00	0.82	O
ATOM	396	CB	VAL	A	178	2.526	28.774	−10.461	1.00	0.82	C
ATOM	397	CG1	VAL	A	178	1.213	28.323	−11.116	1.00	0.82	C

ATOM	398	CG2	VAL	A	178	2.239	29.094	−8.992	1.00	0.82	C
ATOM	399	N	ASP	A	179	4.375	28.117	−12.862	1.00	0.80	N
ATOM	400	CA	ASP	A	179	4.501	27.839	−14.304	1.00	0.80	C
ATOM	401	C	ASP	A	179	5.628	26.872	−14.680	1.00	0.80	C
ATOM	402	O	ASP	A	179	5.442	26.013	−15.537	1.00	0.80	O
ATOM	403	CB	ASP	A	179	4.673	29.136	−15.094	1.00	0.80	C
ATOM	404	CG	ASP	A	179	3.469	30.077	−14.995	1.00	0.80	C
ATOM	405	OD1	ASP	A	179	2.404	29.654	−14.491	1.00	0.80	O
ATOM	406	OD2	ASP	A	179	3.628	31.256	−15.375	1.00	0.80	O
ATOM	407	N	CYS	A	180	6.769	27.028	−14.020	1.00	0.83	N
ATOM	408	CA	CYS	A	180	8.017	26.366	−14.438	1.00	0.83	C
ATOM	409	C	CYS	A	180	8.425	25.152	−13.597	1.00	0.83	C
ATOM	410	O	CYS	A	180	8.994	24.201	−14.132	1.00	0.83	O
ATOM	411	CB	CYS	A	180	9.150	27.393	−14.459	1.00	0.83	C
ATOM	412	SG	CYS	A	180	8.888	28.758	−15.649	1.00	0.83	S
ATOM	413	N	ASP	A	181	8.167	25.195	−12.292	1.00	0.80	N
ATOM	414	CA	ASP	A	181	8.476	24.058	−11.409	1.00	0.80	C
ATOM	415	C	ASP	A	181	7.411	22.953	−11.551	1.00	0.80	C
ATOM	416	O	ASP	A	181	6.536	22.807	−10.700	1.00	0.80	O
ATOM	417	CB	ASP	A	181	8.618	24.570	−9.969	1.00	0.80	C
ATOM	418	CG	ASP	A	181	9.039	23.475	−8.988	1.00	0.80	C
ATOM	419	OD1	ASP	A	181	9.582	22.453	−9.462	1.00	0.80	O
ATOM	420	OD2	ASP	A	181	8.875	23.738	−7.780	1.00	0.80	O
ATOM	421	N	VAL	A	182	7.601	22.118	−12.570	1.00	0.73	N
ATOM	422	CA	VAL	A	182	6.614	21.079	−12.963	1.00	0.73	C
ATOM	423	C	VAL	A	182	7.114	19.622	−12.827	1.00	0.73	C
ATOM	424	O	VAL	A	182	6.410	18.665	−13.146	1.00	0.73	O
ATOM	425	CB	VAL	A	182	6.103	21.394	−14.389	1.00	0.73	C
ATOM	426	CG1	VAL	A	182	7.219	21.328	−15.439	1.00	0.73	C
ATOM	427	CG2	VAL	A	182	4.899	20.542	−14.817	1.00	0.73	C
ATOM	428	N	SER	A	183	8.359	19.444	−12.406	1.00	0.67	N
ATOM	429	CA	SER	A	183	8.921	18.084	−12.331	1.00	0.67	C
ATOM	430	C	SER	A	183	9.145	17.646	−10.886	1.00	0.67	C
ATOM	431	O	SER	A	183	9.117	18.458	−9.959	1.00	0.67	O
ATOM	432	CB	SER	A	183	10.217	17.970	−13.135	1.00	0.67	C
ATOM	433	OG	SER	A	183	10.619	16.596	−13.154	1.00	0.67	O
ATOM	434	N	GLY	A	184	9.209	16.319	−10.731	1.00	0.67	N
ATOM	435	CA	GLY	A	184	9.382	15.658	−9.426	1.00	0.67	C
ATOM	436	C	GLY	A	184	8.067	15.650	−8.638	1.00	0.67	C
ATOM	437	O	GLY	A	184	7.706	14.631	−8.058	1.00	0.67	O
ATOM	438	N	VAL	A	185	7.362	16.780	−8.666	1.00	0.68	N
ATOM	439	CA	VAL	A	185	6.057	16.927	−7.994	1.00	0.68	C

ATOM	440	C	VAL	A	185	5.140	17.999	−8.598	1.00	0.68	C
ATOM	441	O	VAL	A	185	4.105	17.674	−9.175	1.00	0.68	O
ATOM	442	CB	VAL	A	185	6.247	17.086	−6.464	1.00	0.68	C
ATOM	443	CG1	VAL	A	185	7.024	18.321	−6.078	1.00	0.68	C
ATOM	444	CG2	VAL	A	185	4.939	17.176	−5.681	1.00	0.68	C
ATOM	445	N	ASP	A	186	5.544	19.252	−8.389	1.00	0.76	N
ATOM	446	CA	ASP	A	186	4.646	20.396	−8.225	1.00	0.76	C
ATOM	447	C	ASP	A	186	3.746	20.538	−9.433	1.00	0.76	C
ATOM	448	O	ASP	A	186	4.167	20.325	−10.568	1.00	0.76	O
ATOM	449	CB	ASP	A	186	5.459	21.675	−8.008	1.00	0.76	C
ATOM	450	CG	ASP	A	186	6.290	21.594	−6.725	1.00	0.76	C
ATOM	451	OD1	ASP	A	186	5.753	21.105	−5.714	1.00	0.76	O
ATOM	452	OD2	ASP	A	186	7.501	21.836	−6.794	1.00	0.76	O
ATOM	453	N	GLN	A	187	2.492	20.798	−9.127	1.00	0.74	N
ATOM	454	CA	GLN	A	187	1.554	20.917	−10.244	1.00	0.74	C
ATOM	455	C	GLN	A	187	0.789	22.264	−10.222	1.00	0.74	C
ATOM	456	O	GLN	A	187	−0.407	22.414	−10.468	1.00	0.74	O
ATOM	457	CB	GLN	A	187	0.736	19.623	−10.430	1.00	0.74	C
ATOM	458	CG	GLN	A	187	1.010	18.210	−9.898	1.00	0.74	C
ATOM	459	CD	GLN	A	187	−0.280	17.377	−9.718	1.00	0.74	C
ATOM	460	OE1	GLN	A	187	−1.382	17.606	−10.203	1.00	0.74	O
ATOM	461	NE2	GLN	A	187	−0.107	16.344	−8.933	1.00	0.74	N
ATOM	462	N	GLY	A	188	1.567	23.316	−9.933	1.00	0.84	N
ATOM	463	CA	GLY	A	188	1.093	24.715	−10.005	1.00	0.84	C
ATOM	464	C	GLY	A	188	−0.109	24.966	−9.086	1.00	0.84	C
ATOM	465	O	GLY	A	188	−0.058	24.677	−7.890	1.00	0.84	O
ATOM	466	N	CYS	A	189	−1.209	25.395	−9.698	1.00	0.83	N
ATOM	467	CA	CYS	A	189	−2.479	25.659	−8.988	1.00	0.83	C
ATOM	468	C	CYS	A	189	−3.178	24.420	−8.410	1.00	0.83	C
ATOM	469	O	CYS	A	189	−4.204	24.554	−7.745	1.00	0.83	O
ATOM	470	CB	CYS	A	189	−3.478	26.385	−9.888	1.00	0.83	C
ATOM	471	SG	CYS	A	189	−2.865	27.977	−10.540	1.00	0.83	S
ATOM	472	N	ASP	A	190	−2.622	23.232	−8.616	1.00	0.84	N
ATOM	473	CA	ASP	A	190	−3.255	22.023	−8.043	1.00	0.84	C
ATOM	474	C	ASP	A	190	−2.455	21.433	−6.852	1.00	0.84	C
ATOM	475	O	ASP	A	190	−2.504	20.226	−6.601	1.00	0.84	O
ATOM	476	CB	ASP	A	190	−3.506	21.023	−9.193	1.00	0.84	C
ATOM	477	CG	ASP	A	190	−2.190	20.486	−9.757	1.00	0.84	C
ATOM	478	OD1	ASP	A	190	−1.254	20.541	−8.930	1.00	0.84	O
ATOM	479	OD2	ASP	A	190	−2.109	20.218	−10.970	1.00	0.84	O
ATOM	480	N	GLY	A	191	−1.518	22.238	−6.317	1.00	0.87	N
ATOM	481	CA	GLY	A	191	−0.679	21.818	−5.183	1.00	0.87	C

ATOM	482	C	GLY	A	191	0.775	21.509	−5.563	1.00	0.87	C
ATOM	483	O	GLY	A	191	1.152	21.361	−6.727	1.00	0.87	O
ATOM	484	N	GLY	A	192	1.584	21.482	−4.504	1.00	0.85	N
ATOM	485	CA	GLY	A	192	3.019	21.177	−4.590	1.00	0.85	C
ATOM	486	C	GLY	A	192	3.622	21.077	−3.186	1.00	0.85	C
ATOM	487	O	GLY	A	192	2.905	21.079	−2.186	1.00	0.85	O
ATOM	488	N	LEU	A	193	4.945	21.038	−3.145	1.00	0.84	N
ATOM	489	CA	LEU	A	193	5.713	20.909	−1.900	1.00	0.84	C
ATOM	490	C	LEU	A	193	6.867	21.917	−1.916	1.00	0.84	C
ATOM	491	O	LEU	A	193	7.579	22.035	−2.914	1.00	0.84	O
ATOM	492	CB	LEU	A	193	6.228	19.466	−1.814	1.00	0.84	C
ATOM	493	CG	LEU	A	193	6.780	19.098	−0.433	1.00	0.84	C
ATOM	494	CD1	LEU	A	193	5.690	19.160	0.643	1.00	0.84	C
ATOM	495	CD2	LEU	A	193	7.395	17.698	−0.482	1.00	0.84	C
ATOM	496	N	MET	A	194	7.131	22.511	−0.755	1.00	0.84	N
ATOM	497	CA	MET	A	194	8.199	23.530	−0.631	1.00	0.84	C
ATOM	498	C	MET	A	194	9.598	22.977	−0.907	1.00	0.84	C
ATOM	499	O	MET	A	194	10.367	23.581	−1.650	1.00	0.84	O
ATOM	500	CB	MET	A	194	8.232	24.216	0.736	1.00	0.84	C
ATOM	501	CG	MET	A	194	6.997	25.082	0.952	1.00	0.84	C
ATOM	502	SD	MET	A	194	5.505	24.080	1.278	1.00	0.84	S
ATOM	503	CE	MET	A	194	5.729	23.719	3.007	1.00	0.84	C
ATOM	504	N	ASP	A	195	9.859	21.763	−0.421	1.00	0.85	N
ATOM	505	CA	ASP	A	195	11.161	21.105	−0.645	1.00	0.85	C
ATOM	506	C	ASP	A	195	11.525	20.945	−2.120	1.00	0.85	C
ATOM	507	O	ASP	A	195	12.645	21.262	−2.505	1.00	0.85	O
ATOM	508	CB	ASP	A	195	11.258	19.743	0.045	1.00	0.85	C
ATOM	509	CG	ASP	A	195	11.468	19.877	1.554	1.00	0.85	C
ATOM	510	OD1	ASP	A	195	11.973	20.942	1.979	1.00	0.85	O
ATOM	511	OD2	ASP	A	195	11.104	18.910	2.250	1.00	0.85	O
ATOM	512	N	ASN	A	196	10.544	20.580	−2.936	1.00	0.81	N
ATOM	513	CA	ASN	A	196	10.801	20.462	−4.380	1.00	0.81	C
ATOM	514	C	ASN	A	196	11.010	21.820	−5.059	1.00	0.81	C
ATOM	515	O	ASN	A	196	11.830	21.939	−5.966	1.00	0.81	O
ATOM	516	CB	ASN	A	196	9.626	19.784	−5.031	1.00	0.81	C
ATOM	517	CG	ASN	A	196	9.888	19.482	−6.508	1.00	0.81	C
ATOM	518	OD1	ASN	A	196	9.648	20.283	−7.396	1.00	0.81	O
ATOM	519	ND2	ASN	A	196	10.229	18.244	−6.783	1.00	0.81	N
ATOM	520	N	ALA	A	197	10.333	22.834	−4.524	1.00	0.86	N
ATOM	521	CA	ALA	A	197	10.454	24.221	−5.007	1.00	0.86	C
ATOM	522	C	ALA	A	197	11.846	24.803	−4.721	1.00	0.86	C
ATOM	523	O	ALA	A	197	12.437	25.455	−5.579	1.00	0.86	O

ATOM	524	CB	ALA	A	197	9.372	25.086	−4.361	1.00	0.86	C
ATOM	525	N	PHE	A	198	12.403	24.459	−3.561	1.00	0.84	N
ATOM	526	CA	PHE	A	198	13.773	24.853	−3.187	1.00	0.84	C
ATOM	527	C	PHE	A	198	14.825	24.174	−4.073	1.00	0.84	C
ATOM	528	O	PHE	A	198	15.777	24.788	−4.543	1.00	0.84	O
ATOM	529	CB	PHE	A	198	14.065	24.487	−1.739	1.00	0.84	C
ATOM	530	CG	PHE	A	198	13.328	25.329	−0.703	1.00	0.84	C
ATOM	531	CD1	PHE	A	198	13.338	26.717	−0.778	1.00	0.84	C
ATOM	532	CD2	PHE	A	198	12.720	24.684	0.364	1.00	0.84	C
ATOM	533	CE1	PHE	A	198	12.740	27.463	0.227	1.00	0.84	C
ATOM	534	CE2	PHE	A	198	12.123	25.432	1.369	1.00	0.84	C
ATOM	535	CZ	PHE	A	198	12.136	26.820	1.301	1.00	0.84	C
ATOM	536	N	GLN	A	199	14.558	22.914	−4.390	1.00	0.79	N
ATOM	537	CA	GLN	A	199	15.400	22.172	−5.347	1.00	0.79	C
ATOM	538	C	GLN	A	199	15.367	22.872	−6.714	1.00	0.79	C
ATOM	539	O	GLN	A	199	16.405	23.006	−7.356	1.00	0.79	O
ATOM	540	CB	GLN	A	199	14.916	20.733	−5.512	1.00	0.79	C
ATOM	541	CG	GLN	A	199	14.965	20.017	−4.172	1.00	0.79	C
ATOM	542	CD	GLN	A	199	14.555	18.556	−4.212	1.00	0.79	C
ATOM	543	OE1	GLN	A	199	15.074	17.748	−4.965	1.00	0.79	O
ATOM	544	NE2	GLN	A	199	13.634	18.194	−3.346	1.00	0.79	N
ATOM	545	N	PHE	A	200	14.191	23.420	−7.033	1.00	0.80	N
ATOM	546	CA	PHE	A	200	13.945	24.136	−8.312	1.00	0.80	C
ATOM	547	C	PHE	A	200	14.807	25.348	−8.490	1.00	0.80	C
ATOM	548	O	PHE	A	200	15.578	25.504	−9.431	1.00	0.80	O
ATOM	549	CB	PHE	A	200	12.458	24.449	−8.533	1.00	0.80	C
ATOM	550	CG	PHE	A	200	12.244	25.203	−9.855	1.00	0.80	C
ATOM	551	CD1	PHE	A	200	12.080	24.660	−11.136	1.00	0.80	C
ATOM	552	CD2	PHE	A	200	12.181	26.572	−9.712	1.00	0.80	C
ATOM	553	CE1	PHE	A	200	11.828	25.454	−12.232	1.00	0.80	C
ATOM	554	CE2	PHE	A	200	11.939	27.378	−10.811	1.00	0.80	C
ATOM	555	CZ	PHE	A	200	11.755	26.823	−12.069	1.00	0.80	C
ATOM	556	N	VAL	A	201	14.699	26.163	−7.464	1.00	0.82	N
ATOM	557	CA	VAL	A	201	15.447	27.412	−7.411	1.00	0.82	C
ATOM	558	C	VAL	A	201	16.961	27.145	−7.375	1.00	0.82	C
ATOM	559	O	VAL	A	201	17.702	27.815	−8.090	1.00	0.82	O
ATOM	560	CB	VAL	A	201	14.905	28.276	−6.262	1.00	0.82	C
ATOM	561	CG1	VAL	A	201	14.928	27.562	−4.925	1.00	0.82	C
ATOM	562	CG2	VAL	A	201	15.659	29.593	−6.114	1.00	0.82	C
ATOM	563	N	ALA	A	202	17.372	26.098	−6.659	1.00	0.84	N
ATOM	564	CA	ALA	A	202	18.791	25.711	−6.566	1.00	0.84	C
ATOM	565	C	ALA	A	202	19.349	25.296	−7.936	1.00	0.84	C

ATOM	566	O	ALA	A	202	20.328	25.873	−8.405	1.00	0.84	O
ATOM	567	CB	ALA	A	202	18.956	24.568	−5.562	1.00	0.84	C
ATOM	568	N	ARG	A	203	18.580	24.464	−8.637	1.00	0.73	N
ATOM	569	CA	ARG	A	203	18.957	23.950	−9.968	1.00	0.73	C
ATOM	570	C	ARG	A	203	18.890	24.994	−11.092	1.00	0.73	C
ATOM	571	O	ARG	A	203	19.669	24.931	−12.040	1.00	0.73	O
ATOM	572	CB	ARG	A	203	18.140	22.704	−10.336	1.00	0.73	C
ATOM	573	CG	ARG	A	203	16.638	22.961	−10.487	1.00	0.73	C
ATOM	574	CD	ARG	A	203	15.900	21.703	−10.944	1.00	0.73	C
ATOM	575	NE	ARG	A	203	14.440	21.899	−11.001	1.00	0.73	N
ATOM	576	CZ	ARG	A	203	13.588	21.722	−9.991	1.00	0.73	C
ATOM	577	NH1	ARG	A	203	14.014	21.396	−8.781	1.00	0.73	N
ATOM	578	NH2	ARG	A	203	12.298	21.943	−10.103	1.00	0.73	N
ATOM	579	N	ARG	A	204	17.954	25.934	−10.974	1.00	0.71	N
ATOM	580	CA	ARG	A	204	17.791	27.012	−11.969	1.00	0.71	C
ATOM	581	C	ARG	A	204	18.864	28.099	−11.854	1.00	0.71	C
ATOM	582	O	ARG	A	204	19.012	28.935	−12.744	1.00	0.71	O
ATOM	583	CB	ARG	A	204	16.393	27.638	−11.888	1.00	0.71	C
ATOM	584	CG	ARG	A	204	15.276	26.661	−12.276	1.00	0.71	C
ATOM	585	CD	ARG	A	204	15.523	25.895	−13.580	1.00	0.71	C
ATOM	586	NE	ARG	A	204	15.845	26.813	−14.688	1.00	0.71	N
ATOM	587	CZ	ARG	A	204	15.789	26.501	−15.981	1.00	0.71	C
ATOM	588	NH1	ARG	A	204	15.381	25.299	−16.367	1.00	0.71	N
ATOM	589	NH2	ARG	A	204	16.182	27.370	−16.902	1.00	0.71	N
ATOM	590	N	GLY	A	205	19.530	28.087	−10.693	1.00	0.76	N
ATOM	591	CA	GLY	A	205	20.526	29.103	−10.317	1.00	0.76	C
ATOM	592	C	GLY	A	205	19.840	30.364	−9.776	1.00	0.76	C
ATOM	593	O	GLY	A	205	20.426	31.445	−9.756	1.00	0.76	O
ATOM	594	N	GLY	A	206	18.621	30.158	−9.265	1.00	0.77	N
ATOM	595	CA	GLY	A	206	17.832	31.210	−8.624	1.00	0.77	C
ATOM	596	C	GLY	A	206	16.417	31.350	−9.186	1.00	0.77	C
ATOM	597	O	GLY	A	206	15.961	30.579	−10.027	1.00	0.77	O
ATOM	598	N	LEU	A	207	15.726	32.309	−8.593	1.00	0.74	N
ATOM	599	CA	LEU	A	207	14.340	32.657	−8.939	1.00	0.74	C
ATOM	600	C	LEU	A	207	14.296	34.156	−9.278	1.00	0.74	C
ATOM	601	O	LEU	A	207	15.001	34.964	−8.667	1.00	0.74	O
ATOM	602	CB	LEU	A	207	13.475	32.268	−7.730	1.00	0.74	C
ATOM	603	CG	LEU	A	207	11.965	32.354	−7.963	1.00	0.74	C
ATOM	604	CD1	LEU	A	207	11.213	31.483	−6.963	1.00	0.74	C
ATOM	605	CD2	LEU	A	207	11.499	33.756	−7.641	1.00	0.74	C
ATOM	606	N	ALA	A	208	13.490	34.486	−10.282	1.00	0.70	N
ATOM	607	CA	ALA	A	208	13.334	35.868	−10.776	1.00	0.70	C

ATOM	608	C	ALA	A	208	12.445	36.727	−9.859	1.00	0.70	C
ATOM	609	O	ALA	A	208	11.705	36.241	−9.010	1.00	0.70	O
ATOM	610	CB	ALA	A	208	12.776	35.836	−12.202	1.00	0.70	C
ATOM	611	N	SER	A	209	12.534	38.036	−10.027	1.00	0.69	N
ATOM	612	CA	SER	A	209	11.716	38.974	−9.231	1.00	0.69	C
ATOM	613	C	SER	A	209	10.221	38.883	−9.579	1.00	0.69	C
ATOM	614	O	SER	A	209	9.835	38.513	−10.690	1.00	0.69	O
ATOM	615	CB	SER	A	209	12.230	40.403	−9.422	1.00	0.69	C
ATOM	616	OG	SER	A	209	11.455	41.313	−8.636	1.00	0.69	O
ATOM	617	N	GLU	A	210	9.404	39.317	−8.622	1.00	0.69	N
ATOM	618	CA	GLU	A	210	7.946	39.472	−8.791	1.00	0.69	C
ATOM	619	C	GLU	A	210	7.625	40.412	−9.963	1.00	0.69	C
ATOM	620	O	GLU	A	210	6.767	40.103	−10.780	1.00	0.69	O
ATOM	621	CB	GLU	A	210	7.361	40.061	−7.503	1.00	0.69	C
ATOM	622	CG	GLU	A	210	5.841	40.275	−7.549	1.00	0.69	C
ATOM	623	CD	GLU	A	210	5.045	38.983	−7.772	1.00	0.69	C
ATOM	624	OE1	GLU	A	210	5.513	37.906	−7.342	1.00	0.69	O
ATOM	625	OE2	GLU	A	210	3.953	39.096	−8.373	1.00	0.69	O
ATOM	626	N	SER	A	211	8.357	41.519	−10.039	1.00	0.68	N
ATOM	627	CA	SER	A	211	8.189	42.526	−11.108	1.00	0.68	C
ATOM	628	C	SER	A	211	8.393	41.936	−12.512	1.00	0.68	C
ATOM	629	O	SER	A	211	7.630	42.234	−13.425	1.00	0.68	O
ATOM	630	CB	SER	A	211	9.175	43.682	−10.907	1.00	0.68	C
ATOM	631	OG	SER	A	211	10.523	43.193	−10.890	1.00	0.68	O
ATOM	632	N	GLY	A	212	9.385	41.038	−12.609	1.00	0.68	N
ATOM	633	CA	GLY	A	212	9.749	40.364	−13.869	1.00	0.68	C
ATOM	634	C	GLY	A	212	8.724	39.298	−14.273	1.00	0.68	C
ATOM	635	O	GLY	A	212	8.420	39.128	−15.452	1.00	0.68	O
ATOM	636	N	TYR	A	213	8.245	38.572	−13.267	1.00	0.71	N
ATOM	637	CA	TYR	A	213	7.304	37.461	−13.464	1.00	0.71	C
ATOM	638	C	TYR	A	213	6.174	37.561	−12.415	1.00	0.71	C
ATOM	639	O	TYR	A	213	6.151	36.810	−11.436	1.00	0.71	O
ATOM	640	CB	TYR	A	213	8.126	36.172	−13.366	1.00	0.71	C
ATOM	641	CG	TYR	A	213	7.547	34.973	−14.120	1.00	0.71	C
ATOM	642	CD1	TYR	A	213	6.187	34.808	−14.366	1.00	0.71	C
ATOM	643	CD2	TYR	A	213	8.446	34.022	−14.579	1.00	0.71	C
ATOM	644	CE1	TYR	A	213	5.738	33.700	−15.071	1.00	0.71	C
ATOM	645	CE2	TYR	A	213	8.004	32.916	−15.289	1.00	0.71	C
ATOM	646	CZ	TYR	A	213	6.648	32.757	−15.536	1.00	0.71	C
ATOM	647	OH	TYR	A	213	6.219	31.685	−16.251	1.00	0.71	O
ATOM	648	N	PRO	A	214	5.235	38.493	−12.627	1.00	0.76	N
ATOM	649	CA	PRO	A	214	4.170	38.805	−11.656	1.00	0.76	C

ATOM	650	C	PROA		214	3.144	37.680	−11.527	1.00 0.76	C
ATOM	651	O	PROA		214	2.902	36.946	−12.480	1.00 0.76	O
ATOM	652	CB	PROA		214	3.546	40.100	−12.174	1.00 0.76	C
ATOM	653	CG	PROA		214	3.741	40.006	−13.687	1.00 0.76	C
ATOM	654	CD	PROA		214	5.111	39.346	−13.826	1.00 0.76	C
ATOM	655	N	TYR	A	215	2.606	37.556	−10.321	1.00 0.75	N
ATOM	656	CA	TYR	A	215	1.592	36.551	−9.975	1.00 0.75	C
ATOM	657	C	TYR	A	215	0.234	36.948	−10.551	1.00 0.75	C
ATOM	658	O	TYR	A	215	−0.248	38.064	−10.362	1.00 0.75	O
ATOM	659	CB	TYR	A	215	1.492	36.442	−8.453	1.00 0.75	C
ATOM	660	CG	TYR	A	215	0.574	35.292	−8.047	1.00 0.75	C
ATOM	661	CD1	TYR	A	215	0.989	33.992	−8.304	1.00 0.75	C
ATOM	662	CD2	TYR	A	215	−0.630	35.554	−7.398	1.00 0.75	C
ATOM	663	CE1	TYR	A	215	0.198	32.936	−7.909	1.00 0.75	C
ATOM	664	CE2	TYR	A	215	−1.423	34.502	−6.988	1.00 0.75	C
ATOM	665	CZ	TYR	A	215	−0.989	33.221	−7.258	1.00 0.75	C
ATOM	666	OH	TYR	A	215	−1.807	32.206	−7.012	1.00 0.75	O
ATOM	667	N	GLN	A	216	−0.427	35.940	−11.079	1.00 0.70	N
ATOM	668	CA	GLN	A	216	−1.743	36.178	−11.690	1.00 0.70	C
ATOM	669	C	GLN	A	216	−2.776	35.092	−11.340	1.00 0.70	C
ATOM	670	O	GLN	A	216	−3.785	34.921	−12.021	1.00 0.70	O
ATOM	671	CB	GLN	A	216	−1.433	36.264	−13.170	1.00 0.70	C
ATOM	672	CG	GLN	A	216	−1.132	34.860	−13.709	1.00 0.70	C
ATOM	673	CD	GLN	A	216	0.306	34.541	−13.884	1.00 0.70	C
ATOM	674	OE1	GLN	A	216	1.174	35.240	−13.394	1.00 0.70	O
ATOM	675	NE2	GLN	A	216	0.558	33.393	−14.471	1.00 0.70	N
ATOM	676	N	CYSA		217	−2.380	34.271	−10.369	1.00 0.75	N
ATOM	677	CA	CYSA		217	−3.216	33.200	−9.801	1.00 0.75	C
ATOM	678	C	CYSA		217	−3.881	32.329	−10.886	1.00 0.75	C
ATOM	679	O	CYSA		217	−5.097	32.303	−11.055	1.00 0.75	O
ATOM	680	CB	CYSA		217	−4.244	33.860	−8.873	1.00 0.75	C
ATOM	681	SG	CYSA		217	−5.446	32.660	−8.205	1.00 0.75	S
ATOM	682	N	ARG	A	218	−3.040	31.616	−11.623	1.00 0.66	N
ATOM	683	CA	ARG	A	218	−3.486	30.718	−12.709	1.00 0.66	C
ATOM	684	C	ARG	A	218	−2.308	29.882	−13.217	1.00 0.66	C
ATOM	685	O	ARG	A	218	−1.145	30.235	−13.005	1.00 0.66	O
ATOM	686	CB	ARG	A	218	−4.140	31.487	−13.875	1.00 0.66	C
ATOM	687	CG	ARG	A	218	−3.176	32.405	−14.631	1.00 0.66	C
ATOM	688	CD	ARG	A	218	−3.861	33.122	−15.800	1.00 0.66	C
ATOM	689	NE	ARG	A	218	−2.935	34.017	−16.525	1.00 0.66	N
ATOM	690	CZ	ARG	A	218	−2.694	35.303	−16.246	1.00 0.66	C
ATOM	691	NH1	ARG	A	218	−3.333	35.887	−15.245	1.00 0.66	N

ATOM	692	NH2	ARG	A	218	−1.671	35.944	−16.809	1.00	0.66	N
ATOM	693	N	ASP	A	219	−2.647	28.733	−13.794	1.00	0.75	N
ATOM	694	CA	ASP	A	219	−1.649	27.836	−14.405	1.00	0.75	C
ATOM	695	C	ASP	A	219	−1.208	28.351	−15.779	1.00	0.75	C
ATOM	696	O	ASP	A	219	−1.860	28.113	−16.794	1.00	0.75	O
ATOM	697	CB	ASP	A	219	−2.164	26.399	−14.537	1.00	0.75	C
ATOM	698	CG	ASP	A	219	−2.382	25.742	−13.179	1.00	0.75	C
ATOM	699	OD1	ASP	A	219	−1.367	25.467	−12.502	1.00	0.75	O
ATOM	700	OD2	ASP	A	219	−3.567	25.598	−12.817	1.00	0.75	O
ATOM	701	N	GLY	A	220	−0.141	29.147	−15.737	1.00	0.78	N
ATOM	702	CA	GLY	A	220	0.473	29.702	−16.950	1.00	0.78	C
ATOM	703	C	GLY	A	220	1.550	28.753	−17.511	1.00	0.78	C
ATOM	704	O	GLY	A	220	2.127	27.966	−16.755	1.00	0.78	O
ATOM	705	N	PRO	A	221	1.815	28.834	−18.821	1.00	0.77	N
ATOM	706	CA	PRO	A	221	2.940	28.122	−19.453	1.00	0.77	C
ATOM	707	C	PRO	A	221	4.249	28.791	−19.013	1.00	0.77	C
ATOM	708	O	PRO	A	221	4.326	30.019	−18.945	1.00	0.77	O
ATOM	709	CB	PRO	A	221	2.689	28.294	−20.954	1.00	0.77	C
ATOM	710	CG	PRO	A	221	1.980	29.645	−21.053	1.00	0.77	C
ATOM	711	CD	PRO	A	221	1.087	29.654	−19.814	1.00	0.77	C
ATOM	712	N	CYS	A	222	5.238	27.977	−18.656	1.00	0.81	N
ATOM	713	CA	CYS	A	222	6.554	28.481	−18.215	1.00	0.81	C
ATOM	714	C	CYS	A	222	7.150	29.454	−19.243	1.00	0.81	C
ATOM	715	O	CYS	A	222	7.416	29.094	−20.390	1.00	0.81	O
ATOM	716	CB	CYS	A	222	7.517	27.313	−17.984	1.00	0.81	C
ATOM	717	SG	CYS	A	222	9.185	27.853	−17.453	1.00	0.81	S
ATOM	718	N	ARG	A	223	7.274	30.705	−18.813	1.00	0.66	N
ATOM	719	CA	ARG	A	223	7.853	31.764	−19.650	1.00	0.66	C
ATOM	720	C	ARG	A	223	9.374	31.699	−19.489	1.00	0.66	C
ATOM	721	O	ARG	A	223	9.952	32.316	−18.596	1.00	0.66	O
ATOM	722	CB	ARG	A	223	7.277	33.123	−19.236	1.00	0.66	C
ATOM	723	CG	ARG	A	223	7.769	34.246	−20.150	1.00	0.66	C
ATOM	724	CD	ARG	A	223	7.125	35.576	−19.759	1.00	0.66	C
ATOM	725	NE	ARG	A	223	7.633	36.651	−20.630	1.00	0.66	N
ATOM	726	CZ	ARG	A	223	8.785	37.311	−20.489	1.00	0.66	C
ATOM	727	NH1	ARG	A	223	9.621	37.035	−19.504	1.00	0.66	N
ATOM	728	NH2	ARG	A	223	9.110	38.289	−21.321	1.00	0.66	N
ATOM	729	N	SER	A	224	9.991	30.956	−20.406	1.00	0.69	N
ATOM	730	CA	SER	A	224	11.449	30.703	−20.390	1.00	0.69	C
ATOM	731	C	SER	A	224	12.290	31.990	−20.348	1.00	0.69	C
ATOM	732	O	SER	A	224	13.286	32.062	−19.634	1.00	0.69	O
ATOM	733	CB	SER	A	224	11.860	29.879	−21.613	1.00	0.69	C

ATOM	734	OG	SER	A	224	13.268	29.637	−21.582	1.00	0.69	O
ATOM	735	N	SER	A	225	11.796	33.033	−21.017	1.00	0.62	N
ATOM	736	CA	SER	A	225	12.483	34.342	−21.060	1.00	0.62	C
ATOM	737	C	SER	A	225	12.654	34.949	−19.657	1.00	0.62	C
ATOM	738	O	SER	A	225	13.735	35.415	−19.300	1.00	0.62	O
ATOM	739	CB	SER	A	225	11.715	35.325	−21.946	1.00	0.62	C
ATOM	740	OG	SER	A	225	12.458	36.541	−22.042	1.00	0.62	O
ATOM	741	N	ALA	A	226	11.621	34.777	−18.830	1.00	0.70	N
ATOM	742	CA	ALA	A	226	11.606	35.222	−17.423	1.00	0.70	C
ATOM	743	C	ALA	A	226	12.324	34.231	−16.486	1.00	0.70	C
ATOM	744	O	ALA	A	226	11.836	33.857	−15.416	1.00	0.70	O
ATOM	745	CB	ALA	A	226	10.158	35.450	−16.989	1.00	0.70	C
ATOM	746	N	ALA	A	227	13.503	33.811	−16.933	1.00	0.68	N
ATOM	747	CA	ALA	A	227	14.364	32.919	−16.145	1.00	0.68	C
ATOM	748	C	ALA	A	227	15.281	33.792	−15.300	1.00	0.68	C
ATOM	749	O	ALA	A	227	15.378	33.478	−14.125	1.00	0.68	O
ATOM	750	CB	ALA	A	227	15.186	31.968	−17.013	1.00	0.68	C
ATOM	751	N	ALA	A	228	15.918	34.777	−15.961	1.00	0.56	N
ATOM	752	CA	ALA	A	228	16.615	35.955	−15.385	1.00	0.56	C
ATOM	753	C	ALA	A	228	16.574	35.979	−13.852	1.00	0.56	C
ATOM	754	O	ALA	A	228	15.801	36.698	−13.213	1.00	0.56	O
ATOM	755	CB	ALA	A	228	15.977	37.220	−15.981	1.00	0.56	C
ATOM	756	N	ALA	A	229	17.323	35.026	−13.289	1.00	0.66	N
ATOM	757	CA	ALA	A	229	17.266	34.757	−11.852	1.00	0.66	C
ATOM	758	C	ALA	A	229	17.813	35.993	−11.148	1.00	0.66	C
ATOM	759	O	ALA	A	229	18.689	36.678	−11.674	1.00	0.66	O
ATOM	760	CB	ALA	A	229	18.103	33.519	−11.519	1.00	0.66	C
ATOM	761	N	ALA	A	230	17.164	36.330	−10.049	1.00	0.66	N
ATOM	762	CA	ALA	A	230	17.549	37.520	−9.274	1.00	0.66	C
ATOM	763	C	ALA	A	230	18.168	37.131	−7.928	1.00	0.66	C
ATOM	764	O	ALA	A	230	19.019	37.840	−7.399	1.00	0.66	O
ATOM	765	CB	ALA	A	230	16.323	38.416	−9.074	1.00	0.66	C
ATOM	766	N	ALA	A	231	17.695	36.011	−7.385	1.00	0.78	N
ATOM	767	CA	ALA	A	231	18.174	35.483	−6.099	1.00	0.78	C
ATOM	768	C	ALA	A	231	18.423	33.980	−6.216	1.00	0.78	C
ATOM	769	O	ALA	A	231	17.514	33.243	−6.594	1.00	0.78	O
ATOM	770	CB	ALA	A	231	17.121	35.754	−5.020	1.00	0.78	C
ATOM	771	N	SER	A	232	19.614	33.569	−5.804	1.00	0.76	N
ATOM	772	CA	SER	A	232	20.048	32.159	−5.852	1.00	0.76	C
ATOM	773	C	SER	A	232	20.173	31.574	−4.446	1.00	0.76	C
ATOM	774	O	SER	A	232	20.242	32.286	−3.463	1.00	0.76	O
ATOM	775	CB	SER	A	232	21.412	32.046	−6.537	1.00	0.76	C

ATOM	776	OG	SER	A	232	22.386	32.782	−5.789	1.00	0.76	O
ATOM	777	N	ILE	A	233	20.034	30.269	−4.341	1.00	0.72	N
ATOM	778	CA	ILE	A	233	20.232	29.565	−3.061	1.00	0.72	C
ATOM	779	C	ILE	A	233	21.166	28.379	−3.337	1.00	0.72	C
ATOM	780	O	ILE	A	233	21.172	27.851	−4.449	1.00	0.72	O
ATOM	781	CB	ILE	A	233	18.885	29.131	−2.449	1.00	0.72	C
ATOM	782	CG1	ILE	A	233	18.186	28.060	−3.293	1.00	0.72	C
ATOM	783	CG2	ILE	A	233	17.967	30.345	−2.208	1.00	0.72	C
ATOM	784	CD1	ILE	A	233	17.144	27.326	−2.448	1.00	0.72	C
ATOM	785	N	ARG	A	234	21.901	27.944	−2.322	1.00	0.66	N
ATOM	786	CA	ARG	A	234	22.830	26.802	−2.478	1.00	0.66	C
ATOM	787	C	ARG	A	234	22.146	25.423	−2.370	1.00	0.66	C
ATOM	788	O	ARG	A	234	22.680	24.418	−2.834	1.00	0.66	O
ATOM	789	CB	ARG	A	234	24.089	26.923	−1.596	1.00	0.66	C
ATOM	790	CG	ARG	A	234	23.784	27.134	−0.112	1.00	0.66	C
ATOM	791	CD	ARG	A	234	24.913	26.895	0.900	1.00	0.66	C
ATOM	792	NE	ARG	A	234	24.422	26.917	2.302	1.00	0.66	N
ATOM	793	CZ	ARG	A	234	24.063	27.990	3.023	1.00	0.66	C
ATOM	794	NH1	ARG	A	234	24.117	29.189	2.498	1.00	0.66	N
ATOM	795	NH2	ARG	A	234	23.616	27.866	4.276	1.00	0.66	N
ATOM	796	N	GLY	A	235	20.989	25.395	−1.695	1.00	0.80	N
ATOM	797	CA	GLY	A	235	20.162	24.181	−1.511	1.00	0.80	C
ATOM	798	C	GLY	A	235	19.068	24.449	−0.465	1.00	0.80	C
ATOM	799	O	GLY	A	235	18.684	25.598	−0.236	1.00	0.80	O
ATOM	800	N	HIS	A	236	18.807	23.429	0.346	1.00	0.76	N
ATOM	801	CA	HIS	A	236	17.771	23.479	1.395	1.00	0.76	C
ATOM	802	C	HIS	A	236	17.900	22.272	2.328	1.00	0.76	C
ATOM	803	O	HIS	A	236	18.384	21.213	1.933	1.00	0.76	O
ATOM	804	CB	HIS	A	236	16.372	23.503	0.762	1.00	0.76	C
ATOM	805	CG	HIS	A	236	16.091	22.267	−0.097	1.00	0.76	C
ATOM	806	ND1	HIS	A	236	15.467	21.172	0.324	1.00	0.76	N
ATOM	807	CD2	HIS	A	236	16.536	22.050	−1.331	1.00	0.76	C
ATOM	808	CE1	HIS	A	236	15.525	20.266	−0.641	1.00	0.76	C
ATOM	809	NE2	HIS	A	236	16.190	20.813	−1.648	1.00	0.76	N
ATOM	810	N	GLU	A	237	17.475	22.489	3.564	1.00	0.77	N
ATOM	811	CA	GLU	A	237	17.490	21.456	4.610	1.00	0.77	C
ATOM	812	C	GLU	A	237	16.164	21.475	5.381	1.00	0.77	C
ATOM	813	O	GLU	A	237	15.481	22.500	5.433	1.00	0.77	O
ATOM	814	CB	GLU	A	237	18.690	21.696	5.540	1.00	0.77	C
ATOM	815	CG	GLU	A	237	18.867	20.606	6.608	1.00	0.77	C
ATOM	816	CD	GLU	A	237	19.109	19.221	5.995	1.00	0.77	C
ATOM	817	OE1	GLU	A	237	18.116	18.623	5.517	1.00	0.77	O

ATOM	818	OE2	GLU	A	237	20.284	18.805	6.003	1.00	0.77	O
ATOM	819	N	ASP	A	238	15.833	20.330	5.967	1.00	0.79	N
ATOM	820	CA	ASP	A	238	14.631	20.191	6.803	1.00	0.79	C
ATOM	821	C	ASP	A	238	15.022	20.278	8.284	1.00	0.79	C
ATOM	822	O	ASP	A	238	16.067	19.784	8.710	1.00	0.79	O
ATOM	823	CB	ASP	A	238	13.969	18.841	6.480	1.00	0.79	C
ATOM	824	CG	ASP	A	238	12.581	18.619	7.100	1.00	0.79	C
ATOM	825	OD1	ASP	A	238	12.315	19.153	8.179	1.00	0.79	O
ATOM	826	OD2	ASP	A	238	11.783	17.885	6.485	1.00	0.79	O
ATOM	827	N	VAL	A	239	14.142	20.924	9.039	1.00	0.80	N
ATOM	828	CA	VAL	A	239	14.193	20.930	10.511	1.00	0.80	C
ATOM	829	C	VAL	A	239	13.797	19.523	11.024	1.00	0.80	C
ATOM	830	O	VAL	A	239	12.809	18.948	10.573	1.00	0.80	O
ATOM	831	CB	VAL	A	239	13.229	22.000	11.059	1.00	0.80	C
ATOM	832	CG1	VAL	A	239	13.245	22.102	12.587	1.00	0.80	C
ATOM	833	CG2	VAL	A	239	13.509	23.383	10.464	1.00	0.80	C
ATOM	834	N	PRO	A	240	14.491	19.007	12.043	1.00	0.77	N
ATOM	835	CA	PRO	A	240	14.131	17.728	12.688	1.00	0.77	C
ATOM	836	C	PRO	A	240	12.636	17.754	13.035	1.00	0.77	C
ATOM	837	O	PRO	A	240	12.130	18.752	13.553	1.00	0.77	O
ATOM	838	CB	PRO	A	240	14.981	17.709	13.956	1.00	0.77	C
ATOM	839	CG	PRO	A	240	16.242	18.463	13.540	1.00	0.77	C
ATOM	840	CD	PRO	A	240	15.721	19.571	12.629	1.00	0.77	C
ATOM	841	N	ARG	A	241	11.937	16.716	12.587	1.00	0.68	N
ATOM	842	CA	ARG	A	241	10.469	16.657	12.704	1.00	0.68	C
ATOM	843	C	ARG	A	241	10.048	16.636	14.178	1.00	0.68	C
ATOM	844	O	ARG	A	241	10.748	16.092	15.033	1.00	0.68	O
ATOM	845	CB	ARG	A	241	9.941	15.411	11.988	1.00	0.68	C
ATOM	846	CG	ARG	A	241	8.412	15.326	12.046	1.00	0.68	C
ATOM	847	CD	ARG	A	241	7.881	14.055	11.393	1.00	0.68	C
ATOM	848	NE	ARG	A	241	8.061	14.159	9.937	1.00	0.68	N
ATOM	849	CZ	ARG	A	241	7.250	14.779	9.078	1.00	0.68	C
ATOM	850	NH1	ARG	A	241	6.127	15.355	9.479	1.00	0.68	N
ATOM	851	NH2	ARG	A	241	7.577	14.867	7.800	1.00	0.68	N
ATOM	852	N	ASN	A	242	8.895	17.246	14.430	1.00	0.72	N
ATOM	853	CA	ASN	A	242	8.234	17.188	15.751	1.00	0.72	C
ATOM	854	C	ASN	A	242	9.131	17.665	16.897	1.00	0.72	C
ATOM	855	O	ASN	A	242	9.254	17.029	17.946	1.00	0.72	O
ATOM	856	CB	ASN	A	242	7.726	15.766	16.049	1.00	0.72	C
ATOM	857	CG	ASN	A	242	6.718	15.342	14.996	1.00	0.72	C
ATOM	858	OD1	ASN	A	242	6.099	16.180	14.395	1.00	0.72	O
ATOM	859	ND2	ASN	A	242	6.627	14.047	14.748	1.00	0.72	N

ATOM	860	N	ASN	A	243	9.827	18.761	16.630	1.00	0.75	N
ATOM	861	CA	ASN	A	243	10.780	19.320	17.595	1.00	0.75	C
ATOM	862	C	ASN	A	243	10.737	20.846	17.513	1.00	0.75	C
ATOM	863	O	ASN	A	243	11.527	21.487	16.816	1.00	0.75	O
ATOM	864	CB	ASN	A	243	12.182	18.767	17.305	1.00	0.75	C
ATOM	865	CG	ASN	A	243	13.185	19.176	18.385	1.00	0.75	C
ATOM	866	OD1	ASN	A	243	13.023	20.147	19.118	1.00	0.75	O
ATOM	867	ND2	ASN	A	243	14.269	18.441	18.461	1.00	0.75	N
ATOM	868	N	GLU	A	244	9.847	21.421	18.313	1.00	0.76	N
ATOM	869	CA	GLU	A	244	9.672	22.886	18.339	1.00	0.76	C
ATOM	870	C	GLU	A	244	10.907	23.654	18.829	1.00	0.76	C
ATOM	871	O	GLU	A	244	11.116	24.790	18.416	1.00	0.76	O
ATOM	872	CB	GLU	A	244	8.433	23.308	19.128	1.00	0.76	C
ATOM	873	CG	GLU	A	244	7.163	22.949	18.354	1.00	0.76	C
ATOM	874	CD	GLU	A	244	5.934	23.598	18.990	1.00	0.76	C
ATOM	875	OE1	GLU	A	244	5.731	24.804	18.728	1.00	0.76	O
ATOM	876	OE2	GLU	A	244	5.243	22.921	19.782	1.00	0.76	O
ATOM	877	N	ALA	A	245	11.735	23.000	19.645	1.00	0.85	N
ATOM	878	CA	ALA	A	245	13.027	23.561	20.091	1.00	0.85	C
ATOM	879	C	ALA	A	245	14.013	23.710	18.919	1.00	0.85	C
ATOM	880	O	ALA	A	245	14.587	24.778	18.714	1.00	0.85	O
ATOM	881	CB	ALA	A	245	13.637	22.667	21.173	1.00	0.85	C
ATOM	882	N	ALA	A	246	14.039	22.692	18.058	1.00	0.88	N
ATOM	883	CA	ALA	A	246	14.855	22.696	16.827	1.00	0.88	C
ATOM	884	C	ALA	A	246	14.317	23.721	15.822	1.00	0.88	C
ATOM	885	O	ALA	A	246	15.068	24.498	15.232	1.00	0.88	O
ATOM	886	CB	ALA	A	246	14.853	21.310	16.183	1.00	0.88	C
ATOM	887	N	LEUA	247	12.991	23.818	15.791	1.00	0.86	N	
ATOM	888	CA	LEUA	247	12.297	24.793	14.945	1.00	0.86	C	
ATOM	889	C	LEUA	247	12.555	26.237	15.392	1.00	0.86	C	
ATOM	890	O	LEUA	247	12.801	27.107	14.561	1.00	0.86	O	
ATOM	891	CB	LEUA	247	10.807	24.479	14.951	1.00	0.86	C	
ATOM	892	CG	LEUA	247	10.042	25.410	14.018	1.00	0.86	C	
ATOM	893	CD1	LEUA	247	10.488	25.314	12.557	1.00	0.86	C	
ATOM	894	CD2	LEUA	247	8.595	25.003	14.148	1.00	0.86	C	
ATOM	895	N	ALA	A	248	12.650	26.420	16.705	1.00	0.89	N
ATOM	896	CA	ALA	A	248	12.929	27.729	17.324	1.00	0.89	C
ATOM	897	C	ALA	A	248	14.330	28.229	16.959	1.00	0.89	C
ATOM	898	O	ALA	A	248	14.477	29.347	16.470	1.00	0.89	O
ATOM	899	CB	ALA	A	248	12.782	27.629	18.843	1.00	0.89	C
ATOM	900	N	ALA	A	249	15.283	27.297	16.986	1.00	0.87	N
ATOM	901	CA	ALA	A	249	16.683	27.574	16.615	1.00	0.87	C

ATOM	902	C	ALA	A	249	16.815	27.938	15.128	1.00	0.87	C
ATOM	903	O	ALA	A	249	17.525	28.877	14.772	1.00	0.87	O
ATOM	904	CB	ALA	A	249	17.546	26.351	16.933	1.00	0.87	C
ATOM	905	N	ALA	A	250	16.004	27.277	14.301	1.00	0.90	N
ATOM	906	CA	ALA	A	250	15.969	27.506	12.844	1.00	0.90	C
ATOM	907	C	ALA	A	250	15.366	28.874	12.485	1.00	0.90	C
ATOM	908	O	ALA	A	250	15.957	29.638	11.722	1.00	0.90	O
ATOM	909	CB	ALA	A	250	15.173	26.386	12.173	1.00	0.90	C
ATOM	910	N	VAL	A	251	14.277	29.218	13.176	1.00	0.90	N
ATOM	911	CA	VAL	A	251	13.557	30.495	12.976	1.00	0.90	C
ATOM	912	C	VAL	A	251	14.401	31.689	13.448	1.00	0.90	C
ATOM	913	O	VAL	A	251	14.337	32.764	12.852	1.00	0.90	O
ATOM	914	CB	VAL	A	251	12.179	30.482	13.670	1.00	0.90	C
ATOM	915	CG1	VAL	A	251	11.425	31.808	13.503	1.00	0.90	C
ATOM	916	CG2	VAL	A	251	11.283	29.390	13.084	1.00	0.90	C
ATOM	917	N	ALA	A	252	15.202	31.464	14.488	1.00	0.87	N
ATOM	918	CA	ALA	A	252	16.092	32.500	15.047	1.00	0.87	C
ATOM	919	C	ALA	A	252	17.077	33.024	13.993	1.00	0.87	C
ATOM	920	O	ALA	A	252	17.454	34.193	14.020	1.00	0.87	O
ATOM	921	CB	ALA	A	252	16.865	31.930	16.237	1.00	0.87	C
ATOM	922	N	HIS	A	253	17.399	32.157	13.035	1.00	0.81	N
ATOM	923	CA	HIS	A	253	18.327	32.478	11.942	1.00	0.81	C
ATOM	924	C	HIS	A	253	17.636	33.144	10.741	1.00	0.81	C
ATOM	925	O	HIS	A	253	18.250	33.958	10.056	1.00	0.81	O
ATOM	926	CB	HIS	A	253	19.034	31.190	11.507	1.00	0.81	C
ATOM	927	CG	HIS	A	253	20.026	31.453	10.372	1.00	0.81	C
ATOM	928	ND1	HIS	A	253	19.714	31.543	9.082	1.00	0.81	N
ATOM	929	CD2	HIS	A	253	21.335	31.649	10.493	1.00	0.81	C
ATOM	930	CE1	HIS	A	253	20.827	31.790	8.402	1.00	0.81	C
ATOM	931	NE2	HIS	A	253	21.830	31.842	9.275	1.00	0.81	N
ATOM	932	N	GLN	A	254	16.417	32.705	10.438	1.00	0.80	N
ATOM	933	CA	GLN	A	254	15.728	33.060	9.185	1.00	0.80	C
ATOM	934	C	GLN	A	254	14.259	32.605	9.227	1.00	0.80	C
ATOM	935	O	GLN	A	254	13.930	31.694	9.995	1.00	0.80	O
ATOM	936	CB	GLN	A	254	16.443	32.381	8.002	1.00	0.80	C
ATOM	937	CG	GLN	A	254	16.331	30.858	8.096	1.00	0.80	C
ATOM	938	CD	GLN	A	254	17.194	30.146	7.074	1.00	0.80	C
ATOM	939	OE1	GLN	A	254	17.042	30.264	5.870	1.00	0.80	O
ATOM	940	NE2	GLN	A	254	18.105	29.359	7.601	1.00	0.80	N
ATOM	941	N	PRO	A	255	13.413	33.191	8.369	1.00	0.91	N
ATOM	942	CA	PRO	A	255	12.043	32.697	8.140	1.00	0.91	C
ATOM	943	C	PRO	A	255	12.111	31.244	7.650	1.00	0.91	C

ATOM	944	O	PRO	A	255	12.991	30.878	6.871	1.00	0.91	O
ATOM	945	CB	PRO	A	255	11.496	33.597	7.032	1.00	0.91	C
ATOM	946	CG	PRO	A	255	12.240	34.913	7.245	1.00	0.91	C
ATOM	947	CD	PRO	A	255	13.640	34.462	7.654	1.00	0.91	C
ATOM	948	N	VAL	A	256	11.199	30.427	8.158	1.00	0.87	N
ATOM	949	CA	VAL	A	256	11.160	28.986	7.828	1.00	0.87	C
ATOM	950	C	VAL	A	256	9.809	28.641	7.186	1.00	0.87	C
ATOM	951	O	VAL	A	256	8.754	28.958	7.738	1.00	0.87	O
ATOM	952	CB	VAL	A	256	11.424	28.140	9.095	1.00	0.87	C
ATOM	953	CG1	VAL	A	256	11.356	26.631	8.830	1.00	0.87	C
ATOM	954	CG2	VAL	A	256	12.804	28.444	9.688	1.00	0.87	C
ATOM	955	N	SER	A	257	9.888	27.842	6.127	1.00	0.88	N
ATOM	956	CA	SER	A	257	8.698	27.285	5.455	1.00	0.88	C
ATOM	957	C	SER	A	257	8.169	26.097	6.254	1.00	0.88	C
ATOM	958	O	SER	A	257	8.930	25.229	6.684	1.00	0.88	O
ATOM	959	CB	SER	A	257	9.036	26.785	4.052	1.00	0.88	C
ATOM	960	OG	SER	A	257	9.443	27.889	3.245	1.00	0.88	O
ATOM	961	N	VAL	A	258	6.867	26.121	6.492	1.00	0.85	N
ATOM	962	CA	VAL	A	258	6.193	25.062	7.265	1.00	0.85	C
ATOM	963	C	VAL	A	258	4.862	24.673	6.597	1.00	0.85	C
ATOM	964	O	VAL	A	258	4.237	25.468	5.889	1.00	0.85	O
ATOM	965	CB	VAL	A	258	5.972	25.492	8.731	1.00	0.85	C
ATOM	966	CG1	VAL	A	258	7.259	25.864	9.477	1.00	0.85	C
ATOM	967	CG2	VAL	A	258	5.011	26.671	8.797	1.00	0.85	C
ATOM	968	N	ALA	A	259	4.405	23.482	6.949	1.00	0.90	N
ATOM	969	CA	ALA	A	259	3.092	22.973	6.525	1.00	0.90	C
ATOM	970	C	ALA	A	259	2.216	22.779	7.764	1.00	0.90	C
ATOM	971	O	ALA	A	259	2.698	22.395	8.830	1.00	0.90	O
ATOM	972	CB	ALA	A	259	3.272	21.641	5.798	1.00	0.90	C
ATOM	973	N	ILE	A	260	0.954	23.148	7.608	1.00	0.82	N
ATOM	974	CA	ILE	A	260	−0.040	23.068	8.691	1.00	0.82	C
ATOM	975	C	ILE	A	260	−1.359	22.483	8.172	1.00	0.82	C
ATOM	976	O	ILE	A	260	−1.591	22.362	6.964	1.00	0.82	O
ATOM	977	CB	ILE	A	260	−0.275	24.448	9.337	1.00	0.82	C
ATOM	978	CG1	ILE	A	260	−0.825	25.447	8.310	1.00	0.82	C
ATOM	979	CG2	ILE	A	260	0.997	24.948	10.038	1.00	0.82	C
ATOM	980	CD1	ILE	A	260	−1.509	26.644	8.972	1.00	0.82	C
ATOM	981	N	ASN	A	261	−2.228	22.202	9.127	1.00	0.84	N
ATOM	982	CA	ASN	A	261	−3.607	21.797	8.854	1.00	0.84	C
ATOM	983	C	ASN	A	261	−4.483	23.042	9.039	1.00	0.84	C
ATOM	984	O	ASN	A	261	−4.803	23.437	10.162	1.00	0.84	O
ATOM	985	CB	ASN	A	261	−3.998	20.693	9.838	1.00	0.84	C

ATOM	986	CG	ASN	A	261	−5.361	20.107	9.475	1.00	0.84	C
ATOM	987	OD1	ASN	A	261	−6.182	20.734	8.831	1.00	0.84	O
ATOM	988	ND2	ASN	A	261	−5.620	18.908	9.943	1.00	0.84	N
ATOM	989	N	GLY	A	262	−4.816	23.658	7.901	1.00	0.84	N
ATOM	990	CA	GLY	A	262	−5.630	24.888	7.892	1.00	0.84	C
ATOM	991	C	GLY	A	262	−7.017	24.638	7.290	1.00	0.84	C
ATOM	992	O	GLY	A	262	−7.690	25.569	6.859	1.00	0.84	O
ATOM	993	N	GLU	A	263	−7.465	23.385	7.385	1.00	0.72	N
ATOM	994	CA	GLU	A	263	−8.776	22.977	6.841	1.00	0.72	C
ATOM	995	C	GLU	A	263	−9.953	23.237	7.789	1.00	0.72	C
ATOM	996	O	GLU	A	263	−11.087	22.906	7.467	1.00	0.72	O
ATOM	997	CB	GLU	A	263	−8.801	21.507	6.414	1.00	0.72	C
ATOM	998	CG	GLU	A	263	−8.689	20.515	7.578	1.00	0.72	C
ATOM	999	CD	GLU	A	263	−8.507	19.063	7.132	1.00	0.72	C
ATOM	1000	OE1	GLU	A	263	−9.089	18.718	6.085	1.00	0.72	O
ATOM	1001	OE2	GLU	A	263	−7.822	18.318	7.873	1.00	0.72	O
ATOM	1002	N	ASP	A	264	−9.646	23.581	9.032	1.00	0.73	N
ATOM	1003	CA	ASP	A	264	−10.712	23.799	10.022	1.00	0.73	C
ATOM	1004	C	ASP	A	264	−11.210	25.251	10.057	1.00	0.73	C
ATOM	1005	O	ASP	A	264	−10.489	26.191	9.723	1.00	0.73	O
ATOM	1006	CB	ASP	A	264	−10.250	23.355	11.406	1.00	0.73	C
ATOM	1007	CG	ASP	A	264	−9.066	24.171	11.890	1.00	0.73	C
ATOM	1008	OD1	ASP	A	264	−8.944	25.366	11.579	1.00	0.73	O
ATOM	1009	OD2	ASP	A	264	−8.251	23.527	12.511	1.00	0.73	O
ATOM	1010	N	MET	A	265	−12.370	25.385	10.682	1.00	0.69	N
ATOM	1011	CA	MET	A	265	−13.035	26.685	10.884	1.00	0.69	C
ATOM	1012	C	MET	A	265	−12.325	27.653	11.844	1.00	0.69	C
ATOM	1013	O	MET	A	265	−12.183	28.844	11.590	1.00	0.69	O
ATOM	1014	CB	MET	A	265	−14.496	26.497	11.286	1.00	0.69	C
ATOM	1015	CG	MET	A	265	−14.609	25.792	12.640	1.00	0.69	C
ATOM	1016	SD	MET	A	265	−16.327	25.555	13.201	1.00	0.69	S
ATOM	1017	CE	MET	A	265	−16.843	24.348	11.999	1.00	0.69	C
ATOM	1018	N	ALA	A	266	−11.800	27.094	12.926	1.00	0.78	N
ATOM	1019	CA	ALA	A	266	−11.118	27.897	13.954	1.00	0.78	C
ATOM	1020	C	ALA	A	266	−9.918	28.661	13.379	1.00	0.78	C
ATOM	1021	O	ALA	A	266	−9.828	29.877	13.523	1.00	0.78	O
ATOM	1022	CB	ALA	A	266	−10.677	26.981	15.091	1.00	0.78	C
ATOM	1023	N	PHE	A	267	−9.131	27.962	12.560	1.00	0.75	N
ATOM	1024	CA	PHE	A	267	−7.967	28.563	11.890	1.00	0.75	C
ATOM	1025	C	PHE	A	267	−8.411	29.527	10.783	1.00	0.75	C
ATOM	1026	O	PHE	A	267	−7.904	30.639	10.699	1.00	0.75	O
ATOM	1027	CB	PHE	A	267	−7.063	27.478	11.299	1.00	0.75	C

ATOM	1028	CG	PHE	A	267	−5.825	28.053	10.624	1.00	0.75	C
ATOM	1029	CD1	PHE	A	267	−5.887	28.480	9.304	1.00	0.75	C
ATOM	1030	CD2	PHE	A	267	−4.677	28.227	11.379	1.00	0.75	C
ATOM	1031	CE1	PHE	A	267	−4.788	29.107	8.738	1.00	0.75	C
ATOM	1032	CE2	PHE	A	267	−3.578	28.854	10.811	1.00	0.75	C
ATOM	1033	CZ	PHE	A	267	−3.636	29.296	9.494	1.00	0.75	C
ATOM	1034	N	ARG	A	268	−9.396	29.106	9.995	1.00	0.68	N
ATOM	1035	CA	ARG	A	268	−9.847	29.902	8.836	1.00	0.68	C
ATOM	1036	C	ARG	A	268	−10.483	31.253	9.208	1.00	0.68	C
ATOM	1037	O	ARG	A	268	−10.300	32.218	8.474	1.00	0.68	O
ATOM	1038	CB	ARG	A	268	−10.771	29.096	7.924	1.00	0.68	C
ATOM	1039	CG	ARG	A	268	−12.062	28.774	8.653	1.00	0.68	C
ATOM	1040	CD	ARG	A	268	−13.101	28.051	7.828	1.00	0.68	C
ATOM	1041	NE	ARG	A	268	−12.630	26.713	7.493	1.00	0.68	N
ATOM	1042	CZ	ARG	A	268	−13.291	25.905	6.678	1.00	0.68	C
ATOM	1043	NH1	ARG	A	268	−14.469	26.247	6.168	1.00	0.68	N
ATOM	1044	NH2	ARG	A	268	−12.729	24.784	6.284	1.00	0.68	N
ATOM	1045	N	PHE	A	269	−11.119	31.323	10.379	1.00	0.66	N
ATOM	1046	CA	PHE	A	269	−11.782	32.559	10.838	1.00	0.66	C
ATOM	1047	C	PHE	A	269	−11.013	33.281	11.951	1.00	0.66	C
ATOM	1048	O	PHE	A	269	−11.525	34.214	12.574	1.00	0.66	O
ATOM	1049	CB	PHE	A	269	−13.210	32.263	11.313	1.00	0.66	C
ATOM	1050	CG	PHE	A	269	−14.150	31.902	10.163	1.00	0.66	C
ATOM	1051	CD1	PHE	A	269	−14.793	32.889	9.429	1.00	0.66	C
ATOM	1052	CD2	PHE	A	269	−14.371	30.573	9.852	1.00	0.66	C
ATOM	1053	CE1	PHE	A	269	−15.635	32.539	8.379	1.00	0.66	C
ATOM	1054	CE2	PHE	A	269	−15.210	30.222	8.804	1.00	0.66	C
ATOM	1055	CZ	PHE	A	269	−15.837	31.203	8.056	1.00	0.66	C
ATOM	1056	N	TYR	A	270	−9.754	32.885	12.149	1.00	0.77	N
ATOM	1057	CA	TYR	A	270	−8.851	33.590	13.072	1.00	0.77	C
ATOM	1058	C	TYR	A	270	−8.795	35.073	12.675	1.00	0.77	C
ATOM	1059	O	TYR	A	270	−8.621	35.412	11.506	1.00	0.77	O
ATOM	1060	CB	TYR	A	270	−7.455	32.963	12.995	1.00	0.77	C
ATOM	1061	CG	TYR	A	270	−6.402	33.830	13.689	1.00	0.77	C
ATOM	1062	CD1	TYR	A	270	−6.177	33.728	15.054	1.00	0.77	C
ATOM	1063	CD2	TYR	A	270	−5.724	34.782	12.938	1.00	0.77	C
ATOM	1064	CE1	TYR	A	270	−5.253	34.565	15.668	1.00	0.77	C
ATOM	1065	CE2	TYR	A	270	−4.811	35.627	13.550	1.00	0.77	C
ATOM	1066	CZ	TYR	A	270	−4.566	35.508	14.909	1.00	0.77	C
ATOM	1067	OH	TYR	A	270	−3.581	36.260	15.462	1.00	0.77	O
ATOM	1068	N	ASP	A	271	−8.876	35.914	13.697	1.00	0.79	N
ATOM	1069	CA	ASP	A	271	−8.845	37.372	13.488	1.00	0.79	C

ATOM	1070	C	ASP	A	271	−7.761	38.100	14.286	1.00	0.79	C
ATOM	1071	O	ASP	A	271	−7.092	38.981	13.754	1.00	0.79	O
ATOM	1072	CB	ASP	A	271	−10.226	37.987	13.744	1.00	0.79	C
ATOM	1073	CG	ASP	A	271	−11.289	37.525	12.736	1.00	0.79	C
ATOM	1074	OD1	ASP	A	271	−10.912	37.118	11.616	1.00	0.79	O
ATOM	1075	OD2	ASP	A	271	−12.477	37.670	13.095	1.00	0.79	O
ATOM	1076	N	SER	A	272	−7.592	37.711	15.550	1.00	0.87	N
ATOM	1077	CA	SER	A	272	−6.621	38.367	16.445	1.00	0.87	C
ATOM	1078	C	SER	A	272	−6.302	37.519	17.680	1.00	0.87	C
ATOM	1079	O	SER	A	272	−7.010	36.564	18.005	1.00	0.87	O
ATOM	1080	CB	SER	A	272	−7.159	39.737	16.881	1.00	0.87	C
ATOM	1081	OG	SER	A	272	−6.176	40.432	17.654	1.00	0.87	O
ATOM	1082	N	GLY	A	273	−5.170	37.881	18.287	1.00	0.92	N
ATOM	1083	CA	GLY	A	273	−4.626	37.242	19.496	1.00	0.92	C
ATOM	1084	C	GLY	A	273	−3.722	36.066	19.121	1.00	0.92	C
ATOM	1085	O	GLY	A	273	−3.337	35.881	17.967	1.00	0.92	O
ATOM	1086	N	VAL	A	274	−3.459	35.240	20.119	1.00	0.88	N
ATOM	1087	CA	VAL	A	274	−2.655	34.017	19.940	1.00	0.88	C
ATOM	1088	C	VAL	A	274	−3.624	32.845	19.744	1.00	0.88	C
ATOM	1089	O	VAL	A	274	−4.318	32.432	20.673	1.00	0.88	O
ATOM	1090	CB	VAL	A	274	−1.722	33.784	21.145	1.00	0.88	C
ATOM	1091	CG1	VAL	A	274	−0.913	32.492	20.986	1.00	0.88	C
ATOM	1092	CG2	VAL	A	274	−0.746	34.951	21.332	1.00	0.88	C
ATOM	1093	N	LEU	A	275	−3.675	32.364	18.509	1.00	0.86	N
ATOM	1094	CA	LEU	A	275	−4.470	31.180	18.154	1.00	0.86	C
ATOM	1095	C	LEU	A	275	−3.914	29.972	18.922	1.00	0.86	C
ATOM	1096	O	LEU	A	275	−2.731	29.633	18.839	1.00	0.86	O
ATOM	1097	CB	LEU	A	275	−4.426	30.984	16.632	1.00	0.86	C
ATOM	1098	CG	LEU	A	275	−5.389	29.900	16.126	1.00	0.86	C
ATOM	1099	CD1	LEU	A	275	−5.738	30.164	14.663	1.00	0.86	C
ATOM	1100	CD2	LEU	A	275	−4.754	28.509	16.195	1.00	0.86	C
ATOM	1101	N	GLY	A	276	−4.845	29.341	19.637	1.00	0.87	N
ATOM	1102	CA	GLY	A	276	−4.558	28.167	20.480	1.00	0.87	C
ATOM	1103	C	GLY	A	276	−5.251	26.900	19.968	1.00	0.87	C
ATOM	1104	O	GLY	A	276	−5.182	25.841	20.559	1.00	0.87	O
ATOM	1105	N	GLY	A	277	−6.105	27.065	18.965	1.00	0.84	N
ATOM	1106	CA	GLY	A	277	−6.864	25.942	18.413	1.00	0.84	C
ATOM	1107	C	GLY	A	277	−8.278	26.394	18.037	1.00	0.84	C
ATOM	1108	O	GLY	A	277	−8.604	27.578	18.110	1.00	0.84	O
ATOM	1109	N	ALA	A	278	−9.087	25.461	17.550	1.00	0.81	N
ATOM	1110	CA	ALA	A	278	−8.732	24.035	17.392	1.00	0.81	C
ATOM	1111	C	ALA	A	278	−8.414	23.698	15.933	1.00	0.81	C

ATOM	1112	O	ALA	A	278	−9.039	24.231	15.014	1.00	0.81	O
ATOM	1113	CB	ALA	A	278	−9.873	23.163	17.917	1.00	0.81	C
ATOM	1114	N	CYS	A	279	−7.435	22.808	15.786	1.00	0.85	N
ATOM	1115	CA	CYS	A	279	−6.926	22.331	14.492	1.00	0.85	C
ATOM	1116	C	CYS	A	279	−6.339	20.932	14.566	1.00	0.85	C
ATOM	1117	O	CYS	A	279	−5.717	20.591	15.571	1.00	0.85	O
ATOM	1118	CB	CYS	A	279	−5.871	23.285	13.918	1.00	0.85	C
ATOM	1119	SG	CYS	A	279	−4.437	23.595	14.991	1.00	0.85	S
ATOM	1120	N	GLY	A	280	−6.580	20.160	13.498	1.00	0.88	N
ATOM	1121	CA	GLY	A	280	−5.980	18.823	13.369	1.00	0.88	C
ATOM	1122	C	GLY	A	280	−4.495	18.933	12.994	1.00	0.88	C
ATOM	1123	O	GLY	A	280	−3.914	20.015	12.939	1.00	0.88	O
ATOM	1124	N	THR	A	281	−3.949	17.794	12.589	1.00	0.88	N
ATOM	1125	CA	THR	A	281	−2.538	17.712	12.151	1.00	0.88	C
ATOM	1126	C	THR	A	281	−2.372	17.085	10.757	1.00	0.88	C
ATOM	1127	O	THR	A	281	−1.284	16.675	10.362	1.00	0.88	O
ATOM	1128	CB	THR	A	281	−1.702	16.926	13.173	1.00	0.88	C
ATOM	1129	OG1	THR	A	281	−2.303	15.643	13.369	1.00	0.88	O
ATOM	1130	CG2	THR	A	281	−1.556	17.678	14.502	1.00	0.88	C
ATOM	1131	N	ASP	A	282	−3.479	17.006	10.019	1.00	0.84	N
ATOM	1132	CA	ASP	A	282	−3.470	16.571	8.617	1.00	0.84	C
ATOM	1133	C	ASP	A	282	−3.057	17.755	7.732	1.00	0.84	C
ATOM	1134	O	ASP	A	282	−3.860	18.634	7.422	1.00	0.84	O
ATOM	1135	CB	ASP	A	282	−4.846	16.033	8.202	1.00	0.84	C
ATOM	1136	CG	ASP	A	282	−4.802	15.329	6.838	1.00	0.84	C
ATOM	1137	OD1	ASP	A	282	−3.731	15.360	6.192	1.00	0.84	O
ATOM	1138	OD2	ASP	A	282	−5.847	14.743	6.490	1.00	0.84	O
ATOM	1139	N	LEU	A	283	−1.781	17.756	7.383	1.00	0.84	N
ATOM	1140	CA	LEU	A	283	−1.161	18.848	6.609	1.00	0.84	C
ATOM	1141	C	LEU	A	283	−1.860	19.060	5.263	1.00	0.84	C
ATOM	1142	O	LEU	A	283	−2.107	18.110	4.522	1.00	0.84	O
ATOM	1143	CB	LEU	A	283	0.314	18.519	6.361	1.00	0.84	C
ATOM	1144	CG	LEU	A	283	1.093	18.257	7.656	1.00	0.84	C
ATOM	1145	CD1	LEU	A	283	2.515	17.811	7.318	1.00	0.84	C
ATOM	1146	CD2	LEU	A	283	1.119	19.488	8.566	1.00	0.84	C
ATOM	1147	N	ASN	A	284	−2.186	20.318	5.000	1.00	0.83	N
ATOM	1148	CA	ASN	A	284	−2.850	20.710	3.739	1.00	0.83	C
ATOM	1149	C	ASN	A	284	−2.517	22.124	3.239	1.00	0.83	C
ATOM	1150	O	ASN	A	284	−2.985	22.529	2.175	1.00	0.83	O
ATOM	1151	CB	ASN	A	284	−4.369	20.556	3.879	1.00	0.83	C
ATOM	1152	CG	ASN	A	284	−4.966	21.457	4.962	1.00	0.83	C
ATOM	1153	OD1	ASN	A	284	−4.496	22.524	5.339	1.00	0.83	O

ATOM	1154	ND2	ASN	A	284	−6.033	20.989	5.535	1.00	0.83 N
ATOM	1155	N	HIS	A	285	−1.812	22.896	4.062	1.00	0.82 N
ATOM	1156	CA	HIS	A	285	−1.500	24.294	3.741	1.00	0.82 C
ATOM	1157	C	HIS	A	285	−0.029	24.583	4.036	1.00	0.82 C
ATOM	1158	O	HIS	A	285	0.498	24.185	5.075	1.00	0.82 O
ATOM	1159	CB	HIS	A	285	−2.400	25.208	4.577	1.00	0.82 C
ATOM	1160	CG	HIS	A	285	−2.402	26.642	4.044	1.00	0.82 C
ATOM	1161	ND1	HIS	A	285	−2.327	27.007	2.767	1.00	0.82 N
ATOM	1162	CD2	HIS	A	285	−2.523	27.739	4.785	1.00	0.82 C
ATOM	1163	CE1	HIS	A	285	−2.411	28.337	2.723	1.00	0.82 C
ATOM	1164	NE2	HIS	A	285	−2.526	28.788	3.967	1.00	0.82 N
ATOM	1165	N	ALA	A	286	0.570	25.339	3.133	1.00	0.88 N
ATOM	1166	CA	ALA	A	286	1.978	25.739	3.237	1.00	0.88 C
ATOM	1167	C	ALA	A	286	2.091	27.245	3.464	1.00	0.88 C
ATOM	1168	O	ALA	A	286	1.558	28.051	2.700	1.00	0.88 O
ATOM	1169	CB	ALA	A	286	2.631	25.381	1.914	1.00	0.88 C
ATOM	1170	N	ILE	A	287	2.730	27.575	4.574	1.00	0.80 N
ATOM	1171	CA	ILE	A	287	2.940	28.971	5.014	1.00	0.80 C
ATOM	1172	C	ILE	A	287	4.404	29.152	5.467	1.00	0.80 C
ATOM	1173	O	ILE	A	287	5.225	28.248	5.327	1.00	0.80 O
ATOM	1174	CB	ILE	A	287	1.922	29.277	6.127	1.00	0.80 C
ATOM	1175	CG1	ILE	A	287	2.062	28.227	7.241	1.00	0.80 C
ATOM	1176	CG2	ILE	A	287	0.492	29.330	5.567	1.00	0.80 C
ATOM	1177	CD1	ILE	A	287	1.135	28.429	8.431	1.00	0.80 C
ATOM	1178	N	THR	A	288	4.685	30.228	6.192	1.00	0.87 N
ATOM	1179	CA	THR	A	288	6.056	30.492	6.696	1.00	0.87 C
ATOM	1180	C	THR	A	288	6.122	31.249	8.033	1.00	0.87 C
ATOM	1181	O	THR	A	288	5.450	32.265	8.234	1.00	0.87 O
ATOM	1182	CB	THR	A	288	6.836	31.309	5.669	1.00	0.87 C
ATOM	1183	OG1	THR	A	288	8.184	31.440	6.130	1.00	0.87 O
ATOM	1184	CG2	THR	A	288	6.165	32.675	5.468	1.00	0.87 C
ATOM	1185	N	ALA	A	289	6.888	30.697	8.955	1.00	0.94 N
ATOM	1186	CA	ALA	A	289	7.137	31.366	10.242	1.00	0.94 C
ATOM	1187	C	ALA	A	289	8.143	32.496	10.006	1.00	0.94 C
ATOM	1188	O	ALA	A	289	9.229	32.276	9.470	1.00	0.94 O
ATOM	1189	CB	ALA	A	289	7.682	30.360	11.255	1.00	0.94 C
ATOM	1190	N	VAL	A	290	7.750	33.696	10.401	1.00	0.87 N
ATOM	1191	CA	VAL	A	290	8.595	34.899	10.224	1.00	0.87 C
ATOM	1192	C	VAL	A	290	9.020	35.529	11.563	1.00	0.87 C
ATOM	1193	O	VAL	A	290	9.520	36.654	11.615	1.00	0.87 O
ATOM	1194	CB	VAL	A	290	7.913	35.934	9.305	1.00	0.87 C
ATOM	1195	CG1	VAL	A	290	7.661	35.397	7.896	1.00	0.87 C

ATOM	1196CG2		VAL	A	290	6.562	36.374	9.840	1.00	0.87	C
ATOM	1197N		GLY	A	291	8.764	34.790	12.647	1.00	0.96	N
ATOM	1198CA		GLY	A	291	9.179	35.213	13.986	1.00	0.96	C
ATOM	1199C		GLY	A	291	8.590	34.335	15.085	1.00	0.96	C
ATOM	1200O		GLY	A	291	7.800	33.424	14.832	1.00	0.96	O
ATOM	1201	N	TYR	A	292	8.962	34.707	16.297	1.00	0.88	N
ATOM	1202	CA	TYR	A	292	8.496	34.073	17.539	1.00	0.88	C
ATOM	1203	C	TYR	A	292	8.719	35.058	18.691	1.00	0.88	C
ATOM	1204	O	TYR	A	292	9.539	35.974	18.590	1.00	0.88	O
ATOM	1205	CB	TYR	A	292	9.255	32.762	17.805	1.00	0.88	C
ATOM	1206	CG	TYR	A	292	10.735	32.969	18.142	1.00	0.88	C
ATOM	1207	CD1	TYR	A	292	11.107	33.266	19.449	1.00	0.88	C
ATOM	1208	CD2	TYR	A	292	11.707	32.803	17.168	1.00	0.88	C
ATOM	1209	CE1	TYR	A	292	12.446	33.391	19.784	1.00	0.88	C
ATOM	1210	CE2	TYR	A	292	13.049	32.917	17.501	1.00	0.88	C
ATOM	1211	CZ	TYR	A	292	13.418	33.211	18.808	1.00	0.88	C
ATOM	1212	OH	TYR	A	292	14.731	33.320	19.130	1.00	0.88	O
ATOM	1213	N	GLY	A	293	8.008	34.786	19.783	1.00	0.89	N
ATOM	1214	CA	GLY	A	293	8.151	35.568	21.018	1.00	0.89	C
ATOM	1215	C	GLY	A	293	7.217	35.028	22.094	1.00	0.89	C
ATOM	1216	O	GLY	A	293	6.751	33.888	22.036	1.00	0.89	O
ATOM	1217	N	THR	A	294	6.969	35.885	23.068	1.00	0.76	N
ATOM	1218	CA	THR	A	294	6.100	35.556	24.209	1.00	0.76	C
ATOM	1219	C	THR	A	294	5.296	36.811	24.545	1.00	0.76	C
ATOM	1220	O	THR	A	294	5.862	37.894	24.688	1.00	0.76	O
ATOM	1221	CB	THR	A	294	6.958	35.127	25.409	1.00	0.76	C
ATOM	1222	OG1	THR	A	294	7.796	34.034	25.025	1.00	0.76	O
ATOM	1223	CG2	THR	A	294	6.111	34.698	26.606	1.00	0.76	C
ATOM	1224	N	ALA	A	295	3.979	36.648	24.595	1.00	0.72	N
ATOM	1225	CA	ALA	A	295	3.080	37.749	24.993	1.00	0.72	C
ATOM	1226	C	ALA	A	295	3.432	38.190	26.423	1.00	0.72	C
ATOM	1227	O	ALA	A	295	4.012	37.416	27.183	1.00	0.72	O
ATOM	1228	CB	ALA	A	295	1.625	37.279	24.914	1.00	0.72	C
ATOM	1229	N	ALA	A	296	3.013	39.396	26.798	1.00	0.61	N
ATOM	1230	CA	ALA	A	296	3.234	39.917	28.164	1.00	0.61	C
ATOM	1231	C	ALA	A	296	2.671	38.973	29.241	1.00	0.61	C
ATOM	1232	O	ALA	A	296	3.291	38.757	30.276	1.00	0.61	O
ATOM	1233	CB	ALA	A	296	2.584	41.295	28.305	1.00	0.61	C
ATOM	1234	N	ASP	A	297	1.574	38.300	28.887	1.00	0.57	N
ATOM	1235	CA	ASP	A	297	0.938	37.293	29.758	1.00	0.57	C
ATOM	1236	C	ASP	A	297	1.661	35.938	29.836	1.00	0.57	C
ATOM	1237	O	ASP	A	297	1.202	35.033	30.531	1.00	0.57	O

ATOM	1238	CB	ASP	A	297	−0.543	37.130	29.400	1.00	0.57	C
ATOM	1239	CG	ASP	A	297	−1.368	38.374	29.759	1.00	0.57	C
ATOM	1240	OD1	ASP	A	297	−0.861	39.233	30.515	1.00	0.57	O
ATOM	1241	OD2	ASP	A	297	−2.492	38.461	29.221	1.00	0.57	O
ATOM	1242	N	GLY	A	298	2.732	35.804	29.047	1.00	0.67	N
ATOM	1243	CA	GLY	A	298	3.596	34.611	29.080	1.00	0.67	C
ATOM	1244	C	GLY	A	298	3.168	33.483	28.130	1.00	0.67	C
ATOM	1245	O	GLY	A	298	3.598	32.345	28.297	1.00	0.67	O
ATOM	1246	N	THR	A	299	2.442	33.832	27.071	1.00	0.73	N
ATOM	1247	CA	THR	A	299	2.039	32.853	26.040	1.00	0.73	C
ATOM	1248	C	THR	A	299	3.053	32.897	24.894	1.00	0.73	C
ATOM	1249	O	THR	A	299	3.149	33.901	24.184	1.00	0.73	O
ATOM	1250	CB	THR	A	299	0.632	33.146	25.491	1.00	0.73	C
ATOM	1251	OG1	THR	A	299	−0.295	33.240	26.576	1.00	0.73	O
ATOM	1252	CG2	THR	A	299	0.167	32.071	24.502	1.00	0.73	C
ATOM	1253	N	ARG	A	300	3.815	31.816	24.775	1.00	0.72	N
ATOM	1254	CA	ARG	A	300	4.763	31.643	23.657	1.00	0.72	C
ATOM	1255	C	ARG	A	300	3.996	31.465	22.342	1.00	0.72	C
ATOM	1256	O	ARG	A	300	2.956	30.806	22.300	1.00	0.72	O
ATOM	1257	CB	ARG	A	300	5.644	30.417	23.888	1.00	0.72	C
ATOM	1258	CG	ARG	A	300	6.523	30.583	25.132	1.00	0.72	C
ATOM	1259	CD	ARG	A	300	7.442	29.384	25.367	1.00	0.72	C
ATOM	1260	NE	ARG	A	300	6.642	28.186	25.640	1.00	0.72	N
ATOM	1261	CZ	ARG	A	300	6.426	27.190	24.785	1.00	0.72	C
ATOM	1262	NH1	ARG	A	300	6.945	27.220	23.557	1.00	0.72	N
ATOM	1263	NH2	ARG	A	300	5.697	26.210	25.197	1.00	0.72	N
ATOM	1264	N	TYR	A	301	4.509	32.090	21.293	1.00	0.84	N
ATOM	1265	CA	TYR	A	301	3.870	32.040	19.965	1.00	0.84	C
ATOM	1266	C	TYR	A	301	4.884	32.029	18.816	1.00	0.84	C
ATOM	1267	O	TYR	A	301	6.037	32.432	18.980	1.00	0.84	O
ATOM	1268	CB	TYR	A	301	2.886	33.210	19.805	1.00	0.84	C
ATOM	1269	CG	TYR	A	301	3.593	34.566	19.812	1.00	0.84	C
ATOM	1270	CD1	TYR	A	301	4.107	35.079	18.628	1.00	0.84	C
ATOM	1271	CD2	TYR	A	301	3.723	35.275	20.997	1.00	0.84	C
ATOM	1272	CE1	TYR	A	301	4.754	36.305	18.626	1.00	0.84	C
ATOM	1273	CE2	TYR	A	301	4.361	36.506	20.998	1.00	0.84	C
ATOM	1274	CZ	TYR	A	301	4.873	37.019	19.811	1.00	0.84	C
ATOM	1275	OH	TYR	A	301	5.484	38.230	19.805	1.00	0.84	O
ATOM	1276	N	TRP	A	302	4.370	31.623	17.665	1.00	0.90	N
ATOM	1277	CA	TRP	A	302	5.044	31.749	16.365	1.00	0.90	C
ATOM	1278	C	TRP	A	302	4.253	32.788	15.572	1.00	0.90	C
ATOM	1279	O	TRP	A	302	3.026	32.869	15.665	1.00	0.90	O

ATOM	1280	CB	TRP	A	302	4.989	30.450	15.557	1.00	0.90	C
ATOM	1281	CG	TRP	A	302	5.585	29.236	16.262	1.00	0.90	C
ATOM	1282	CD1	TRP	A	302	4.922	28.163	16.693	1.00	0.90	C
ATOM	1283	CD2	TRP	A	302	6.935	28.944	16.328	1.00	0.90	C
ATOM	1284	NE1	TRP	A	302	5.793	27.211	17.028	1.00	0.90	N
ATOM	1285	CE2	TRP	A	302	7.029	27.653	16.821	1.00	0.90	C
ATOM	1286	CE3	TRP	A	302	8.074	29.681	16.032	1.00	0.90	C
ATOM	1287	CZ2	TRP	A	302	8.277	27.091	17.046	1.00	0.90	C
ATOM	1288	CZ3	TRP	A	302	9.324	29.113	16.239	1.00	0.90	C
ATOM	1289	CH2	TRP	A	302	9.416	27.823	16.748	1.00	0.90	C
ATOM	1290	N	LEU	A	303	4.984	33.571	14.805	1.00	0.91	N
ATOM	1291	CA	LEU	A	303	4.355	34.560	13.933	1.00	0.91	C
ATOM	1292	C	LEU	A	303	4.455	34.060	12.503	1.00	0.91	C
ATOM	1293	O	LEU	A	303	5.541	33.895	11.950	1.00	0.91	O
ATOM	1294	CB	LEU	A	303	5.102	35.864	14.087	1.00	0.91	C
ATOM	1295	CG	LEU	A	303	4.228	36.994	13.590	1.00	0.91	C
ATOM	1296	CD1	LEU	A	303	4.670	38.132	14.470	1.00	0.91	C
ATOM	1297	CD2	LEU	A	303	4.454	37.353	12.127	1.00	0.91	C
ATOM	1298	N	MET	A	304	3.283	33.811	11.955	1.00	0.87	N
ATOM	1299	CA	MET	A	304	3.177	33.185	10.645	1.00	0.87	C
ATOM	1300	C	MET	A	304	2.593	34.146	9.608	1.00	0.87	C
ATOM	1301	O	MET	A	304	1.529	34.722	9.835	1.00	0.87	O
ATOM	1302	CB	MET	A	304	2.254	32.001	10.873	1.00	0.87	C
ATOM	1303	CG	MET	A	304	2.034	31.290	9.558	1.00	0.87	C
ATOM	1304	SD	MET	A	304	3.556	30.462	9.014	1.00	0.87	S
ATOM	1305	CE	MET	A	304	3.947	29.397	10.367	1.00	0.87	C
ATOM	1306	N	LYS	A	305	3.194	34.094	8.425	1.00	0.84	N
ATOM	1307	CA	LYS	A	305	2.721	34.843	7.250	1.00	0.84	C
ATOM	1308	C	LYS	A	305	1.875	33.916	6.369	1.00	0.84	C
ATOM	1309	O	LYS	A	305	2.363	32.898	5.870	1.00	0.84	O
ATOM	1310	CB	LYS	A	305	3.927	35.372	6.469	1.00	0.84	C
ATOM	1311	CG	LYS	A	305	3.512	36.291	5.318	1.00	0.84	C
ATOM	1312	CD	LYS	A	305	4.737	36.764	4.540	1.00	0.84	C
ATOM	1313	CE	LYS	A	305	4.323	37.722	3.425	1.00	0.84	C
ATOM	1314	NZ	LYS	A	305	5.486	38.185	2.658	1.00	0.84	N
ATOM	1315	N	ASN	A	306	0.591	34.247	6.299	1.00	0.86	N
ATOM	1316	CA	ASN	A	306	−0.354	33.502	5.453	1.00	0.86	C
ATOM	1317	C	ASN	A	306	−0.470	34.195	4.084	1.00	0.86	C
ATOM	1318	O	ASN	A	306	−0.052	35.337	3.899	1.00	0.86	O
ATOM	1319	CB	ASN	A	306	−1.715	33.429	6.152	1.00	0.86	C
ATOM	1320	CG	ASN	A	306	−2.580	32.264	5.655	1.00	0.86	C
ATOM	1321	OD1	ASN	A	306	−2.279	31.540	4.712	1.00	0.86	O

ATOM	1322	ND2	ASN	A	306	−3.657	32.017	6.358	1.00 0.86	N
ATOM	1323	N	SER	A	307	−1.040	33.476	3.127	1.00 0.84	N
ATOM	1324	CA	SER	A	307	−1.199	33.957	1.740	1.00 0.84	C
ATOM	1325	C	SER	A	307	−2.675	34.135	1.344	1.00 0.84	C
ATOM	1326	O	SER	A	307	−3.068	33.932	0.192	1.00 0.84	O
ATOM	1327	CB	SER	A	307	−0.476	32.995	0.793	1.00 0.84	C
ATOM	1328	OG	SER	A	307	−0.988	31.678	1.002	1.00 0.84	O
ATOM	1329	N	TRP	A	308	−3.464	34.621	2.303	1.00 0.75	N
ATOM	1330	CA	TRP	A	308	−4.904	34.886	2.095	1.00 0.75	C
ATOM	1331	C	TRP	A	308	−5.253	36.380	2.136	1.00 0.75	C
ATOM	1332	O	TRP	A	308	−6.400	36.769	2.364	1.00 0.75	O
ATOM	1333	CB	TRP	A	308	−5.736	34.115	3.131	1.00 0.75	C
ATOM	1334	CG	TRP	A	308	−5.606	32.593	3.015	1.00 0.75	C
ATOM	1335	CD1	TRP	A	308	−5.120	31.901	1.985	1.00 0.75	C
ATOM	1336	CD2	TRP	A	308	−5.971	31.681	3.991	1.00 0.75	C
ATOM	1337	NE1	TRP	A	308	−5.156	30.601	2.263	1.00 0.75	N
ATOM	1338	CE2	TRP	A	308	−5.669	30.429	3.479	1.00 0.75	C
ATOM	1339	CE3	TRP	A	308	−6.573	31.810	5.236	1.00 0.75	C
ATOM	1340	CZ2	TRP	A	308	−5.971	29.293	4.218	1.00 0.75	C
ATOM	1341	CZ3	TRP	A	308	−6.885	30.672	5.972	1.00 0.75	C
ATOM	1342	CH2	TRP	A	308	−6.583	29.414	5.461	1.00 0.75	C
ATOM	1343	N	GLY	A	309	−4.249	37.203	1.802	1.00 0.82	N
ATOM	1344	CA	GLY	A	309	−4.409	38.666	1.768	1.00 0.82	C
ATOM	1345	C	GLY	A	309	−4.291	39.274	3.170	1.00 0.82	C
ATOM	1346	O	GLY	A	309	−4.491	38.617	4.190	1.00 0.82	O
ATOM	1347	N	ALA	A	310	−4.018	40.573	3.165	1.00 0.81	N
ATOM	1348	CA	ALA	A	310	−3.881	41.377	4.398	1.00 0.81	C
ATOM	1349	C	ALA	A	310	−5.175	41.429	5.229	1.00 0.81	C
ATOM	1350	O	ALA	A	310	−5.148	41.705	6.424	1.00 0.81	O
ATOM	1351	CB	ALA	A	310	−3.441	42.796	4.034	1.00 0.81	C
ATOM	1352	N	SER	A	311	−6.296	41.174	4.553	1.00 0.78	N
ATOM	1353	CA	SER	A	311	−7.644	41.168	5.157	1.00 0.78	C
ATOM	1354	C	SER	A	311	−7.843	40.056	6.204	1.00 0.78	C
ATOM	1355	O	SER	A	311	−8.707	40.160	7.073	1.00 0.78	O
ATOM	1356	CB	SER	A	311	−8.701	41.038	4.056	1.00 0.78	C
ATOM	1357	OG	SER	A	311	−10.006	41.180	4.622	1.00 0.78	O
ATOM	1358	N	TRP	A	312	−7.043	38.995	6.103	1.00 0.78	N
ATOM	1359	CA	TRP	A	312	−7.153	37.836	7.006	1.00 0.78	C
ATOM	1360	C	TRP	A	312	−6.243	37.995	8.231	1.00 0.78	C
ATOM	1361	O	TRP	A	312	−5.128	38.504	8.135	1.00 0.78	O
ATOM	1362	CB	TRP	A	312	−6.790	36.568	6.231	1.00 0.78	C
ATOM	1363	CG	TRP	A	312	−6.940	35.314	7.095	1.00 0.78	C

ATOM	1364	CD1	TRP	A	312	−8.057	34.615	7.272	1.00	0.78	C
ATOM	1365	CD2	TRP	A	312	−5.947	34.715	7.856	1.00	0.78	C
ATOM	1366	NE1	TRP	A	312	−7.817	33.597	8.096	1.00	0.78	N
ATOM	1367	CE2	TRP	A	312	−6.540	33.625	8.471	1.00	0.78	C
ATOM	1368	CE3	TRP	A	312	−4.599	34.993	8.053	1.00	0.78	C
ATOM	1369	CZ2	TRP	A	312	−5.776	32.792	9.277	1.00	0.78	C
ATOM	1370	CZ3	TRP	A	312	−3.836	34.155	8.856	1.00	0.78	C
ATOM	1371	CH2	TRP	A	312	−4.426	33.052	9.463	1.00	0.78	C
ATOM	1372	N	GLY	A	313	−6.740	37.508	9.373	1.00	0.86	N
ATOM	1373	CA	GLY	A	313	−5.982	37.513	10.640	1.00	0.86	C
ATOM	1374	C	GLY	A	313	−5.512	38.923	11.022	1.00	0.86	C
ATOM	1375	O	GLY	A	313	−6.155	39.927	10.721	1.00	0.86	O
ATOM	1376	N	GLU	A	314	−4.322	38.975	11.605	1.00	0.82	N
ATOM	1377	CA	GLU	A	314	−3.685	40.245	12.002	1.00	0.82	C
ATOM	1378	C	GLU	A	314	−2.848	40.819	10.851	1.00	0.82	C
ATOM	1379	O	GLU	A	314	−1.674	40.480	10.678	1.00	0.82	O
ATOM	1380	CB	GLU	A	314	−2.801	40.033	13.228	1.00	0.82	C
ATOM	1381	CG	GLU	A	314	−3.592	39.487	14.416	1.00	0.82	C
ATOM	1382	CD	GLU	A	314	−2.719	39.327	15.661	1.00	0.82	C
ATOM	1383	OE1	GLU	A	314	−1.496	39.096	15.524	1.00	0.82	O
ATOM	1384	OE2	GLU	A	314	−3.301	39.431	16.762	1.00	0.82	O
ATOM	1385	N	GLY	A	315	−3.556	41.549	9.980	1.00	0.83	N
ATOM	1386	CA	GLY	A	315	−2.959	42.165	8.775	1.00	0.83	C
ATOM	1387	C	GLY	A	315	−2.303	41.150	7.821	1.00	0.83	C
ATOM	1388	O	GLY	A	315	−1.314	41.464	7.161	1.00	0.83	O
ATOM	1389	N	GLY	A	316	−2.798	39.908	7.862	1.00	0.86	N
ATOM	1390	CA	GLY	A	316	−2.307	38.817	6.994	1.00	0.86	C
ATOM	1391	C	GLY	A	316	−1.555	37.720	7.761	1.00	0.86	C
ATOM	1392	O	GLY	A	316	−1.289	36.641	7.229	1.00	0.86	O
ATOM	1393	N	TYR	A	317	−1.204	38.031	9.005	1.00	0.84	N
ATOM	1394	CA	TYR	A	317	−0.427	37.136	9.875	1.00	0.84	C
ATOM	1395	C	TYR	A	317	−1.312	36.529	10.965	1.00	0.84	C
ATOM	1396	O	TYR	A	317	−2.422	36.981	11.243	1.00	0.84	O
ATOM	1397	CB	TYR	A	317	0.721	37.902	10.541	1.00	0.84	C
ATOM	1398	CG	TYR	A	317	1.691	38.491	9.518	1.00	0.84	C
ATOM	1399	CD1	TYR	A	317	1.442	39.754	8.999	1.00	0.84	C
ATOM	1400	CD2	TYR	A	317	2.822	37.788	9.130	1.00	0.84	C
ATOM	1401	CE1	TYR	A	317	2.325	40.313	8.089	1.00	0.84	C
ATOM	1402	CE2	TYR	A	317	3.709	38.346	8.220	1.00	0.84	C
ATOM	1403	CZ	TYR	A	317	3.452	39.604	7.693	1.00	0.84	C
ATOM	1404	OH	TYR	A	317	4.297	40.149	6.785	1.00	0.84	O
ATOM	1405	N	VAL	A	318	−0.804	35.435	11.507	1.00	0.88	N

ATOM	1406	CA	VAL	A	318	−1.421	34.728	12.638	1.00	0.88	C
ATOM	1407	C	VAL	A	318	−0.331	34.386	13.659	1.00	0.88	C
ATOM	1408	O	VAL	A	318	0.727	33.862	13.304	1.00	0.88	O
ATOM	1409	CB	VAL	A	318	−2.185	33.484	12.132	1.00	0.88	C
ATOM	1410	CG1	VAL	A	318	−1.298	32.451	11.426	1.00	0.88	C
ATOM	1411	CG2	VAL	A	318	−2.971	32.799	13.252	1.00	0.88	C
ATOM	1412	N	ARG	A	319	−0.650	34.648	14.915	1.00	0.87	N
ATOM	1413	CA	ARG	A	319	0.220	34.233	16.025	1.00	0.87	C
ATOM	1414	C	ARG	A	319	−0.341	32.944	16.603	1.00	0.87	C
ATOM	1415	O	ARG	A	319	−1.488	32.894	17.040	1.00	0.87	O
ATOM	1416	CB	ARG	A	319	0.294	35.290	17.121	1.00	0.87	C
ATOM	1417	CG	ARG	A	319	1.100	36.501	16.652	1.00	0.87	C
ATOM	1418	CD	ARG	A	319	1.187	37.559	17.750	1.00	0.87	C
ATOM	1419	NE	ARG	A	319	−0.147	38.120	18.031	1.00	0.87	N
ATOM	1420	CZ	ARG	A	319	−0.581	38.567	19.211	1.00	0.87	C
ATOM	1421	NH1	ARG	A	319	0.191	38.537	20.289	1.00	0.87	N
ATOM	1422	NH2	ARG	A	319	−1.798	39.080	19.315	1.00	0.87	N
ATOM	1423	N	ILE	A	320	0.422	31.886	16.425	1.00	0.85	N
ATOM	1424	CA	ILE	A	320	−0.017	30.554	16.864	1.00	0.85	C
ATOM	1425	C	ILE	A	320	0.796	30.159	18.098	1.00	0.85	C
ATOM	1426	O	ILE	A	320	2.007	30.374	18.149	1.00	0.85	O
ATOM	1427	CB	ILE	A	320	0.169	29.552	15.722	1.00	0.85	C
ATOM	1428	CG1	ILE	A	320	1.645	29.479	15.344	1.00	0.85	C
ATOM	1429	CG2	ILE	A	320	−0.708	29.925	14.510	1.00	0.85	C
ATOM	1430	CD1	ILE	A	320	1.858	28.283	14.459	1.00	0.85	C
ATOM	1431	N	ARG	A	321	0.120	29.561	19.064	1.00	0.80	N
ATOM	1432	CA	ARG	A	321	0.785	29.102	20.296	1.00	0.80	C
ATOM	1433	C	ARG	A	321	1.985	28.195	19.975	1.00	0.80	C
ATOM	1434	O	ARG	A	321	1.935	27.323	19.104	1.00	0.80	O
ATOM	1435	CB	ARG	A	321	−0.221	28.391	21.203	1.00	0.80	C
ATOM	1436	CG	ARG	A	321	−0.830	27.151	20.545	1.00	0.80	C
ATOM	1437	CD	ARG	A	321	−1.742	26.456	21.540	1.00	0.80	C
ATOM	1438	NE	ARG	A	321	−2.281	25.214	20.961	1.00	0.80	N
ATOM	1439	CZ	ARG	A	321	−3.164	24.420	21.571	1.00	0.80	C
ATOM	1440	NH1	ARG	A	321	−3.665	24.747	22.760	1.00	0.80	N
ATOM	1441	NH2	ARG	A	321	−3.675	23.371	20.933	1.00	0.80	N
ATOM	1442	N	ARG	A	322	3.076	28.483	20.659	1.00	0.79	N
ATOM	1443	CA	ARG	A	322	4.354	27.782	20.463	1.00	0.79	C
ATOM	1444	C	ARG	A	322	4.661	26.920	21.691	1.00	0.79	C
ATOM	1445	O	ARG	A	322	4.165	27.208	22.776	1.00	0.79	O
ATOM	1446	CB	ARG	A	322	5.426	28.843	20.216	1.00	0.79	C
ATOM	1447	CG	ARG	A	322	6.843	28.279	20.094	1.00	0.79	C

ATOM	1448	CD	ARG	A	322	7.824	29.345	19.608	1.00	0.79	C
ATOM	1449	NE	ARG	A	322	7.917	30.468	20.550	1.00	0.79	N
ATOM	1450	CZ	ARG	A	322	8.621	30.473	21.681	1.00	0.79	C
ATOM	1451	NH1	ARG	A	322	9.315	29.409	22.071	1.00	0.79	N
ATOM	1452	NH2	ARG	A	322	8.677	31.575	22.412	1.00	0.79	N
ATOM	1453	N	GLY	A	323	5.360	25.807	21.410	1.00	0.80	N
ATOM	1454	CA	GLY	A	323	5.779	24.774	22.386	1.00	0.80	C
ATOM	1455	C	GLY	A	323	4.604	24.177	23.171	1.00	0.80	C
ATOM	1456	O	GLY	A	323	4.614	23.950	24.372	1.00	0.80	O
ATOM	1457	N	VAL	A	324	3.667	23.693	22.393	1.00	0.75	N
ATOM	1458	CA	VAL	A	324	2.491	23.038	22.978	1.00	0.75	C
ATOM	1459	C	VAL	A	324	2.589	21.514	22.846	1.00	0.75	C
ATOM	1460	O	VAL	A	324	2.460	20.770	23.813	1.00	0.75	O
ATOM	1461	CB	VAL	A	324	1.202	23.657	22.406	1.00	0.75	C
ATOM	1462	CG1	VAL	A	324	1.072	23.545	20.880	1.00	0.75	C
ATOM	1463	CG2	VAL	A	324	−0.025	23.090	23.124	1.00	0.75	C
ATOM	1464	N	ARG	A	325	2.761	21.081	21.607	1.00	0.68	N
ATOM	1465	CA	ARG	A	325	2.767	19.653	21.276	1.00	0.68	C
ATOM	1466	C	ARG	A	325	3.966	19.293	20.403	1.00	0.68	C
ATOM	1467	O	ARG	A	325	4.493	20.134	19.676	1.00	0.68	O
ATOM	1468	CB	ARG	A	325	1.435	19.240	20.637	1.00	0.68	C
ATOM	1469	CG	ARG	A	325	1.124	20.034	19.373	1.00	0.68	C
ATOM	1470	CD	ARG	A	325	−0.138	19.564	18.674	1.00	0.68	C
ATOM	1471	NE	ARG	A	325	−1.281	19.921	19.520	1.00	0.68	N
ATOM	1472	CZ	ARG	A	325	−2.536	19.561	19.284	1.00	0.68	C
ATOM	1473	NH1	ARG	A	325	−2.848	18.830	18.220	1.00	0.68	N
ATOM	1474	NH2	ARG	A	325	−3.489	19.921	20.129	1.00	0.68	N
ATOM	1475	N	GLY	A	326	4.289	17.999	20.453	1.00	0.75	N
ATOM	1476	CA	GLY	A	326	5.398	17.406	19.677	1.00	0.75	C
ATOM	1477	C	GLY	A	326	5.276	17.679	18.171	1.00	0.75	C
ATOM	1478	O	GLY	A	326	6.210	18.173	17.553	1.00	0.75	O
ATOM	1479	N	GLU	A	327	4.070	17.500	17.638	1.00	0.72	N
ATOM	1480	CA	GLU	A	327	3.749	17.854	16.236	1.00	0.72	C
ATOM	1481	C	GLU	A	327	3.628	19.373	16.031	1.00	0.72	C
ATOM	1482	O	GLU	A	327	3.736	19.883	14.916	1.00	0.72	O
ATOM	1483	CB	GLU	A	327	2.461	17.181	15.739	1.00	0.72	C
ATOM	1484	CG	GLU	A	327	2.537	15.648	15.751	1.00	0.72	C
ATOM	1485	CD	GLU	A	327	2.260	14.998	17.113	1.00	0.72	C
ATOM	1486	OE1	GLU	A	327	2.069	15.736	18.112	1.00	0.72	O
ATOM	1487	OE2	GLU	A	327	2.171	13.756	17.124	1.00	0.72	O
ATOM	1488	N	GLY	A	328	3.397	20.088	17.136	1.00	0.83	N
ATOM	1489	CA	GLY	A	328	3.170	21.547	17.112	1.00	0.83	C

ATOM	1490	C	GLY	A	328	1.711	21.824	16.730	1.00 0.83	C
ATOM	1491	O	GLY	A	328	1.006	20.961	16.204	1.00 0.83	O
ATOM	1492	N	VAL	A	329	1.250	23.018	17.077	1.00 0.86	N
ATOM	1493	CA	VAL	A	329	−0.124	23.463	16.762	1.00 0.86	C
ATOM	1494	C	VAL	A	329	−0.394	23.348	15.252	1.00 0.86	C
ATOM	1495	O	VAL	A	329	0.412	23.789	14.431	1.00 0.86	O
ATOM	1496	CB	VAL	A	329	−0.352	24.900	17.265	1.00 0.86	C
ATOM	1497	CG1	VAL	A	329	0.586	25.914	16.609	1.00 0.86	C
ATOM	1498	CG2	VAL	A	329	−1.813	25.332	17.100	1.00 0.86	C
ATOM	1499	N	CYS	A	330	−1.476	22.650	14.931	1.00 0.91	N
ATOM	1500	CA	CYS	A	330	−1.893	22.395	13.533	1.00 0.91	C
ATOM	1501	C	CYS	A	330	−0.800	21.643	12.747	1.00 0.91	C
ATOM	1502	O	CYS	A	330	−0.760	21.670	11.517	1.00 0.91	O
ATOM	1503	CB	CYS	A	330	−2.267	23.717	12.833	1.00 0.91	C
ATOM	1504	SG	CYS	A	330	−3.323	24.840	13.823	1.00 0.91	S
ATOM	1505	N	GLY	A	331	0.066	20.956	13.508	1.00 0.97	N
ATOM	1506	CA	GLY	A	331	1.201	20.181	12.977	1.00 0.97	C
ATOM	1507	C	GLY	A	331	2.273	21.054	12.313	1.00 0.97	C
ATOM	1508	O	GLY	A	331	3.023	20.568	11.470	1.00 0.97	O
ATOM	1509	N	LEU	A	332	2.455	22.268	12.835	1.00 0.87	N
ATOM	1510	CA	LEU	A	332	3.449	23.212	12.293	1.00 0.87	C
ATOM	1511	C	LEU	A	332	4.897	22.675	12.320	1.00 0.87	C
ATOM	1512	O	LEU	A	332	5.734	23.075	11.510	1.00 0.87	O
ATOM	1513	CB	LEU	A	332	3.313	24.566	13.002	1.00 0.87	C
ATOM	1514	CG	LEU	A	332	4.324	25.545	12.403	1.00 0.87	C
ATOM	1515	CD1	LEU	A	332	3.894	26.968	12.505	1.00 0.87	C
ATOM	1516	CD2	LEU	A	332	5.566	25.599	13.260	1.00 0.87	C
ATOM	1517	N	ALA	A	333	5.191	21.811	13.286	1.00 0.83	N
ATOM	1518	CA	ALA	A	333	6.548	21.254	13.454	1.00 0.83	C
ATOM	1519	C	ALA	A	333	6.815	19.976	12.637	1.00 0.83	C
ATOM	1520	O	ALA	A	333	7.924	19.443	12.670	1.00 0.83	O
ATOM	1521	CB	ALA	A	333	6.800	21.004	14.942	1.00 0.83	C
ATOM	1522	N	LYS	A	334	5.799	19.497	11.919	1.00 0.76	N
ATOM	1523	CA	LYS	A	334	5.883	18.251	11.135	1.00 0.76	C
ATOM	1524	C	LYS	A	334	6.776	18.346	9.881	1.00 0.76	C
ATOM	1525	O	LYS	A	334	7.732	17.597	9.734	1.00 0.76	O
ATOM	1526	CB	LYS	A	334	4.474	17.804	10.735	1.00 0.76	C
ATOM	1527	CG	LYS	A	334	3.590	17.352	11.902	1.00 0.76	C
ATOM	1528	CD	LYS	A	334	4.062	16.066	12.578	1.00 0.76	C
ATOM	1529	CE	LYS	A	334	4.138	14.789	11.746	1.00 0.76	C
ATOM	1530	NZ	LYS	A	334	4.785	13.688	12.479	1.00 0.76	N
ATOM	1531	N	LEU	A	335	6.569	19.384	9.074	1.00 0.82	N

ATOM	1532	CA	LEU	A	335	7.336	19.519	7.815	1.00	0.82	C
ATOM	1533	C	LEU	A	335	7.898	20.949	7.632	1.00	0.82	C
ATOM	1534	O	LEU	A	335	7.373	21.744	6.854	1.00	0.82	O
ATOM	1535	CB	LEU	A	335	6.450	19.025	6.660	1.00	0.82	C
ATOM	1536	CG	LEU	A	335	7.154	18.871	5.301	1.00	0.82	C
ATOM	1537	CD1	LEU	A	335	6.379	17.852	4.476	1.00	0.82	C
ATOM	1538	CD2	LEU	A	335	7.153	20.152	4.462	1.00	0.82	C
ATOM	1539	N	PRO	A	336	8.846	21.347	8.483	1.00	0.87	N
ATOM	1540	CA	PRO	A	336	9.509	22.647	8.320	1.00	0.87	C
ATOM	1541	C	PRO	A	336	10.826	22.475	7.561	1.00	0.87	C
ATOM	1542	O	PRO	A	336	11.562	21.513	7.754	1.00	0.87	O
ATOM	1543	CB	PRO	A	336	9.718	23.122	9.753	1.00	0.87	C
ATOM	1544	CG	PRO	A	336	9.955	21.835	10.541	1.00	0.87	C
ATOM	1545	CD	PRO	A	336	9.084	20.807	9.838	1.00	0.87	C
ATOM	1546	N	SER	A	337	11.156	23.463	6.750	1.00	0.85	N
ATOM	1547	CA	SER	A	337	12.389	23.458	5.940	1.00	0.85	C
ATOM	1548	C	SER	A	337	12.735	24.881	5.512	1.00	0.85	C
ATOM	1549	O	SER	A	337	11.893	25.780	5.532	1.00	0.85	O
ATOM	1550	CB	SER	A	337	12.235	22.576	4.697	1.00	0.85	C
ATOM	1551	OG	SER	A	337	11.191	23.067	3.851	1.00	0.85	O
ATOM	1552	N	TYR	A	338	14.011	25.067	5.213	1.00	0.79	N
ATOM	1553	CA	TYR	A	338	14.555	26.392	4.884	1.00	0.79	C
ATOM	1554	C	TYR	A	338	15.670	26.315	3.827	1.00	0.79	C
ATOM	1555	O	TYR	A	338	16.324	25.271	3.707	1.00	0.79	O
ATOM	1556	CB	TYR	A	338	15.057	27.074	6.167	1.00	0.79	C
ATOM	1557	CG	TYR	A	338	16.081	26.205	6.891	1.00	0.79	C
ATOM	1558	CD1	TYR	A	338	17.420	26.255	6.528	1.00	0.79	C
ATOM	1559	CD2	TYR	A	338	15.638	25.299	7.842	1.00	0.79	C
ATOM	1560	CE1	TYR	A	338	18.325	25.392	7.126	1.00	0.79	C
ATOM	1561	CE2	TYR	A	338	16.541	24.437	8.443	1.00	0.79	C
ATOM	1562	CZ	TYR	A	338	17.882	24.485	8.080	1.00	0.79	C
ATOM	1563	OH	TYR	A	338	18.763	23.620	8.642	1.00	0.79	O
ATOM	1564	N	PRO	A	339	15.823	27.397	3.050	1.00	0.78	N
ATOM	1565	CA	PRO	A	339	16.882	27.532	2.041	1.00	0.78	C
ATOM	1566	C	PRO	A	339	18.226	27.809	2.713	1.00	0.78	C
ATOM	1567	O	PRO	A	339	18.318	28.268	3.848	1.00	0.78	O
ATOM	1568	CB	PRO	A	339	16.439	28.722	1.187	1.00	0.78	C
ATOM	1569	CG	PRO	A	339	15.733	29.627	2.194	1.00	0.78	C
ATOM	1570	CD	PRO	A	339	14.999	28.625	3.081	1.00	0.78	C
ATOM	1571	N	VAL	A	340	19.263	27.458	1.981	1.00	0.66	N
ATOM	1572	CA	VAL	A	340	20.643	27.704	2.418	1.00	0.66	C
ATOM	1573	C	VAL	A	340	21.285	28.738	1.460	1.00	0.66	C

ATOM 1574 O VALA 340 21.072 28.541 0.232 1.00 0.66 O
ATOM 1575 CB VALA 340 21.394 26.361 2.579 1.00 0.66 C
ATOM 1576 CG1 VALA 340 20.800 25.435 3.638 1.00 0.66 C
ATOM 1577 CG2 VALA 340 21.404 25.497 1.341 1.00 0.66 C
ATOM 1578 OXTVALA 340 21.586 29.837 1.931 1.00 0.66 O
TER 1579 VALA 340
END

2. The Structure of R'-7-Amido-4-methylcoumarin (R'-AMC) and R"-R-AMC

Figure S1. (**a**) the structure of R'-7-Amido-4-methylcoumarin (R'-AMC); (**b**) the structure of R"-R-AMC. (R', R" refers to G, A, V, L, I, F, W, M, Y, S, T, N, Q, D, E, K, R, and H residues).

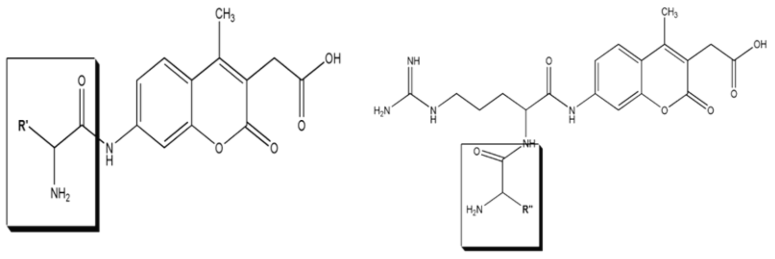

R'-AMC a R"-R-AMC b

© 2014 by the authors; licensee MDPI, Basel, Switzerland. This article is an open access article distributed under the terms and conditions of the Creative Commons Attribution license (http://creativecommons.org/licenses/by/3.0/).
